# Four novel algal virus genomes discovered from Yellowstone Lake metagenomes

**DOI:** 10.1038/srep15131

**Published:** 2015-10-13

**Authors:** Weijia Zhang, Jinglie Zhou, Taigang Liu, Yongxin Yu, Yingjie Pan, Shuling Yan, Yongjie Wang

**Affiliations:** 1 College of Food Science and Technology, Shanghai Ocean University, Shanghai, China; 2 Department of Biological Sciences, Auburn University, Auburn, AL, USA; 3 College of Information Technology, Shanghai Ocean University, Shanghai, China; 4 Laboratory of Quality and Safety Risk Assessment for Aquatic Products on Storage & Preservation, Ministry of Agriculture, Shanghai, China; 5 Institute of Biochemistry and Molecular Cell Biology, University of Göttingen, Göttingen, Germany

## Abstract

Phycodnaviruses are algae-infecting large dsDNA viruses that are widely distributed in aquatic environments. Here, partial genomic sequences of four novel algal viruses were assembled from a Yellowstone Lake metagenomic data set. Genomic analyses revealed that three Yellowstone Lake phycodnaviruses (YSLPVs) had genome lengths of 178,262 bp, 171,045 bp, and 171,454 bp, respectively, and were phylogenetically closely related to prasinoviruses (*Phycodnaviridae*). The fourth (YSLGV), with a genome length of 73,689 bp, was related to group III in the extended family *Mimiviridae* comprising Organic Lake phycodnaviruses and *Phaeocystis globosa* virus 16 T (OLPG). A pair of inverted terminal repeats was detected in YSLPV1, suggesting that its genome is nearly complete. Interestingly, these four putative YSL giant viruses also bear some genetic similarities to Yellowstone Lake virophages (YSLVs). For example, they share nine non-redundant homologous genes, including ribonucleotide reductase small subunit (a gene conserved in nucleo-cytoplasmic large DNA viruses) and Organic Lake virophage OLV2 (conserved in the majority of YSLVs). Additionally, putative multidrug resistance genes (emrE) were found in YSLPV1 and YSLPV2 but not in other viruses. Phylogenetic trees of emrE grouped YSLPVs with algae, suggesting that horizontal gene transfer occurred between giant viruses and their potential algal hosts.

Phytoplankton (microalgae), based on conservative estimates of more than 100,000 species^1^, is abundant in the sea. These algae form the base of the marine food web as their photosynthetic activities provide carbon sources and energy for life in the marine ecosystem and they regulate numerous aspects of the global environment^1^. Marine algae-infecting viruses, particularly phycodnaviruses, are important for controlling the composition of planktonic communities^2^.

The phycodnaviruses are a genetically diverse, morphologically similar group of double-stranded DNA viruses that infect eukaryotic algae and presently contain six genera^3^. Members of the *Coccolithovirus*, *Phaeovirus*, *Prasinovirus*, *Prymnesiovirus* and *Raphidovirus* genera infect marine algae, while chloroviruses (*Chlorovirus*) infect freshwater algae^1^,^4^. The genomes of phycodnaviruses are generally smaller (160 to 560 kb) than those of mimiviruses belonging to Lineage A of Group I, which typically have genomes larger than 1 Mb. Thus far, complete genomes of phycodnaviruses, such as *Ostreococcus* viruses (OtV1, OtV5)^5^,^6^, *Micromonas* sp. RCC1109 virus MpV1^7^ and five chloroviruses^8^,^9^,^10^, have been well characterized.

The *Phycodnaviridae*, together with six other giant virus families, were defined as nucleo-cytoplasmic large DNA viruses (NCLDVs)^11^ and were proposed to be reclassified into a new order *Megavirales* due to the following shared features^12^: i) giant viral particles with capsid diameters >150 nm and genome sizes >100 kb; ii) the presence of nine class I core genes in all seven families or 47 NCLDV conserved genes in one or two families^13^; iii) potential for infection with virophages.

Interestingly, virophages were found to be associated with giant viruses since the first virophage Sputnik was isolated in association with a mamavirus, a relative of mimivirus, in a water-cooling tower in Paris^14^. Virophages have circular double-stranded DNA genomes of 18–30 kb which encode more than 20 genes. Recently, Zhou *et al.*^15^,^16^ observed extensive genetic diversity of virophages in Yellowstone Lake metagenomic datasets and have assembled seven complete virophage genomes (Yellowstone Lake virophages, YSLVs).

In this study, to provide insight into the diversity of giant viruses in Yellowstone Lake, we assembled giant viral genomes from the same metagenomic datasets in which YSLVs were discovered^15^,^16^. Based on comparative genomic and phylogenetic analyses, four novel giant viruses detected in YSL appeared to infect algae, and horizontal gene transfer was observed among giant viruses, YSLVs, and their potential algae hosts.

## Material and Methods

### Sequence assembly

Sequence assembly was performed as previously described by Zhou *et al.*^15^,^16^. Briefly, the Yellowstone Lake metagenomics dataset^17^ was downloaded from the CAMERA 2.0 Portal and assembled *de novo* using Newbler v2.6 (Roche). Contigs derived from the assembly were constructed as a local database in order to perform tBLASTx searches for viral major capsid protein (MCP)-related sequences. Since YSLVs are most closely related to Organic Lake virophage (OLV)^15^,^16^, and OLV is thought to be the viral parasite of Organic Lake phycodnaviruses (OLPVs)^15^,^18^, the potential giant viral hosts of YSLVs may be closely related to OLPVs. Therefore, MCPs of OLPVs were used as reference sequences to search (tblastx, E-value < 10^−5^) for homologous sequences in the constructed contig database described above. The OLPV MCP-related contigs over 10 kb in length with good quality (low E-value and high identity) were re-assembled using the Yellowstone Lake metagenomic dataset until the assembled sequences no longer extended. All sequence assemblies (with a minimum overlap length of 25 bp and minimum overlap identity of 95%) were performed using GeneiousPro^15^.

### Assembly check

To further validate the assembled consensus sequences, duplicate reads were first removed from the data sets of scaffold reads with CD-HIT Suite^19^. Sequence identity cut-offs were set as: 0.97, 0.95 and 0.90. The obtained unique reads were then re-assembled, and the consensus sequences were compared to the original sequences using the data sets without prior removal of duplicates in order to check the quality and accuracy of sequence assembly.

### Genomic sequence analysis

The prediction and annotation of open reading frames (ORF) was performed as described by Zhou *et al.* in 2013^15^. Each predicted ORF contained an ATG start codon, and had a minimum size of 150 bp, standard genetic code, and a stop codon. Translated amino acid sequences were used to search (E-value < 10^−1^) for homologs in NCBI nr database using the BLASTp program. One top hit to virus and/or non-virus was recorded. Functional annotation of ORFs was performed using the InterProScan program (http://www.ebi.ac.uk/Tools/pfa/iprscan/), Conserved Domain search^20^ on the NCBI server, and HHpred (http://toolkit.tuebingen.mpg.de/hhpred).

In addition, 412 proteins of OLPV1, 326 of OLPV2 and 434 of *Phaeocystis globosa* virus 16 T (PgV-16 T) were analyzed for orthologous protein clusters shared with YSLGV proteins using the COG algorithm^21^.

All predicted ORFs (E-value < 10^−1^) were searched against a local database, which is comprised of all predicted proteins of seven YSLVs. Analysis of PgVV (a pro-virophage associated with PgV-16 T) proteins was performed by searching their homolog against an extensive database containing sequences of all the predicted proteins of known virophages.

Genomic sequences were aligned using the Mauve program on the Geneious Pro platform (default parameter)^22^. Repetitive sequences were checked on the softberry website (http://linux1.softberry.com/berry.phtml? topic = frep&subgroup = repeat&group = programs) with default parameters, and long terminal repeats using LTR-Finder^23^.

### Phylogenetic analysis

Homologs of MCP, DNA polymerase B family (PolB), poxvirus late transcription factor 3 (Pox-VLTF3), topoisomerase II (Topo II), vaccinia virus (VV) A32-like packaging ATPase, ribonucleotide reductase small subunit (RNR2), multidrug resistance protein (emrE), and OLV ORF2 (OLV2) were used to reconstruct phylogenetic trees. Reconstruction was initiated by aligning multiple amino acid sequences using the MUSCLE program^24^, followed by tree construction using the JTT model with a bootstrap value of 100. Phylogenetic analysis of emrE included sequences from three cellular life domains and was based on Bayesian Inference (parameter set: rate variation: gamma; rate matrix: poisson). All analyses were performed on the Geneious Pro platform.

### Accession numbers

The genomic sequences of four YSL algal viruses have been deposited in DDBJ under accession numbers LC015646-LC015649 for YSLGV and YSLPV 1–3, respectively.

## Results

### Genomic features

A total of 677,637 contigs were obtained (100–199,335 bp in length) after *de novo* assembly and were used to search OLPV MCP-related contigs with tBLASTx. Six contigs (10 kb <length <50 kb, E value < e^−80^) were obtained, four of which were ultimately extended to 178,262, 171,454, 171,045, and 73,689 bp, after reference assembly (Table S1, Fig. S1). Duplicate reads were then removed from each scaffold data set. Re-assembled consensus sequences exhibited >99% nt identity to their corresponding sequences as described above, confirming the accuracy of the assembly.

**Figure 1:**
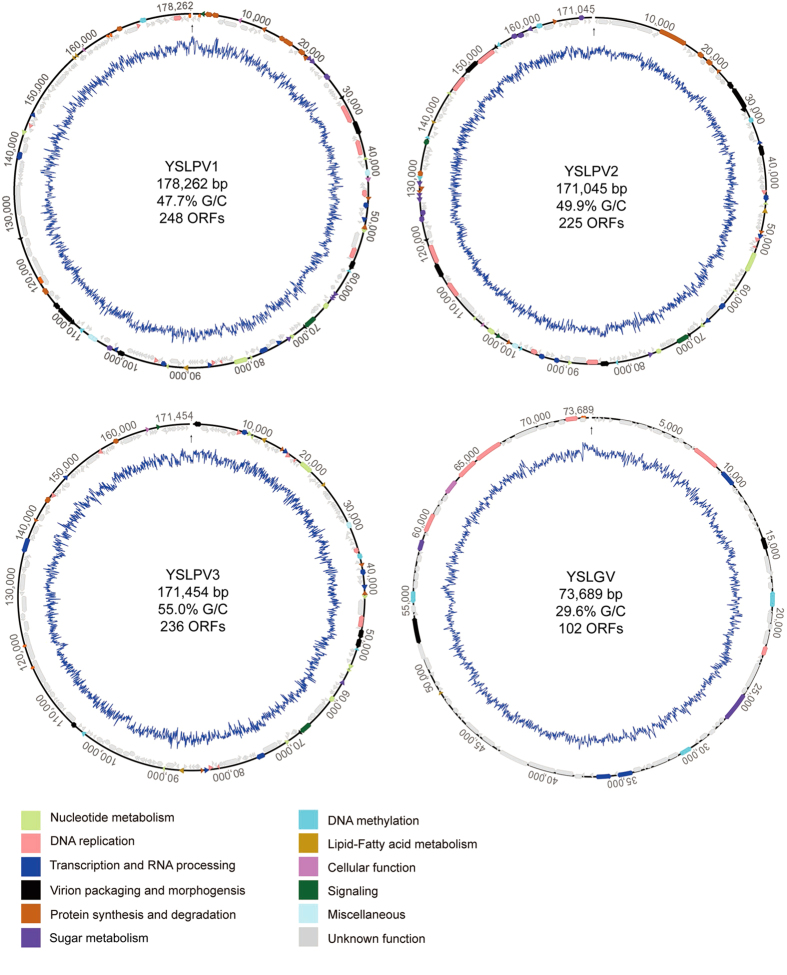
Physical maps of partial genomes of YSLPVs and YSLGV. ORFs are indicated in box arrows and are labeled in different colors that represent different functional categories. Repeats are shown in diamond. Numbers outside the genomes indicate nucleotide positions. Viral names, genomic length and G+C content, and the total number of predicted ORFs are shown in the center of the map. The blue line represents %G+C skew. The ends of each linear genome are indicated with black arrows.

Sequence analysis (see below) indicated that the 178,262, 171,045, and 171,454 bp-long contigs were closely related to phycodnaviruses, while the 73,689 bp-long contig was related to PgV-16 T and OLPVs, which is phylogenetically related with mimiviruses. Accordingly, they were named as Yellowstone Lake phycodnaviruses (YSLPVs 1–3) and Yellowstone Lake giant virus (YSLGV), respectively (Fig. 1). The numbers of predicted ORFs for each virus are shown in Fig. 1. The G/C content of the three YSLPVs (Fig. 1) was similar to that of prasinoviruses (48%), while the G/C content of YSLGV was similar to that of PgV-16 T (32%).

Repeat sequences were found in three partial genomes. Inverted terminal repeats (382 bp) were detected only in YSLPV1 (Fig. 1). Other types of inverted terminal repeats were present on the complete genomic ends of phycodnaviruses, e.g., chloroviruses and phaeoviruses, suggesting that the assembled YSLPV1 genome was nearly complete. In addition, three different types of tandem repeats were also detected in YSLPV1, while one tandem repeat was detected in YSLPV3 and YSLGV (Fig. 1).

**Figure 2:**
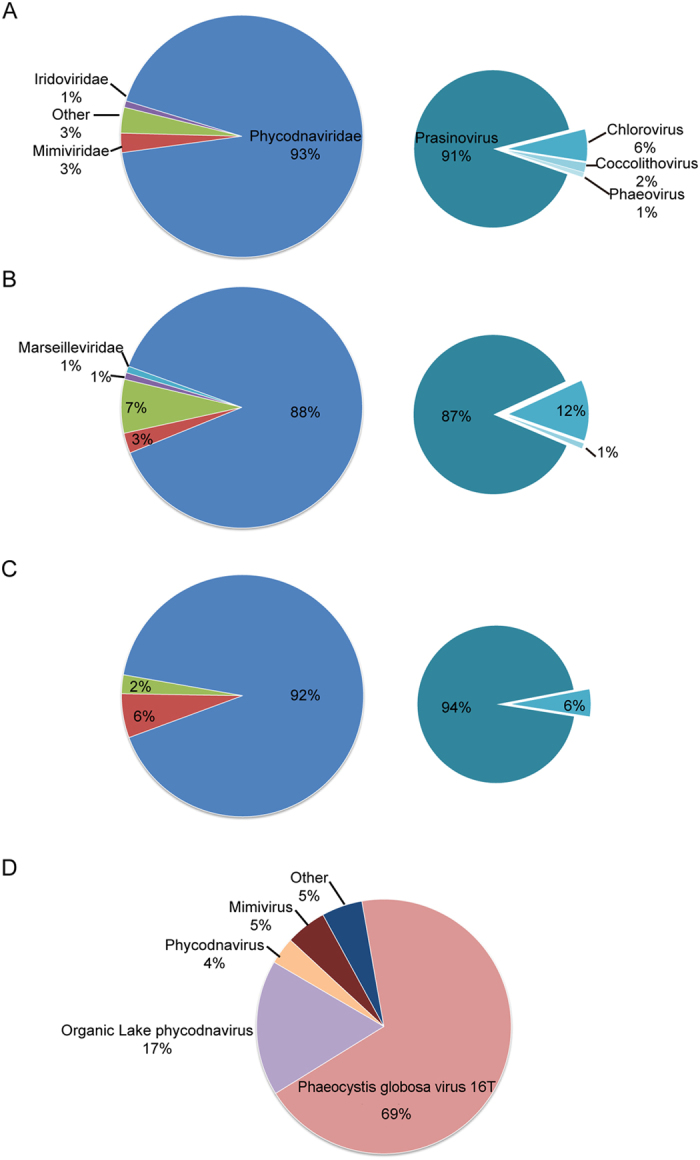
The relative percentages of ORFs, homologous to distinct viral families and to the genera of *Phycodnaviridae* (three pie graphs on the right), in (A) YSLPV1, (B) YSLPV2, (C) YSLPV3 and (D) YSLGV. The same viral family or genus is indicated by a single color. Numbers represent the corresponding percentage of ORFs homologous to that of different viral families or genera.

**Figure 3:**
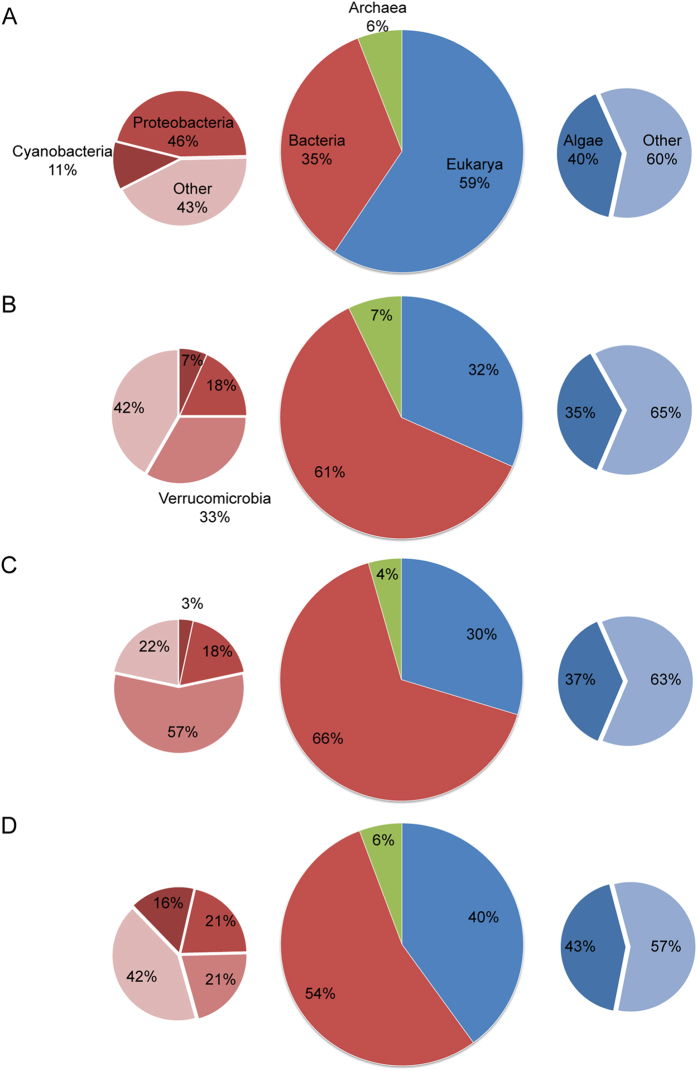
The relative percentage of ORFs, homologous to three domains of life (center pie chart), bacterial phyla (left pie chart), and eukaryotes (right pie chart), in (A) YSLPV1, (B) YSLPV2, (C) YSLPV3 and (D) YSLGV. The same group of organism is indicated by a single color. Numbers represent the corresponding percentages of ORFs homologous to that of different groups of organisms.

BLASTp analysis showed that 33% (YSLPV1), 36% (YSLPV2), 39% (YSLPV3), and 31% (YSLGV) of predicted ORFs were ORFans, which had no detectable homologs in GenBank (Fig. S2). With respect to virus hits, YSLPV1, −2, and −3 shared the most homologs with prasinoviruses (E-value ≤ 0.01, sequence identity 24.0–74.8%), particularly *Ostreococcus* and *Micromonas* viruses. In contrast, YSLGV shared the most homologs with OLPVs (10 hits) and PgV-16 T (40 hits) (Fig. 2). In YSLPV1, 91% of BLAST hits related to *Phycodnaviridae* were from *Prasinovirus* (42 hits to *Micromonas* virus, 38 to *Ostreococcus* virus and 18 to *Bathycoccus* virus). In YSLPV2 and −3, of the *Phycodnaviridae*-related hits, those from *Prasinovirus* accounted for 87% (34 to *Micromonas* virus, 38 to *Ostreococcus* virus and 13 to *Bathycoccus* virus) and 94% (38 to *Micromonas* virus, 48 to *Ostreococcus* virus and 17 to *Bathycoccus* virus), respectively. For non-virus hits, algal genes comprised more than 35% of eukaryotic hits of YSLPV and YSLGV ORFs (Fig. 3). In three YSLPVs, the majority of algae gene homologs were from the four algae classes *Chlorophyceae*, *Trebouxiophyceae*, *Mamiellophyceae*, and *Phaeophyceae*, which were absent in YSLGV. For YSLGV, two algae hits related to *Haptophyceae* and *Cryptophyceae* were observed, one each to *Bangiophyceae* and *Stramenopiles*, respectively. In addition, several ORFs of YSLPV1, −2 and −3 showed similarity to bacterial genes, particularly to *Cyanobacteria* and *Proteobacteria*. YSLPV2 and −3 also had a significant number of hits to *Verrucomicrobia bacterium*: 20 hits of YSLPV2 and 34 of YSLPV3 (Table S2).

**Figure 4:**
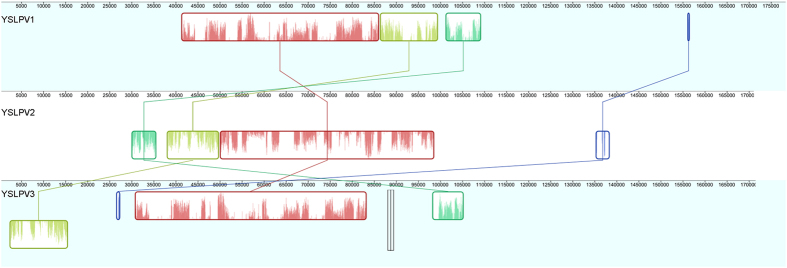
Whole genome alignment of YSLPV1, −2, and −3. Genome inversion and rearrangement of conserved regions are indicated by different colors.

Whole genome alignment of YSLPV1, −2 and −3 showed that three highly conserved regions were shared among the three genomes (Fig. 4), confirming the close phylogenetic relationship as described below. Genome inversion occurred between YSLPV1 and −2, while YSLPV3 experienced genome rearrangement compared to YSLPV1 and −2 (Fig. 4).

### Conserved genes of NCLDVs present in YSLPVs and YSLGV

Based on the results of COG analysis, 39 YSLGV proteins were clustered with OLPG comprising OLPVs and PgV-16 T. Based on conserved domain and functional analyses, 23 NCVOGs were found in YSLPV1, 18 in YSLPV2, 17 in YSLPV3, and 6 in YSLGV (Table 1). Among these, four genes (DNA polymerase sliding clamp (PCNA), VV A32-like packaging ATPase, MCP and VLTF3) were present in all four sequences. Nine genes, present only in YSLPV1, −2 and −3, were identified as YqaJ viral recombinase family protein, ribonucleotide reductase large subunit (RNR1), ribonucleotide reductase small subunit (RNR2), mRNA-capping enzyme, transcription initiation factor IIB, TATA-box binding protein, DNA helicase of superfamily II, thioredoxin fold protein and serine/threonine protein kinase. In addition, YSLPV1 and −2 shared 18 NCVOGs (Table 1). However, a number of core genes in prasinoviruses were absent in YSLPVs and YSLGV. For example, PolB was not detected in YSLGV and YSLPV3, and Rnase H was absent in YSLPVs 1–3. These omissions were likely a result of incomplete genomic sequences or due to high divergence of the corresponding proteins.

**Figure 5:**
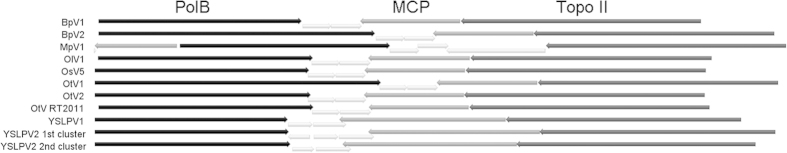
Conserved gene cluster of PolB, MCP, and Topo II in the members of *Prasinovirus* and YSLPV1, −2 and −3.

Gene duplication, which is commonly present in giant dsDNA viruses, was also found in YSLPVs and YSLGV. For example, YSLPV2 encodes two PolB paralogs (ORF143 and ORF195), which shared 69% amino acid identity and were most similar to their homologs in prasinoviruses (Table S2); YSLGV contained two copies of topoisomerase gene similar to PgV-16 T^25^. In addition, a conserved gene cluster, comprising three core genes of PolB, MCP and Topo II, was observed in YSLPV1 and −2 as well as in other prasinovirus genomes (Fig. 5). Interestingly, this gene cluster was duplicated in YSLPV2, which has not been identified in other NCLDV viruses. This duplication is unlikely to be due to the presence of sequence assembly artifacts in the duplicated gene cluster, since their corresponding scaffold sequences were reliable with good quality and high coverage.

### Other key functional genes

Genes involved in DNA replication, nucleotide metabolism, transcription, and virion packaging were frequently found in YSLPVs and YSLGV (Table 1). Genes involved in sugar metabolism, such as glycosyltransferase, were detected in YSLPVs and YSLGV (Table S2), and these genes are also common in NCLDVs. Other functional genes that are involved in lipid metabolism, such as patatin-like phospholipase, were also identified in YSLPVs and YSLGV. The detailed information is shown in Table 1 and Table S2. It is worth noting that YSLPVs encoded several genes, including deoxynucleoside kinase, HNH nuclease, SKP1 protein and ornithine: arginine decarboxylase, were uniquely found in chloroviruses^7^.

### Phylogenetic analysis

**Figure 6:**
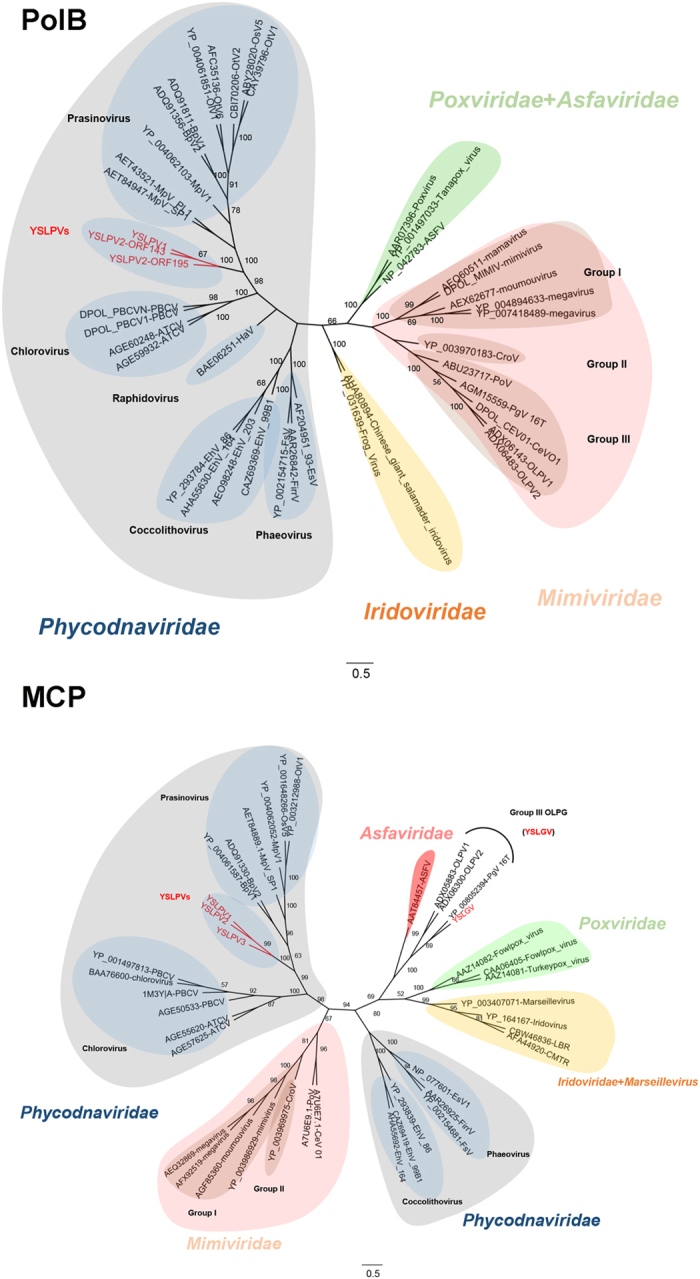
Unrooted phylogenetic trees reconstructed using amino acid sequences of NCLDV conserved genes of PolB and MCP that are present in YSLPVs, YSLGV and selected other giant virus families. Viral families, genera of *Phycodnaviridae* and three groups of *Mimiviridae* are shaded in different colors. The *Mimiviridae* family has currently only two ICTV-approved members, *Acanthamoeba polyphaga mimivirus* and *Cafeteria roenbergensis virus*. The subgroupings into Group I-III are arbitrary, non-formal classifications. Bootstrap values (100 iterations) are indicated on the branching of the tree.

Five conserved proteins of NCLDVs, DNA polymerase B, MCP, VLTF3, topoisomerase II and VV A32-like packaging ATPase, were used to construct phylogenetic trees. According to the tree topology (Fig. 6; Fig. S3), YSLPV1, −2 and −3 formed a distinct monophyletic group, which represented a sister lineage to and shared a common ancestor with prasinoviruses. YSLGV clustered with the OLPG group, including PgV-16 T and OLPVs. Taken together, both sequence and phylogenetic analyses indicated that YSLPV1, −2, and −3 appeared to be a novel algal virus lineage most closely related to *Prasinovirus* and that YSLGV seemed to be a novel member of OLPG.

### Relevance to virophages

**Figure 7:**
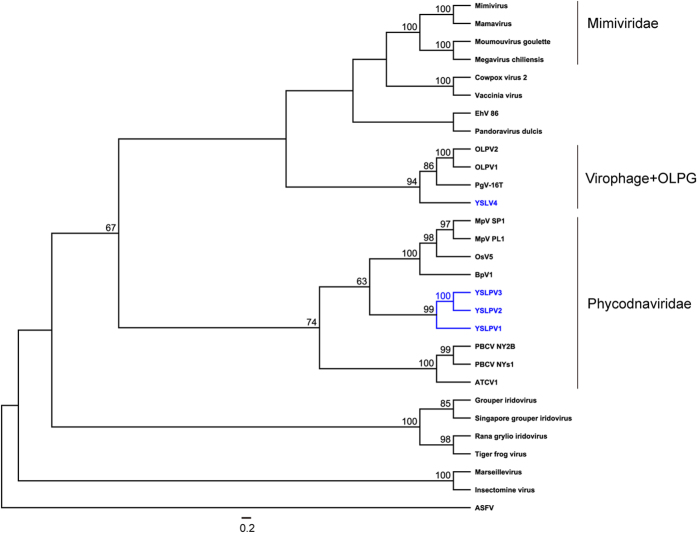
Phylogenetic tree reconstructed using amino acid sequences of RNR2 of YSLPVs, YSLV4-ORF04, and selected other giant viral families. *Mimiviridae*, OLPG group, and *Phycodnaviridae* are labeled with lines. YSLPVs and YSLV4-ORF04 are indicated in blue. African swine fever virus (ASFV) was used to root the tree. Bootstrap values (100 iterations) are shown on the branching of the tree. The accession numbers of the sequences used are as follows: ASFV (NP_042738), ATCV1 (YP_001426516), BpV1 (YP_004061574), Cowpox virus 2 (ADZ29378), EhV 86 (YP_293780), Grouper iridovirus (AAV91049), Insectomime virus (AHA46032), Mamavirus (AEQ60501), Marseillevirus (YP_003407004), Megavirus chiliensis (YP_004894645), Mimivirus (YP_003986815 ), Moumouvirus goulette (AGF85216), MpV PL1 (AET43587), MpV SP1 (AET84875), OLPV1 (ADX05815), OLPV2 (ADX06189), OsV5 (YP_001648251), Pandoravirus dulcis (YP_008319229), PBCV NY2B (AGE54331), PBCV NYs1 (AGE55017), PgV-16 T (YP_008052426), Rana grylioirido virus 9506 (AAS67856), Singapore grouper iridovirus (YP_164142), Tiger frog virus (AAL77807), and Vaccinia virus (AEY74987).

A total of nine non-redundant homologous counterparts shared between YSLPVs/YSLGV and YSLVs were determined based on BLAST searches of a local database containing all YSLV ORFs (n = 193), using YSLPVs/YSLGV ORFs as query sequences (Table 2). YSLV4 ORF04 shared the highest similarity with YSLPV1, −2 and −3 ORFs (>50%, E-value < e-90), which were homologous to RNR2, a conserved gene in NCLDVs. Phylogenetic analysis of viral RNR2 protein sequences (Fig. 7) showed that YSLV4 RNR2 was clustered with that of OLPG, and the RNR2 homologs of YSLPVs were grouped into a single clade that is closely related to prasinovirues. Although RNR2 was not detected in YSLGV, it does not exclude the possibility of its presence since the YSLGV genomic sequence was incomplete and YSLGV appeared to be a new member of OLPG. OLV ORF2 is a gene with unknown function that is conserved in YSLV 1–4 and −6 and is present in both YSLPV1–3 and YSLGV (Table 2).

### Host defense gene in YSLPVs

**Figure 8:**
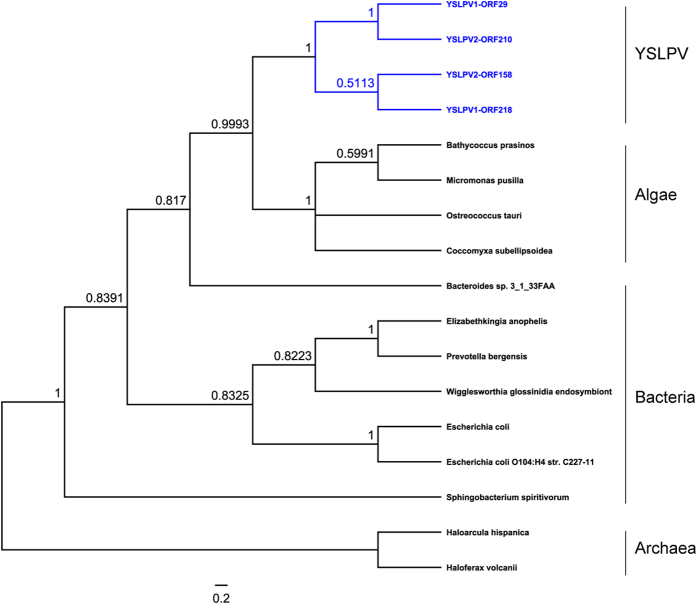
Rooted Bayesian phylogenetic tree reconstructed using amino acid sequences of emrE in YSLPV 1–2 and selected bacterial, eukaryotic, and archaeal species. YSLPVs, Algae, Bacteria and Archaea groups are marked with lines. YSLPVs are shown in blue. Bayesian posterior probabilities are indicated on the branching of the tree. The tree is rooted using Archaea emrE. The accession numbers of the sequences used are as follows: *Haloarcula hispanica* (WP_014039315), *Haloferax* (WP_004045282), *Sphingobacterium spiritivorum* (WP_002995628), *Escherichia coli* (WP_032285312), *Escherichia coli* O104:H4 str. C227-11 (WP_001304280), *Wigglesworthia glossinidia* endosymbiont (WP_011070390), *Elizabethkingia anophelis* (CDN79378), *Prevotella bergensis* (WP_007174771), *Bacteroides* sp.3_1_33FAA (WP_008654963), *Ostreococcus tauri* (XP_003082847), *Coccomyxa subellipsoidea* c-169 (XP_005651632), *Bathycoccus prasinos* (XP_007513508), and *Micromonas pusilla* CCMP1545 (XP_003063730).

YSLPV2 ORF158 and -ORF210 contain a multidrug resistance domain. YSLPV1 ORF29 showed sequence similarity with the quaternary ammonium compound resistant protein of *Natrinema gari* (29.8%, E value 0.035), while YSLPV1 ORF218 shared 33.3% sequence similarity with the multidrug resistant protein MdtJ of *Yersinia pseudotuberculosis* IP 31758, an enterobacteria species, with an E value of 5.88e-04 (Table S2). Based on HHpred analysis, YSLPV1 ORF29, -ORF218, YSLPV2 ORF158, and -ORF210 were homologous to a multidrug transporter emrE protein (Table S3) that is approximately 110 amino acids in size and exports positively charged hydrophobic drugs in exchange for protons, thereby conferring bacterial resistance to toxic compounds^26^. Phylogenetic analysis revealed that YSLPV emrE shares a common ancestor with eukaryotic algae emrE that possibly originated from bacteria (Fig. 8).

## Discussion

In this study, partial genomic sequences of four novel giant viruses were obtained from YSL metagenomic data sets, from which seven virophages had previously been discovered^15^,^16^. The YSLPV1 genome appears nearly complete as it contains a pair of inverted repeats flanking both ends of the genome and a more complete set of NCVOGs than the other three genomes. YSLGV is far from complete as it is the shortest assembled sequence, approximately 73 kb in length, which does not meet the criteria for genome length of giant viruses (>100 kb). Despite the lack of several core genes in the partial genome, including PolB, RNR1, RNR2 and DNA ligase, which are fundamental to DNA replication and nucleotide metabolism, YSLGV contains capsid protein, Topoisomerase II, Packaging ATPase, PCNA, and late transcription factor VLTF3 that all clustered with those of OLPG. Moreover, the presence of the RNA polymerase gene distinguishes YSLGV from YSLPVs and prasinoviruses, since most phycodnaviruses do not contain RNA polymerase genes^27^,^28^.

Based on homolog and phylogenetic analyses, YSLPV 1–3 belong to the phycodnaviruses, and YSLGV is grouped to the OLPG clade affiliated with mimiviruses. YSLPVs represent a novel viral lineage in *Phycodnaviridae* and are more closely related to prasinoviruses that infect marine algae than to chloroviruses that infect freshwater algae. Since Yellowstone Lake is a freshwater ecosystem containing hundreds of hydrothermal vents^29^, YSLPVs, unlike their marine algae-infecting relatives of *Prasinovirus*, have the potential to infect freshwater algae similarly to chloroviruses. In addition, YSLGV appears to be a novel member of Group III, the extended family of *Mimiviridae*, whose eukaryotic hosts are thought to be algae, not protozoa. The Yellowstone Lake therefore contains a diverse set of giant algal viruses, which await further study.

Virophages were found to be associated with mimiviruses whose genome sizes are typically larger than 1 megabase and replicate in cytoplasmic viral factory^14^. Sputnik^30^ and Mavirus^31^ are parasites of mimivirus group I and group II (*Cafeteria roenbergensis* virus, CroV), respectively. OLV^18^ was thought to be associated with tentative group III mimiviruses (OLPVs). The recently isolated virophage Zamilon is capable of infecting lineage C (Group I) members of *Mimiviridae*^32^. A virophage-like sequence, termed PgVV, was obtained during assembly of the third largest genome of marine virus of PgV-16 T (459,984 bp in length) using metagenomic data^25^, although no virophage particles were observed. The PgVV genomic features are unique: 1-kb telomeric-like repeats flanking the genome, 16 coding regions that all transcribe from the same strand, and a putative jelly-roll capsid protein (PgVV ORF12) that bears little similarity to that of other virophages^33^. The discovery of pro-virophage PgVV is the first report of a virophage associated with a mimivirus-like giant algal virus, and it also implies that virophages are capable of infecting giant viruses of comparatively smaller genome sizes^34^. Thus far, however, no evidence has been provided to indicate that virophages are able to infect giant algal viruses of phycodnaviruses.

**Figure 9:**
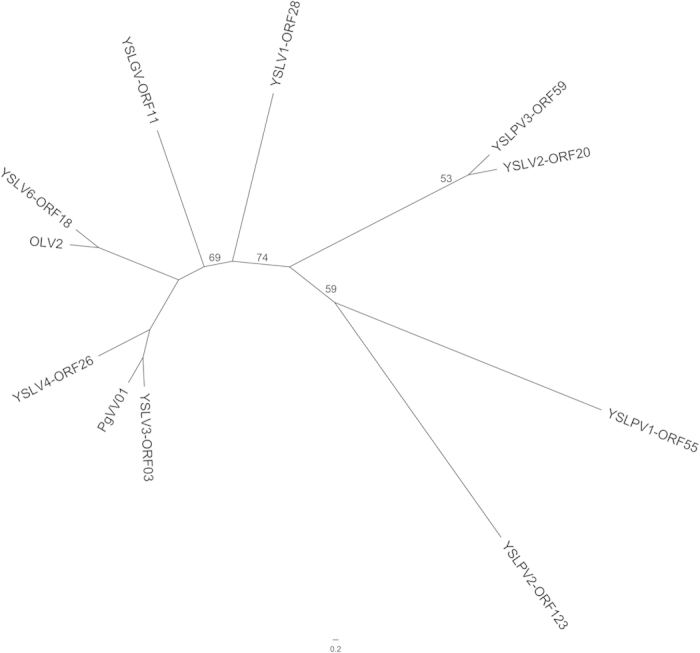
Unrooted phylogenetic tree reconstructed using amino acid sequences of OLV2 and its homolog counterparts in YSLPV1-3, YSLGV, YSLV1−4 and −6, and PgVV. Bootstrap values (>50, 100 iterations) are indicated on the branching of the tree.

It has been shown that virophages and their associated giant viral hosts share homologous genes (Table S4). In this study, comparative genomic analysis revealed that YSLV 1–4 and −6 exhibit genetic links with YSLPVs and YSLGV. For example, nine non-redundant homologous genes are shared between YSLVs and YSLPVs/YSLGV (Table 2), and this number may increase when the complete genomes of YSLPVs and YSLGV are available. The homolog counterparts of function-unknown OLV ORF2, which is conserved in YSLV 1–4 and −6, OLV and PgVV (ORF01) (Table S5), were present in YSLGV and YSLPV 1–3, and phylogenetic analysis of the OLV ORF2 homolog grouped YSLV2 with YSLPVs, suggesting that gene transfer may have occurred between them (Fig. 9). Taken together, although it is too preliminary to conclude a potential association between virophages and phycodnaviruses, the study of giant algal viruses may be informative in the search for novel virophages.

Surprisingly, homologs of multidrug transport protein emrE were identified in YSLPV1 and −2. To our knowledge, emrE has not previously been reported in phycodnaviruses or in any other known virus. It is likely that YSLPVs derived emrE genes from their potential algal hosts that contained emrE genes obtained from their symbiotic bacteria through horizontal gene transfer (Fig. 8). The function of emrE and its ecological and evolutionary fitness to YSLPVs remain to be explored.

In conclusion, four algal viral partial genomic sequences were discovered from the Yellowstone Lake metagenomics dataset. The corresponding novel viruses of YSLPV 1–3 and YSLGV are related to members of the *Phycodnaviridae* and the OLPG clade related to *Mimiviridae*, respectively, indicating the diversity of algal virus species in YSL ecosystem. Genetic links between YSLPVs/YSLGV and YSLVs were observed, while their potential associations await future study. Yellowstone Lake is a hotspot for studying the diversity of algal giant viruses and virophages.

## Additional Information

**How to cite this article**: Zhang, W. *et al.* Four novel algal virus genomes discovered from Yellowstone Lake metagenomes. *Sci. Rep.* **5**, 15131; doi: 10.1038/srep15131 (2015).

## Supplementary Material

Supplementary Information

## Figures and Tables

**Table 1:** Key functional genes in YSLPVs and YSLGV.

Gene function	YSLPV1	YSLPV2	YSLPV3	YSLGV
DNA replication				
**Family B DNA polymerase**	46	143, 195		
**PCNA**	131	44	12, 137	101
**RuvC like Holliday junction resolvase/Pox A22**	185		192	
**ATP dependent DNA ligase**	245			
**Topoisomerase II**	42	148, 199		93, 94
**YqaJ viral recombinase**	114	57	27, 123, 210	
Replication factor C	56	122	62	^**†**^35, 88
SF3 Helicase	66	112	74	
Endonuclease (similar to *Chlorovirus*)	235			
^**+**^NTPase/helicase	111	60	29, 120	
Nucleotide metabolism				
**dUTPase**	48	139, 193		
**Ribonucleotide reductase large subunit**	105	63	32	
**Ribonucleotide reductase small subunit**	82	94	92	
**Thymidylate synthase**	183		83	
^*^Deoxynucleoside kinase	92	82	105	
CYTH-like_mRNA_RTPase	64	114	72	
^*^HNH nuclease	103	72, 133	14, 84	
PDDEXK	127	46	15, 135	
Transcription and RNA processing				
**mRNA-capping enzyme**	61	117	70	
**DNA-directed RNA polymerase II subunit D**				53
**Nudix hydrolase**	142?	33		
**Transcription initiation factor IIB**	58	120	67	
^**+**^**TATA-box binding protein**	96	80	106	
**Poxvirus late transcription factor VLTF3**	128	45	13, 136	17
**Transcription factor S-II**	190		198	
**DNA helicase of superfamily II (Dead-like?)**	99	78	108	
**VV A18 helicase**	178		181	
RNA ligase				55
RNase III	115	56	26, 124	
Virion packaging and morphogenesis				
**Thiol oxidoreductase/Evr1_Alr**	39, 166	150, 201		
**Thioredoxin fold protein**	87?	87?	100	
**Capsid protein**	43, 68, 141, 154, 155	22, 23, 34, 110, 147, 198	01, 76, 77	77
**VV A32-like packaging ATPase**	153	24	157	25
Protein synthesis and degradation				
Ubiquitin hydrolase-like cystein peptidase	63	115	71	
DNaJ	232		217	
^**+**^Aminotransferase family protein	24			
^**+**^2OG-Fe(II) oxygenase	05, 160	13, 15		
^**+**^NAD-dependant epimerasedehydratase (NADB domain)	20, 25	167		
Swib domain protein		55	25, 125	
^**+**^Acetolactate synthase (2-2-1-6) (TPP)	21			
transketolase domain-containing protein (TPP)		163, 164		
^**+**^putative ATP-dependent protease proteolytic subunit	57	128	65	
^**+**^Aspartyl/Asparaginyl beta-hydrolase			186	
Prolyl 4-hydroxylase	06, 16	17, 217		
^**+**^N-myristoyl transferase			191	
Sugar metabolism				
GDP-D-mannose dehydratase epimerase (GMD)	32	156, 209		
^**+**^Glycosyltransferase	26, 27, 77, 78, 79, 93, 144	161,162, 212	87	40, 86
DNA methylation				
Adenine DNA methyltransferase	69, 150, 236	27, 107, 125, 180	63, 78, 154	30, 47
Methyltransferase FkbM		166		
D12 class N6 adenine-specific DNA methyltransferase				80
Lipid – Fatty acid metabolism				
Patatin-like phospholipase	123	48	18, 131	71
Acyl-CoA N-acyltransferase	211, 212	184	39	
Cellular function				
*SKP1 protein	217	135		
Cell division protein (CDC48_2)	10		224	
von Willebrand factor A	52			
Alanine racemase				92
Signaling				
PhoH	04	131	228	
**Serine/Threonine protein kinase**	86	88	99	
Miscellaneous				
*Ornithine:Arginine decarboxylase	51	127	56	
Fibronectin binding protein	147			

^**+**^indicates genes only found in prasinoviruses.

*indicates genes only found in chloroviruses.

NCVOGs are indicated in bolded.

ORF numbers are indicated in the cell.

^†^35 and 88 in YSLGV column reflect small subunit of replication factor C and large subunit of replication factor C, respectively.

**Table 2:**
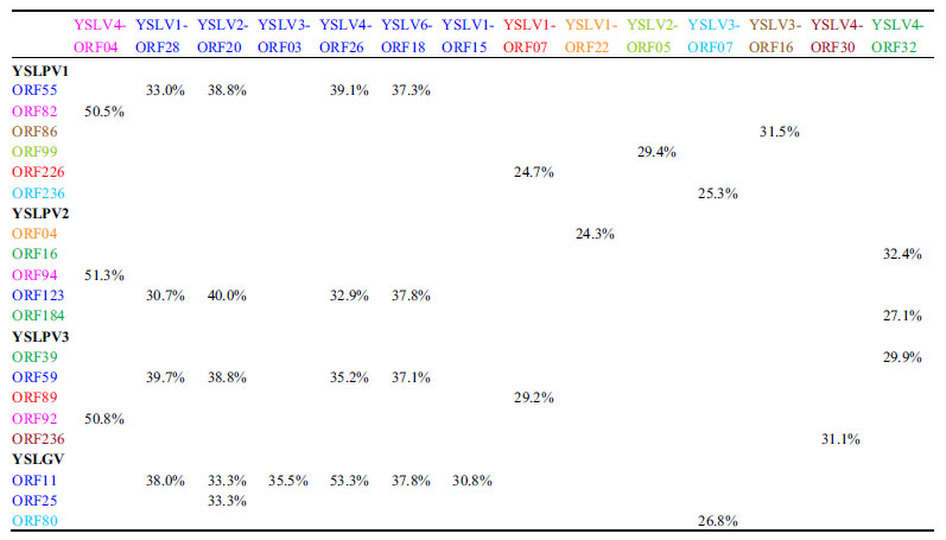
Homologous genes shared between YSLPVs/YSLGV and YSLVs.

Percentages indicate shared amino acid identity. Homologous genes are represented by the same color.
