# Supplementary material for: Four novel algal virus genomes discovered from Yellowstone Lake metagenomes
Source: Sci Rep. 2015 Oct 13;5:15131. doi: 10.1038/srep15131 (PMC4602308; doi:10.1038/srep15131)
Supplement: Supplementary Information [file srep15131-s1.pdf]

Supplementary data

Four novel algal virus genomes discovered from Yellowstone Lake metagenomes

Weijia Zhang[a], Jinglie Zhou[a,b], Taigang Liu[c], Yongxin Yu[a,d], Yingjie Pan[a,d,e], Shuling Yan[a,f], Yongjie Wang[a,d,e]*

[a] College of Food Science and Technology, Shanghai Ocean University, Shanghai, China;

[b] Department of Biological Sciences, Auburn University, Auburn, AL, USA;

[c] College of Information Technology, Shanghai Ocean University, Shanghai, China;

[d] Laboratory of Quality and Safety Risk Assessment for Aquatic Products on Storage & Preservation, Ministry of Agriculture, Shanghai, China;

[e] Shanghai Engineering Research Center of Aquatic-Product Processing & Preservation, Shanghai, China;

[f] Institute of Biochemistry and Molecular Cell Biology, University of Göttingen, Göttingen, Germany;

Table S1 Data on metagenomic assemblies of four YSL sequences

Name	No. of reads recruited to each genome	No. of identical sites	Pairwise identity (%)	Genome Coverage			Size of dataset (Gb)
				Mean	Minimum	Maximum	
YSLPV1	17,474	163,529	98.5	32.0	1	107	11.1
YSLPV2	14,874	157,105	97.5	32.8	1	172	11.1
YSLPV3	13,946	157,872	98.4	28.8	1	79	11.1
YSLGV	3,018	69,407	98.0	13.8	1	46	11.1

Table S2 ORFs and their homologs predicted in YSLPVs and YSLGV

ID	Position		Length		BlastP hit in GenBank nr and/or viruses data set						NCBI conserved domain (ID, E-value, alignment length in aa, alignment start - end position)	Interproscan matches (ID, E-value, alignment start - end position)
	start	end	nt	aa	ORF, protein encoded, or mass	Species	Accession	Aa identity	E Value	Alignment length in aa (Start-end position)		
YSLPV1												
1	29	472	444	147	ring finger protein, putative	Ricinus communis	XP_002522531	42.40%	2.69E-11	63(1-63)	RING superfamily(cd00162, 7.94e-12,44,2-45)	zf-RING_2(PF13639 ,7.5E-16 2-43)
											Zinc finger, C3HC4 type(pfam13923,2.89e-09,42,2-43)	
2	435	641	207	68								
3	870	1,412	543	180								
4	1,524	2,144	621	206	hypothetical protein MpV1_231c	Micromonas sp. RCC1109 virus MpV1	YP_004062114	51.20%	3.91E-67	201(5-205)	PhoH(pfam02562,2.92e-85,205,1-205)	PhoH(PF02562,PhoH 1.3E-69 2-205)
					PhoH-like protein	Synechococcus sp. PCC 7002	YP_001734198	46.30%	6.72E-57	204(2-205)		P-loop containing nucleoside triphosphate hydrolases(SSF52540,1.3E-28 2-181)
5	2,197	3,297	1,101	366	oxidoreductase domain protein	Caenispirillum salinarum AK4	ZP_18917440	45.00%	2.00E-31	155(212-366)	2OG-FeII_Oxy_3 Superfamily(cl17304,8.09e-15 ,83,279-361)	FE2OG_OXY(PS51471,10.45,268-362)
					2OG-Fe(II) oxygenase	Cyanophage Syn30	YP_007877817	44.00%	2.63E-26	137(229-365)		Prolyl 4-hydroxylase alpha subunit homologue(SM00702,4.3E-5,186-361)
												PKHD-TYPE HYDROXYLASE(PTHR30496,2.1E-6,188-361)
												Clavaminate synthase-like(SSF51197 ,5.5E-6,188-358)
6	3,302	4,393	1,092	363	2OG-Fe(II) oxygenase	Synechococcus phage S-SM1	YP_004322947	41.20%	1.37E-31	179(10-188)	2OG-FeII_Oxy_3 Superfamily(cl17304,2.50e-07,139,20-158;5.75e-04,126,211-336)	2OG-FeII_Oxy_3(PF13640,1.6E-7,112-187;7.4E-9,269-356)
					prolyl 4-hydroxylase	Candidatus	YP_827606	41.70%	3.20E-11	94(266-359)		FE2OG_OXY(PS51471,9.635,106-190)

ID	Position		Length		BlastP hit in GenBank nr and/or viruses data set						NCBI conserved domain (ID, E-value, alignment length in aa, alignment start - end position)	Interproscan matches (ID, E-value, alignment start - end position)
	start	end	nt	aa	ORF, protein encoded, or mass	Species	Accession	Aa identity	E Value	Alignment length in aa (Start-end position)		
					subunit alpha	Solibacter usitatus Ellin6076						
												Prolyl 4-hydroxylase alpha subunit homologue(SM00702,1.1E-4,10-189)
7	4,421	5,332	912	303								
					hypothetical protein OsV5_030f	Ostreococcus virus	YP_001648107	60.00%	1.11E-06	35(5-39)	PhoH superfamily(pfam02562,8.51e-13,37,1-37)	PhoH(PF02562,3.3E-9,2-39)
8	5,314	5,481	168	55								
					phosphate starvation-inducible protein ATPase PhoH	Candidatus Chloracidobacterium thermophilum B	YP_004861930	64.90%	3.89E-06	37(1-37)		P-loop containing nucleoside triphosphate hydrolases(SSF52540,4.2E-6,2-40)
9	5,514	7,604	2,091	696								
					vesicular-fusion protein nsf, putative	Ricinus communis	XP_002519069	33.00%	8.43E-15	142(15-156)	CDC48_2 (smart01072,7.81e-05,49,112-160)	ADC-like(SSF50692,6.9E-13,1-78)
10	7,637	8,140	504	167								
												VESICULAR-FUSION PROTEIN NSF(PTHR23078,4.9E-11,1-146)
					hypothetical protein BpV1_108c	Bathycoccus sp. RCC1105 virus BpV1	YP_004061538	37.00%	1.70E-91	434(2-435)		ARIADNE RING ZINC FINGER(PTHR11685,4.4E-7,224-264)
11	8,205	9,545	1,341	446								
					hypothetical protein	Trichomonas vaginalis G3	XP_001301335	32.90%	1.16E-12	131(156-286)		RING/U-box(SSF57850,7.0E-9,228-285)
12	9,665	10,267	603	200								
					hypothetical protein BpV2_179	Bathycoccus sp. RCC1105 virus BpV2	ADQ91346	41.30%	1.86E-16	109(37-145)		signal-peptide(SignalP-NN(euk),-1.0,1-57)
13	10,248	10,703	456	151								
												transmembrane_regions(tmhmm,-1.0,35-53;<br>-1.0,59-77;-1.0,119-137)
14	10,744	11,250	507	168								transmembrane_regions(tmhmm,-1.0,66-88)

ID	Position		Length		BlastP hit in GenBank nr and/or viruses data set						NCBI conserved domain (ID, E-value, alignment length in aa, alignment start - end position)	Interproscan matches (ID, E-value, alignment start - end position)
	start	end	nt	aa	ORF, protein encoded, or mass	Species	Accession	Aa identity	E Value	Alignment length in aa (Start-end position)		
15	11,197	11,604	408	135								signal-peptide(SignalP-NN(euk),-1.0,1-35)
												transmembrane_regions(tmhmm,-1.0,15-35)
16	11,612	12,286	675	224	putative prolyl 4-hydroxylase	Acanthamoeba polyphaga mimivirus	YP_003987108	43.00%	1.73E-52	195(28-222)	P4Hc(smart00702,1.51e-28,172,47-218)	FE2OG_OXY(PS51471,10.599,108-219)
					oxidoreductase, 2OG-Fe(II) oxygenase family protein	Acidithiobacillus sp. GGI-221	ZP_11561250	39.50%	6.00E-33	180(44-223)	2OG-FeII_Oxy_3(cl17304,1.51e-28,172,47-218)	Prolyl 4-hydroxylase alpha subunit homologue(SM00702,5.4E-31,38-218)
												PROLYL 4-HYDROXYLASE ALPHA SUBUNIT(PTHR10869,1.9E-37,36-222)
												signal-peptide(SignalP-NN(euk),-1.0,1-26)
												transmembrane_regions(tmhmm,-1.0,4-22)
17	12,286	13,689	1,404	467								
18	13,732	14,082	351	116	hypothetical protein BpV2_001c	Bathycoccus sp. RCC1105 virus BpV2	ADQ91168	46.90%	1.28E-27	111(1-111)	rI.-1(PHA02604,4.27e-19,111,5-115)	
19	14,103	14,738	636	211	glycosyl transferase group 1	Fischerella sp. JSC-11	ZP_08986113	28.80%	3.94E-13	197(5-201)	Methyltransf_24(pfam13578, 2.42e-21,119,47-165)	Methyltransf_24(PF13578,6.8E-17,47-165)
					Pas34	Actinoplanes phage phiAsp2	YP_024820	26.70%	3.86E-05	148(17-164)		S-adenosyl-L-methionine-dependent methyltransferases(SSF53335,2.2E-8,45-165)
20	14,679	15,629	951	316	NAD dependent epimerase/dehydratase family protein	Trichomonas vaginalis G3	XP_001297395	47.50%	6.21E-99	312(3-314)	NADB_Rossmann Superfamily(cl09931,2.26e-151,307,1-307)	Epimerase(PF01370,5.6E-64,3-240)
					GDP-L-fucose synthase 2	Acanthocystis turfacea Chlorella	AGE59831	46.60%	1.75E-95	305(3-307)	RfbB(COG1088,3.33e-131,311,1-311)	DTDP-GLUCOSE 4,6-DEHYDRATASE(PTHR10366:SF41,3.8E

ID	Position		Length		BlastP hit in GenBank nr and/or viruses data set						NCBI conserved domain (ID, E-value, alignment length in aa, alignment start - end position)	Interproscan matches (ID, E-value, alignment start - end position)
	start	end	nt	aa	ORF, protein encoded, or mass	Species	Accession	Aa identity	E Value	Alignment length in aa (Start-end position)		
						virus TN603.4.2						-141,1-316)
												NAD(P)-binding Rossmann-fold domains(SSF51735,4.4E-86 ,1-313)
					thiamine pyrophosphate enzyme TPP binding domain protein	Clostridium botulinum B str. Eklund 17B	YP_001886523	37.80%	1.84E-123	554(1-554)	TPP_PYR_POX_like(cd07035,2.79e-45,156,5-160)	TPP_enzyme_C(PF02775,1.7E-30,391-504)
21	15,690	17,357	1,668	555							TPP_enzyme_M(pfam00205,2.96e-36,136,193-328)	TPP_enzyme_M(PF00205,1.3E-29,193-328)
											TPP_enzymes(cd00568,3.96e-44,138,367-504)	TPP_enzyme_N(PF02776,8.9E-47,1-174)
											IlvB(COG0028,4.80e-137,533,1-533)	THIAMINE PYROPHOSPHATE ENZYMES(PTHR18968,2.8E-117,1-505)
					hypothetical protein ALOHA_HF4000007I05ctg1g9	uncultured marine microorganism HF4000_007I05	ABZ06236	35.70%	2.40E-29	187(4-190)	CDO_I(pfam05995,5.83e-03,88,12-99)	CDO_I(PF05995,1.9E-5,14-106)
22	17,381	18,031	651	216								
												RmlC-like cupins(SSF51182,8.9E-14,8-130)
					putative DJ-1/PfpI family protein	uncultured marine microorganism HF4000_007I05	ABZ06235	48.00%	6.63E-46	170(3-172)	GAT_1 Superfamily(cl00020,4.96e-52,173,2-174)	PEPTIDASE_C56_PFPI(PS51276,46.123,1-176)
23	17,983	18,552	570	189								
											PfpI(TIGR01382,2.02e-37 ,172,2-173)	Class I glutamine amidotransferase-like(SSF52317,3.2E-39,1-175)
					hypothetical protein OtV6_039	Ostreococcus tauri virus RT-2011	AFC34947	74.80%	0	377(20-396)	AAT_I Superfamily(cl00321,4.17e-79,353,35-387)	Predicted pyridoxal dependent aminotransferase, DegT/DnrJ/EryC1/StrS types(PIRSF000390,6.2E-71,1-394)
24	18,564	19,763	1200	399								
					predicted protein, partial	Phaeodactylum tricornutum CCAP	XP_002180908	54.70%	2.97E-126	355(35-389)	PRK15407(PRK15407,6.32e-94,364,31-394)	PLP-dependent transferases(SSF53383,1.6E-76,20-393)

ID	Position		Length		BlastP hit in GenBank nr and/or viruses data set						NCBI conserved domain (ID, E-value, alignment length in aa, alignment start - end position)	Interproscan matches (ID, E-value, alignment start - end position)
	start	end	nt	aa	ORF, protein encoded, or mass	Species	Accession	Aa identity	E Value	Alignment length in aa (Start-end position)		
						1055/1						
25	19,645	20,436	792	263	hypothetical protein OtV6_036c	Ostreococcus tauri virus RT-2011	AFC34944	41.20%	2.31E-39	208(2-209)	NADB_Rossmann Superfamily(cl09931,4.65e-09,93,7-99)	ASPARTATE DECARBOXYLASE ASDA(PTHR11751:SF119,1.0E-35,33-362) Epimerase(PF01370,9.5E-10,25-177)
					hypothetical protein ACD_20C00109G0012	uncultured bacterium	EKE04057	31.50%	4.67E-22	219(1-219)		NAD(P)-binding Rossmann-fold domains(SSF51735,8.8E-14,1-202)
26	20,461	21,159	699	232	hypothetical protein MpV1_031c	Micromonas sp. RCC1109 virus MpV1	YP_004061914	34.50%	5.83E-39	229(3-231)	Glyco_transf_34 Superfamily(cl05288,5.69e-11,185,23-207)	Glyco_transf_34(PF05637,1.6E-6,22-93;2.5E-7,100-207)
					hypothetical protein COCSUDRAFT_54970	Coccomyxa subellipsoidea C-169	EIE18426	25.00%	2.47E-10	229(4-232)		
27	21,166	21,936	771	256	putative N-acetylglucosaminyltransferase	Flavobacteria bacterium BAL38	ZP_01733049	34.60%	1.82E-31	228(1-228)	Glyco_transf_17(pfam04724,1.03e-20,198,3-200)	
					hypothetical protein NY2A_B618R	Paramecium bursaria Chlorella virus NY2A	YP_001497814	31.00%	1.75E-23	199(2-200)		Glyco_transf_17(PF04724,1.0E-33,3-229)
28	21,821	22,168	348	115	hypothetical protein OLOG_00294	Ostreococcus lucimarinus virus OlV4	AET84745	44.30%	1.20E-06	70(7-76)		
29	22,191	22,517	327	108	quaternary ammonium compound-resistance protein	Natrinema gari JCM 14663	ZP_21532505	29.80%	3.54E-02	101(2-102)		signal-peptide(SignalP-NN(euk),-1.0,1-52)

ID	Position		Length		BlastP hit in GenBank nr and/or viruses data set						NCBI conserved domain (ID, E-value, alignment length in aa, alignment start - end position)	Interproscan matches (ID, E-value, alignment start - end position)
	start	end	nt	aa	ORF, protein encoded, or mass	Species	Accession	Aa identity	E Value	Alignment length in aa (Start-end position)		
												transmembrane_regions(tmhmm,-1.0,30-48;-1.0,58-78;-1.0,84-104)
30	22,670	23,491	822	273	hypothetical protein H665_p044	Ostreococcus tauri virus 1	YP_003212868	29.60%	7.83E-23	256(4-259)		
31	23,497	24,312	816	271	hypothetical protein OMVG_00221	Ostreococcus lucimarinus	AFK66216	28.60%	1.92E-23	256(3-258)		
32	24,285	25,376	1,092	363	GDP-mannose 4,6-dehydratase	Sporosarcina newyorkensis 2681	ZP_08678009	56.20%	3.63E-135	339(22-360)	GDP_MD_SDR_e(cd05260,3.60e-153 ,336,25-360)	signal-peptide(SignalP-NN(euk),-1.0,1-32)
											Gmd(COG1089,4.42e-173,338,22-359)	gmd: GDP-mannose 4,6-dehydratase(TIGR01472,1.3E-136,23-360)
33	25,376	26,332	957	318	hypothetical protein MpV1_229c	Micromonas sp. RCC1109 virus MpV1	YP_004062112	45.30%	1.05E-83	299(3-301)	GT1_YqgM_like(cd03801,1.28e-12,161,90-250)	signal-peptide(SignalP-NN(euk),-1.0,1-22)
					glycosyl transferase, group 1	Jannaschia sp. CCS1	YP_512219	26.60%	9.84E-14	217(70-286)	RfaG(COG0438,1.72e-11,146,99-244)	UDP-Glycosyltransferase/glycogen phosphorylase(SSF53756,2.2E-16,13-244)
34	26,599	26,847	249	82	hypothetical protein OLOG_00122	Ostreococcus lucimarinus virus OlV4	AET84583	42.60%	2.33E-06	68(2-69)		signal-peptide(SignalP-NN(euk),-1.0,1-27)
												transmembrane_regions(tmhmm,-1.0,5-23;-1.0,47-67)
35	26,863	27,306	444	147	hypothetical protein MPWG_00022	Micromonas pusilla virus PL1	AET43512	30.00%	3.96E-07	95(50-144)		
36	27,370	27,642	273	90	ubiquitin	Gracilaria lemaneiformis	AAY41882	92.10%	2.84E-44	76(13-88)	Ubiquitin(cd01803,1.57e-44,76,13-88)	signal-peptide(SignalP-NN(euk),-1.0,1-22)
												UBIQUITIN_2(PS50053,30.256,13-88)
37	27,666	28,205	540	179	predicted protein	Leptosphaeria maculans JN3	XP_003839359	81.30%	1.47E-04	32(143-174)		
38	28,223	28,717	495	164	putative membrane protein	Emiliania huxleyi virus 99B1	CAZ69772	29.70%	1.10E-07	157(7-163)	DUF3592(pfam12158,1.21e-03,116,21-136)	signal-peptide(SignalP-NN(euk),-1.0,1-34)

ID	Position		Length		BlastP hit in GenBank nr and/or viruses data set						NCBI conserved domain (ID, E-value, alignment length in aa, alignment start - end position)	Interproscan matches (ID, E-value, alignment start - end position)
	start	end	nt	aa	ORF, protein encoded, or mass	Species	Accession	Aa identity	E Value	Alignment length in aa (Start-end position)		
												transmembrane_regions(tmhmm,-1.0,19-39;-1.0,117-137)
					probable FAD-linked sulfhydryl oxidase	Acanthamoeba polyphaga mimivirus	YP_003986873	37.80%	1.41E-13	97(1-97)	Evr1_Alr(pfam04777,3.65e-19,91,8-98)	FAD-dependent thiol oxidase(SSF69000,2.4E-21,1-110)
39	28,736	29,149	414	137	sulfhydryl oxidase	Chlamydomonas reinhardtii	XP_001703298	38.50%	1.83E-13	96(7-102)		ALR/ERV(PTHR12645,2.0E-14,26-129)
												signal-peptide(SignalP-NN(euk),-1.0,1-25)
												transmembrane_regions(tmhmm,-1.0,115-135)
					hypothetical protein EhV003	Emiliania huxleyi virus 86	YP_293757	36.60%	2.31E-13	100(14-113)		signal-peptide(SignalP-NN(euk),-1.0,1-51)
40	29,155	29,529	375	124								transmembrane_regions(tmhmm,-1.0,14-32;-1.0,37-57;-1.0,103-118)
41	29,534	30,025	492	163								
					hypothetical protein MpV1_224c	Micromonas sp. RCC1109 virus MpV1	YP_004062107.1	52.00%	0	1046(4-1049)	HATPase_c(pfam02518,1.95e-05,106,45-150)	Ribosomal protein S5 domain 2-like(SSF54211,6.3E-24,218-368)
	30,055	33,204	3,150	1049	DNA topoisomerase II	Encephalitozoon intestinalis ATCC 50506	XP_003072680	44.20%	0	1044(3-1046)	TopoIIA_Trans_ScTopoIIA(cd03481,1.04e-30,110,251-360)	Arginine repressor C-terminal-like domain(G3DSA:3.30.1360.40,3.9E-10,819-888)
42											TOPRIM_TopoIIA(cd03365,1.16e-62,118,401-518)	DNA TOPOISOMERASE/GYRASE(PTHR10169,0.0,2-1049)
											TOP4c(cd00187,8.20e-102,413,637-1049)	
											PLN03128(PLN03128,0,1048,2-1049)	

ID	Position		Length		BlastP hit in GenBank nr and/or viruses data set						NCBI conserved domain (ID, E-value, alignment length in aa, alignment start - end position)	Interproscan matches (ID, E-value, alignment start - end position)
	start	end	nt	aa	ORF, protein encoded, or mass	Species	Accession	Aa identity	E Value	Alignment length in aa (Start-end position)		
43	33,211	35,442	2,232	743	hypothetical protein MPWG_00028	Micromonas pusilla virus PL1	AET43518	31.30%	1.65E-55	420(318-737)	Capsid_NCLDV(pfam04451,2.89e-21,203,538-740)	Capsid_NCLDV(PF04451,5.4E-25,530-739)<br>no description(G3DSA:2.70.9.10,3.9E-24,351-543)<br>Group II dsDNA viruses VP(SSF49749,3.3E-32,340-548;4.6E-23,549-743)<br>no description(G3DSA:2.70.9.20,3.3E-24,561-742)
44	35,354	35,791	438	145	hypothetical protein MpV1_222	Micromonas sp. RCC1109 virus MpV1	YP_004062105	54.50%	1.03E-45	128(1-128)		
45	35,807	36,133	327	108	hypothetical protein MPWG_00030	Micromonas pusilla virus PL1	AET43520	26.50%	1.47E-08	82(1-82)		
46	36,135	38,726	2,592	863	DNA polymerase	Micromonas pusilla virus SP1	AET84947.1	55.00%	0	833(1-833)	PolB(COG0417,4.73e-149,841,15-855)<br>POLBc, with user query added Superfamily(cl10023,3.28e-130,386,447-832)<br>DNA_polB_delta_exo(cd05777,3.32e-63,223,174-396)	DNA_pol_B_exo1(PF03104,2.0E-50,30-341)<br>DNA_pol_B(PF00136,2.0E-117,415-837)<br>Ribonuclease H-like(SSF53098,3.5E-81,20-431)
47	38,738	39,130	393	130								signal-peptide(SignalP-NN(euk),-1.0,1-26)<br>transmembrane_regions(tmhmm,-1.0,10-30;-1.0,66-86;-1.0,105-125)
48	39,146	39,580	435	144	hypothetical protein OlV1_207	Ostreococcus lucimarinus	YP_004061839	57.30%	8.90E-46	143(1-143)	trimeric_dUTPase(cd07557,3.56e-29,90,23-112)	dUTPase(PF00692,3.8E-34,12-143)
					dUTP diphosphatase, partial	Wallemia sebi CBS 633.66	EIM19898	52.80%	7.63E-38	142(2-143)		SUBFAMILY NOT NAMED(PTHR11241:SF0,3.6E-59,1-143)
49	39,642	40,265	624	207								

ID	Position		Length		BlastP hit in GenBank nr and/or viruses data set						NCBI conserved domain (ID, E-value, alignment length in aa, alignment start - end position)	Interproscan matches (ID, E-value, alignment start - end position)
	start	end	nt	aa	ORF, protein encoded, or mass	Species	Accession	Aa identity	E Value	Alignment length in aa (Start-end position)		
50	40,240	40,923	684	227	hypothetical protein ACD_34C00433G0002	uncultured bacterium	EKD88618	24.50%	4.50E-04	198(18-215)	Pyr-5-nucltdase(TIGR01993,4.84e-10,178,18-195)	HAD-like(SSF56784,7.8E-8,18-208)
51	41,086	42,186	1,101	366	ornithine decarboxylase 1	Chlamydomonas reinhardtii	XP_001697502	45.80%	2.39E-102	356(11-366)	PLPDE_III_ODC(cd00622,1.91e-144,350,17-366)	Alanine racemase C-terminal domain-like(SSF50621,1.5E-23,232-357)
											LysA(COG0019,4.66e-70,357,1-357)	ARGININE/DIAMINOPIMELATE/ORNITHINE DECARBOXYLASE(PTHR11482,3.6E-127,17-357)
												PLP-binding barrel(SSF51419,1.8E-58,17-259)
52	42,503	43,207	705	234	von Willebrand factor A	Intrasporangium calvum DSM 43043	YP_004097376	45.70%	1.52E-40	162(54-215)	vWFA(cd00198,2.17e-06,106,51-156)	signal-peptide(SignalP-NN(euk),-1.0,1-22)
												vWA-like(SSF53300,5.8E-13,48-221)
53	43,155	43,667	513	170	putative deoxynucleoside monophosphate kinase	Cafeteria roenbergensis	YP_003969712	29.50%	1.64E-14	162(1-162)	deoxynucleoside monophosphate kinase(PHA02575,8.79e-06,74,1-74;1.50e-03,63,98-160)	
					deoxynucleotide monophosphate kinase, putative	Marinobacter algicola DG893	ZP_01894233	32.40%	6.19E-14	152(2-153)		P-loop containing nucleoside triphosphate hydrolases(SSF52540,2.7E-6,1-160)
54	43,668	44,078	411	136								signal-peptide(SignalP-NN(euk),-1.0,1-26)
												transmembrane_regions(tmhmm,-1.0,4-26;-1.0,41-63)
55	44,078	44,389	312	103	hypothetical protein PBCVMA1D_454L	Paramecium bursaria Chlorella virus MA-1D	AGE54931	64.30%	6.30E-24	70(24-93)		
					Tlr 6Fp protein	Tetrahymena	AAL73477	42.90%	2.44E-12	70(24-93)		

ID	Position		Length		BlastP hit in GenBank nr and/or viruses data set						NCBI conserved domain (ID, E-value, alignment length in aa, alignment start - end position)	Interproscan matches (ID, E-value, alignment start - end position)
	start	end	nt	aa	ORF, protein encoded, or mass	Species	Accession	Aa identity	E Value	Alignment length in aa (Start-end position)		
						thermophila						
56	44,449	45,627	1,179	392	hypothetical protein OlV1_184c	Ostreococcus lucimarinus virus OlV1	YP_004061816	31.40%	9.88E-38	335(56-390)	RFC1(pfam08519,6.06e-05,124,253-376)	DNA polymerase III clamp loader subunits, C-terminal domain(SSF48019,5.7E-7,233-288)
					hypothetical protein	Paramecium tetraurelia strain d4-2	XP_001427741	32.90%	6.56E-02	66(230-295)		
57	45,579	46,163	585	194	hypothetical protein BpV1_171c	Bathycoccus sp. RCC1105 virus BpV1	YP_004061601	47.30%	2.06E-52	184(9-192)	S14_ClpP_2(cd07017,1.56e-15,134,59-192)	ATP-DEPENDENT CLP PROTEASE PROTEOLYTIC SUBUNIT(PTHR10381,7.1E-16,52-193)
					ATP-dependent Clp protease proteolytic subunit	Clostridium sp. DL-VIII	ZP_09206655	33.10%	7.13E-14	166(28-193)		CLP_protease(PF00574,2.4E-24,60-193)
58	46,492	47,478	987	328	hypothetical protein OtV6_158	Ostreococcus tauri virus RT-2011	AFC35066	44.10%	3.76E-72	317(3-319)	TFIIB(pfam00382,2.73e-03,55,148-202)	
					transcription initiation factor IIB	Rhizoctonia solani AG-1 IA	ELU45667	27.10%	2.68E-20	273(54-326)	SUA7(COG1405,1.44e-25,260,54-313)	TRANSCRIPTION INITIATION FACTOR IIB(PTHR11618:SF13,2.8E-32,54-314)
59	47,475	48,227	753	250	hypothetical protein MpV1_158	Micromonas sp. RCC1109 virus MpV1	YP_004062041	65.80%	4.11E-118	240(7-246)	Glycos_transf_2(pfam00535,5.88e-04,114,55-168)	signal-peptide(SignalP-NN(euk),-1.0,1-17)
					hypothetical protein Vapar_0507	Variovorax paradoxus S110	YP_002942436	37.00%	1.00E-47			Nucleotide-diphospho-sugar transferases(SSF53448,7.5E-10,5-236)
60	48,235	48,870	636	211	hypothetical protein MpV1_159	Micromonas sp. RCC1109 virus MpV1	YP_004062042	41.50%	7.88E-46	202(9-210)	GerC3_HepT(TIGR02748,7.16e-03,82,107-188)	
61	48,900	49,751	852	283	hypothetical protein H665_p156	Ostreococcus tauri virus 1	YP_003212979	39.70%	7.98E-58	280(2-281)	Adenylation_mRNA_capping(cd07895,4.31e-39,180,15-194)	mRNA_cap_C(PF03919,8.0E-8,232-280)
											mRNA_cap_C(pfam03919,1.80e-05,83,199-281)	Nucleic acid-binding proteins(SSF50249,2.1E-15,196-283)

ID	Position		Length		BlastP hit in GenBank nr and/or viruses data set						NCBI conserved domain (ID, E-value, alignment length in aa, alignment start - end position)	Interproscan matches (ID, E-value, alignment start - end position)
	start	end	nt	aa	ORF, protein encoded, or mass	Species	Accession	Aa identity	E Value	Alignment length in aa (Start-end position)		
												DNA ligase/mRNA capping enzyme, catalytic domain(SSF56091,4.1E-28,1-195)
62	49,758	49,997	240	79								
63	49,994	50,773	780	259	hypothetical protein OtV6_162c	Ostreococcus tauri virus RT-2011	AFC35070	38.50%	7.54E-57	256(1-256)	Peptidase_C19A(cd02657,1.28e-20,243,3-245)	UCH_2_3(PS50235,17.556,2-259)
					PREDICTED: ubiquitin carboxyl-terminal hydrolase 17-like protein 2-like	Cricetulus griseus	XP_003512737	26.80%	3.45E-15	255(3-257)	UCH(pfam00443,1.16e-25,244,2-245)	Cysteine proteinases(SSF54001,8.2E-42,1-258)
64	50,815	51,402	588	195	hypothetical protein BpV2_156	Bathycoccus sp.RCC1105 virus BpV2	ADQ91323	42.60%	8.21E-44	185(11-195)		mRNA_triPase(PF02940,2.1E-14,33-169)
					hypothetical protein MGL_2917	Malassezia globosa CBS 7966	XP_001729931	25.20%	1.06E-04	104(72-175)	CYTH-like_mRNA_RTPase(cd07470,2.20e-12,113,67-179)	CYTH-like phosphatases(SSF55154,1.0E-11,22-183)
65	51,406	54,216	2,811	936								
66	53,958	55,889	1,932	643	hypothetical protein OtV2_151	Ostreococcus tauri virus 2	YP_004063584	60.80%	0	476(168-643)	COG3378(COG3378,7.44e-10,159,366-524)	SF3_HELICASE_1(PS51206,11.932,328-502)
												P-loop containing nucleoside triphosphate hydrolases(SSF52540,1.0E-11,342-519)
67	55,913	56,200	288	95	hypothetical protein MPWG_00084	Micromonas pusilla virus PL1	AET43573	43.50%	2.86E-06	69(16-84)		
68	56,279	57,634	1,356	451	hypothetical protein OsV5_190f	Ostreococcus virus OsV5	YP_001648266	63.70%	0	451(1-451)	Capsid_NCLDV(pfam04451,9.22e-60,248,200-447)	Capsid_NCLDV(PF04451,1.8E-67,198-447)
												Adenovirus PII,hexon,subdomain4(G3DSA:2.70.9.10,3.1E-52 35-220)

ID	Position		Length		BlastP hit in GenBank nr and/or viruses data set						NCBI conserved domain (ID, E-value, alignment length in aa, alignment start - end position)	Interproscan matches (ID, E-value, alignment start - end position)
	start	end	nt	aa	ORF, protein encoded, or mass	Species	Accession	Aa identity	E Value	Alignment length in aa (Start-end position)		
												Group II dsDNA viruses VP(SSF49749,3.3E-55,25-219;7.9E-43,220-451)<br>African swine fever virus,p72,major capsid(G3DSA:2.70.9.20,1.8E-53 318-451)
69	57,730	58,044	315	104	S-adenosylmethionine decarboxylase proenzyme	Nitratiruptor sp. SB155-2	YP_001356990	35.80%	8.34E-16	92(2-93)	AdoMet_dc(pfam02675,1.02e-24,88,3-90)	S-adenosylmethionine decarboxylase(SSF56276,2.1E-18,3-104)
70	58,047	58,244	198	65								signal-peptide(SignalP-NN(euk),-1.0,1-28)<br>transmembrane_regions(tmhmm,-1.0,12-30;-1.0,40-58)
71	58,267	58,680	414	137								
72	58,877	59,479	603	200								transmembrane_regions(tmhmm,-1.0,165-187)
73	59,516	60,046	531	176	CBLC protein	Mus musculus	AAK01405	37.90%	5.57E-07	63(106-168)	RING(cd00162,2.57e-09,41,126-166)<br>zf-C3HC4_2(pfam13923,5.22e-09,41,124-164)	CBL(PTHR23007,5.2E-5,127-163)<br>RING/U-box(SSF57850,8.3E-12,82-169)
74	60,335	60,694	360	119	predicted protein	Physcomitrella patens subsp. patens	XP_001768243	48.10%	6.32E-20	81(3-83)	DUF814(pfam05670,2.99e-21,70,6-75)	DUF814(PF05670,1.2E-18,7-85)<br>SUBFAMILY NOT NAMED(PTHR13049:SF0,1.1E-5,4-54)
75	61,206	61,547	342	113								signal-peptide(SignalP-NN(euk),-1.0,1-29)<br>transmembrane_regions(tmhmm,-1.0,10-28;-1.0,48-66;-1.0,80-100)
76	61,594	62,025	432	143								

ID	Position		Length		BlastP hit in GenBank nr and/or viruses data set						NCBI conserved domain (ID, E-value, alignment length in aa, alignment start - end position)	Interproscan matches (ID, E-value, alignment start - end position)
	start	end	nt	aa	ORF, protein encoded, or mass	Species	Accession	Aa identity	E Value	Alignment length in aa (Start-end position)		
77	62,015	62,692	678	225	PREDICTED: procollagen galactosyltransferase 2-like	Xenopus (Silurana) tropicalis	XP_002933810	27.70%	2.30E-13	181(1-181)	Glyco_transf_25(cd06532,4.80e-19,149,5-153)	PROCOLLAGEN-LYSINE,2-OXOGLUTARATE 5-DIOXYGENASE/GLYCOSYLTRANSFERASE 25 FAMILY MEMBER(PTHR10730,2.5E-7,1-103) transmembrane_regions(tmhmm,-1.0,193-215)
78	62,693	63,043	351	116	LPS glycosyltransferase	Burkholderia cenocepacia BC7	ZP_21158759	38.50%	1.78E-11	87(8-94)	Glyco_transf_25(cd06532,9.82e-22,88,5-92)	GLYCOSYLTRANSFERASE 25 FAMILY MEMBER(PTHR10730:SF2,4.1E-8,1-87) Glyco_transf_25(PF01755,1.1E-17,5-103)
79	63,067	63,384	318	105							Glyco_transf_25(cd06532,4.45e-03,28,8-35)	signal-peptide(SignalP-NN(euk),-1.0,1-2) transmembrane_regions(tmhmm,-1.0,42-62;-1.0,81-99)
80	63,389	63,796	408	135	hypothetical protein	Theileria parva strain Muguga	XP_765348	32.90%	2.40E-04	81(50-130)	CRAL_TRIO(pfam00650,5.51e-03,73,63-135)	CRAL_TRIO(PF00650,3.4E-6,63-133)
81	63,782	64,195	414	137								
82	64,252	65,208	957	318	hypothetical protein MpV1_155c	Micromonas sp. RCC1109 virus MpV1	YP_004062038	67.40%	8.41E-154	316(3-318)	RNRR2(cd01049,3.78e-109,275,13-287)	RIBONUCLEOSIDE-DIPHOSPHATE REDUCTASE SMALL CHAIN(PTHR23409,7.4E-191,1-318)
					predicted protein	Ostreococcus lucimarinus CCE9901	XP_001419276	60.80%	3.34E-145	318(1-318)		Ferritin-like(SSF47240,2.0E-113,1-303)
83	65,205	65,495	291	96	hypothetical protein BpV1_143	Bathycoccus sp. RCC1105 virus BpV1	YP_004061573	41.30%	6.36E-09	63(8-70)		transmembrane_regions(tmhmm,-1.0,157-177) signal-peptide(SignalP-NN(euk),-1.0,1-17)

ID	Position		Length		BlastP hit in GenBank nr and/or viruses data set						NCBI conserved domain (ID, E-value, alignment length in aa, alignment start - end position)	Interproscan matches (ID, E-value, alignment start - end position)
	start	end	nt	aa	ORF, protein encoded, or mass	Species	Accession	Aa identity	E Value	Alignment length in aa (Start-end position)		
												transmembrane_regions(tmhmm,-1.0,10-30;-1.0,44-64)
84	65,513	66,496	984	327								
85	66,571	67,266	696	231	hypothetical protein MPVG_00083	Micromonas pusilla virus 12T	YP_007676151	46.90%	6.45E-51	206(1-206)		DUF2738(PF10927,9.0E-7,34-230)
					EsV-1-76	Ectocarpus siliculosus	CBN80379	26.00%	4.00E-06			
86	67,311	69,416	2,106	701	hypothetical protein MpV1_151c	Micromonas sp. RCC1109 virus MpV1	YP_004062034	28.30%	2.19E-19	353(126-478)	PKc(cd00180,5.82e-17,199,139-337)	PROTEIN_KINASE_DOM(PS50011,15.722,130-503)
					Serine/threonine-protein kinase ULK2	Harpegnathos saltator	EFN88339	30.90%	5.85E-06	177(139-315)		Serine/Threonine protein kinases, catalytic(SM00220,8.1E-4,130-487)
87	69,421	69,714	294	97	hypothetical protein H665_p144	Ostreococcus tauri virus 1	YP_003212967	37.60%	5.75E-09	84(14-97)		GLUTAREDOXIN_2(PS51354,9.516,27-97)
					glutaredoxin	Methanosaeta thermophila PT	YP_843336	32.40%	9.93E-05	62(35-96)	NrdH(cd02976,1.72e-10,58,39-96)	Thioredoxin-like(SSF52833,7.2E-10,26-96)
												signal-peptide(SignalP-NN(euk),-1.0,1-28)
												transmembrane_regions(tmhmm,-1.0,10-30)
88	69,709	69,912	204	67	hypothetical protein OtV6_145	Ostreococcus tauri virus RT-2011	AFC35053	36.90%	2.75E-09	65(2-66)		signal-peptide(SignalP-NN(euk),-1.0,1-31)
												transmembrane_regions(tmhmm,-1.0,15-35)
89	69,919	70,758	840	279	hypothetical protein BpV2_144c	Bathycoccus sp. RCC1105 virus BpV2	ADQ91311	40.90%	2.48E-53	252(24-275)		
					hypothetical protein VOLCADRAFT_119457	Volvox carteri f. nagariensis	XP_002956623	28.90%	1.97E-14	251(27-277)		

ID	Position		Length		BlastP hit in GenBank nr and/or viruses data set						NCBI conserved domain (ID, E-value, alignment length in aa, alignment start - end position)	Interproscan matches (ID, E-value, alignment start - end position)
	start	end	nt	aa	ORF, protein encoded, or mass	Species	Accession	Aa identity	E Value	Alignment length in aa (Start-end position)		
90	70,776	71,249	474	157	hypothetical protein OtV6_143	Ostreococcus tauri virus RT-2011	AFC35051	30.70%	9.53E-13	124(30-153)		zf-RING_2(PF13639,9.0E-12,31-77)
					PREDICTED: RING finger protein 141-like	Bombus impatiens	XP_003487428	41.20%	1.22E-07	50(29-78)	RING(cd00162,1.92e-08,48,31-78)	RING FINGER PROTEIN 38(PTHR14155:SF2,2.9E-8,31-101)
91	71,271	71,609	339	112	hypothetical protein TCSYLVIO_005045	Trypanosoma cruzi	EKG03892	48.10%	3.35E-09	52(28-79)	RING(cd00162,3.80e-06,41,31-71)	zf-RING_2(PF13639,4.6E-13,31-72)
											zf-C3HC4_2(pfam13923,2.37e-06,44,29-72)	RING FINGER PROTEIN 24-RELATED(PTHR22766,2.8E-11,31-84)
												RING/U-box(SSF57850,9.3E-17,25-94)
92	71,500	72,162	663	220	kinase protein	Acanthocystis turfacea Chlorella virus NTS-1	AGE57698	37.00%	9.20E-25	179(1-179)	dNK(cd01673,1.16e-30,166,3-168)	Deoxynucleoside kinase(PIRSF000705,3.7E-14,1-197)
					Deoxynucleoside kinase	Lepeophtheirus salmonis	ADD38206	28.40%	4.40E-20	201(4-204)	Tmk(COG0125,4.27e-12,181,4-184)	P-loop containing nucleoside triphosphate hydrolases(SSF52540,3.0E-33,1-184)
93	72,189	72,824	636	211	hypothetical protein CHLNCDRAFT_133736	Chlorella variabilis	EFN55609	31.40%	5.53E-07	151(59-209)	Glyco_transf_25(cd06532,2.74e-05,77,25-101)	Pox_P35(PF03213,5.3E-6,81-184)
					hypothetical protein BpV1_162c	Bathycoccus sp. RCC1105 virus BpV1	YP_004061592	29.60%	4.27E-03	95(68-162)	Glyco_transf_25 Superfamily(cl01298,4.04e-04,110,61-170)	signal-peptide(SignalP-NN(euk),-1.0,1-21)
94	72,833	73,555	723	240	AGAP001005-PA	Anopheles gambiae str. PEST	XP_309198	64.00%	9.26E-05	47(51-97)		signal-peptide(SignalP-NN(euk),-1.0,1-27)
												transmembrane_regions(tmhmm,-1.0,10-30)
95	73,579	73,803	225	74	hypothetical protein BpV2_142	Bathycoccus sp. RCC1105 virus BpV2	ADQ91309	49.30%	1.17E-14	63(1-63)		
96	73,809	74,597	789	262	hypothetical protein MpV1_145	Micromonas sp. RCC1109 virus	YP_004062028	47.20%	1.83E-78	261(2-262)	TBP_TLF(cd00652,4.28e-06,71,141-211)	Beta2-adaptin/TBP,C-terminal domain(G3DSA:3.30.310.10,1.4E-8,137-

ID	Position		Length		BlastP hit in GenBank nr and/or viruses data set						NCBI conserved domain (ID, E-value, alignment length in aa, alignment start - end position)	Interproscan matches (ID, E-value, alignment start - end position)
	start	end	nt	aa	ORF, protein encoded, or mass	Species	Accession	Aa identity	E Value	Alignment length in aa (Start-end position)		
						MpV1						229)
					hypothetical protein GUITHDRAFT_160489	Guillardia theta CCMP2712	EKX55535	27.90%	8.75E-07	138(96-233)		TATA-box binding protein-like(SSF55945,6.8E-12,134-243)
97	74,608	75,615	1,008	335								
98	75,588	76,334	747	248								
99	76,412	77,827	1,416	471	hypothetical protein OlV1_147c	Ostreococcus lucimarinus virus OlV1	YP_004061780	55.70%	0	466(5-470)	DEXDc(cd00046,1.27e-14,130,29-158)<br>HELICc(cd00079,9.70e-22,121,291-411)<br>SNF2_N(pfam00176,1.24e-46,263,10-272)	HELICASE_CTER(PS51194,14.926,294-450)<br>HELICASE_ATP_BIND_1(PS51192,14.841,21-176)<br>SWI/SNF-RELATED MATRIX-ASSOCIATED ACTIN-DEPENDENT REGULATOR OF CHROMATIN SUBFAMILY-RELATED(PTHR10799,1.8E-106,9-450)<br>P-loop containing nucleoside triphosphate hydrolases(SSF52540,1.1E-49,8-195;6.3E-47,196-455)
100	77,870	78,136	267	88	FirrV-1-A38	Feldmannia irregularis virus a	AAR26862	43.60%	4.70E-10	87(1-87)		
					dinoflagellate viral nucleoprotein DVNP.6	Hematodinium sp. SG-2012	AFY23230	46.30%	1.67E-07	83(6-88)		
101	78,247	78,753	507	168	hypothetical protein ATCVMN08101_569R	Acanthocystis turfacea Chlorella virus MN0810.1	AGE55688	24.50%	7.53E-02	132(29-160)		
102	78,781	79,248	468	155								

ID	Position		Length		BlastP hit in GenBank nr and/or viruses data set						NCBI conserved domain (ID, E-value, alignment length in aa, alignment start - end position)	Interproscan matches (ID, E-value, alignment start - end position)
	start	end	nt	aa	ORF, protein encoded, or mass	Species	Accession	Aa identity	E Value	Alignment length in aa (Start-end position)		
103	79,220	79,528	309	102	hypothetical protein	Marine Group II euryarchaeote SCGC AB-629-J06	WP_018035887	47.00%	2.00E-23	93(2-94)	HNHc(cd00085,7.78e-07,51,20-70)	HNH(PF01844,1.5E-8,40-72)
					hypothetical protein crov535	Cafeteria roenbergensis virus BV-PW1	YP_003970168	35.40%	1.76E-07	81(2-82)		HNH nucleases(SM00507,0.0027,16-69)
104	79,534	79,911	378	125								
105	79,938	82,187	2,250	749	ribonucleoside-diphosphate reductase, alpha subunit	Microscilla marina ATCC 23134	ZP_01688231	59.00%	0	728(1-728)	ATP-cone(pfam03477,5.47e-14,86,1-86)	ATP_CONE(PS51161,16.237,1-89)
					ribonucleoside diphosphate reductase	Ostreococcus lucimarinus virus OlV3	AFK66128	61.60%	0	749(1-749)	RNR_I(cd01679,0,571,147-717)	RIBONUCLEOSIDE-DIPHOSPHATE REDUCTASE LARGE CHAIN(PTHR11573:SF6,0.0,1-728)
											PLN02437(PLN02437,0,749,1-749)	PFL-like glycyl radical enzymes(SSF51998,6.3E-186,195-728)
106	82,211	82,411	201	66								signal-peptide(SignalP-NN(euk),-1.0,1-23)
												transmembrane_regions(tmhmm,-1.0,15-33;-1.0,39-57)
107	82,398	83,129	732	243	hypothetical protein OtV6_177	Ostreococcus tauri virus RT-2011	AFC35085	58.60%	2.81E-100	236(5-240)		signal-peptide(SignalP-NN(euk),-1.0,1-23)
												transmembrane_regions(tmhmm,-1.0,5-23)
108	83,178	83,513	336	111								signal-peptide(SignalP-NN(euk),-1.0,1-16)
109	83,536	83,919	384	127	hypothetical protein MPVG_00097	Micromonas pusilla virus 12T	YP_007676165	44.40%	3.27E-27	126(2-127)		
110	83,940	84,164	225	74								transmembrane_regions(tmhmm,-1.0,9-29;-1.0,43-63)

ID	Position		Length		BlastP hit in GenBank nr and/or viruses data set						NCBI conserved domain (ID, E-value, alignment length in aa, alignment start - end position)	Interproscan matches (ID, E-value, alignment start - end position)
	start	end	nt	aa	ORF, protein encoded, or mass	Species	Accession	Aa identity	E Value	Alignment length in aa (Start-end position)		
111	84,190	84,510	321	106	hypothetical protein OlV1_140c	Ostreococcus lucimarinus virus OlV1	YP_004061773	69.40%	3.01E-43	98(1-98)	NTP-PPase_SsMazG(cd11535,2.00e-10,75,10-84)	Predicted pyrophosphohydrolase, MazG-related, Bacilli type(PIRSF036521,2.2E-6,1-101)
					MazG nucleotide pyrophosphohydrolase	Ktedonobacter racemifer DSM 44963	ZP_06973402	32.60%	3.32E-09	92(8-99)		all-alpha NTP pyrophosphatases(SSF101386,7.6E-15,1-97)
112	84,510	84,926	417	138								transmembrane_regions(tmhmm,-1.0,119-133)
113	84,916	85,110	195	64	hypothetical protein MPXG_00120	Micromonas pusilla virus SP1	AET84918	42.60%	1.33E-02	47(1-47)		zf-AN1(PF01428,1.6E-5,3-42)
					hypothetical protein CHLNCDRAFT_144583	Chlorella variabilis	EFN56686	41.30%	7.86E-02	62(3-64)		ZF_BBOX(PS50119,8.617,1-33)
114	85,112	85,831	720	239	hypothetical protein BpV2_126	Bathycoccus sp. RCC1105 virus BpV2	ADQ91293	57.40%	1.12E-73	198(21-218)	YqaJ(pfam09588,9.97e-35,125,40-164)	Restriction endonuclease-like(SSF52980,1.1E-46,16-226)
					PBCV-1 exonuclease (ISS)	Ostreococcus tauri	XP_003081163	38.00%	6.00E-25			YqaJ(PF09588,7.7E-30,40-166)
115	85,695	86,471	777	258	ribonuclease III	Micromonas pusilla virus PL1	AET43609	60.60%	4.15E-89	211(12-222)	RIBOc(cd00593,3.15e-40,126,24-149)	RNase III domain-like(SSF69065,5.4E-38,5-163)
											DSRM(cd00048,1.53e-15,62,160-221)	DS_RBD(PS50137,11.82,160-225)
											RNaseIII(TIGR02191,1.88e-65,205,17-221)	RNase_III(MF_00104,24.211,12-220)
116	86,477	86,986	510	169								signal-peptide(SignalP-NN(euk),-1.0,1-23)
												transmembrane_regions(tmhmm,-1.0,14-34)
117	87,019	87,408	390	129								transmembrane_regions(tmhmm,-1.0,108-128)

ID	Position		Length		BlastP hit in GenBank nr and/or viruses data set						NCBI conserved domain (ID, E-value, alignment length in aa, alignment start - end position)	Interproscan matches (ID, E-value, alignment start - end position)
	start	end	nt	aa	ORF, protein encoded, or mass	Species	Accession	Aa identity	E Value	Alignment length in aa (Start-end position)		
118	87,442	87,993	552	183	hypothetical protein OLOG_00166	Ostreococcus lucimarinus virus OlV4	AET84626	37.80%	1.48E-28	173(1-173)		
119	88,043	88,480	438	145	hypothetical protein MPXG_00053	Micromonas pusilla virus SP1	AET84851	45.60%	3.21E-29	133(3-135)		signal-peptide(SignalP-NN(euk),-1.0,1-26)
												transmembrane_regions(tmhmm,-1.0,5-20)
120	88,516	88,881	366	121	hypothetical protein MPWG_00126	Micromonas pusilla virus PL1	AET43614	38.50%	1.32E-18	117(5-121)		
121	88,894	89,265	372	123								
					hypothetical protein OtV6_122c	Ostreococcus tauri virus RT-2011	AFC35030	51.20%	1.64E-26	126(3-128)		DUF3339(PF11820,3.2E-7,70-124)
122	89,266	89,652	387	128	hypothetical protein PRUPE_ppa026813mg	Prunus persica	EMJ18658	33.60%	1.28E-05	115(6-120)		signal-peptide(SignalP-NN(euk),-1.0,1-25)
												transmembrane_regions(tmhmm,-1.0,9-29;-1.0,35-55;-1.0,70-88;-1.0,102-120)
123	89,655	90,446	792	263	hypothetical protein MPXG_00049	Micromonas pusilla virus SP1	AET84847	37.70%	2.62E-51	256(1-256)	Pat_ExoU_VipD_like(cd07207,2.90e-09,68,106-173)	Patatin(PF01734,1.1E-12,9-183)
					hypothetical protein F902_04067	Acinetobacter sp. CIP 70.18	ENX53198	27.10%	5.17E-10	185(7-191)	RssA(COG1752,5.33e-05,151,101-251)	FabD/lysophospholipase-like(SSF52151,5.4E-25,1-193)
												signal-peptide(SignalP-NN(euk),-1.0,1-28)
												transmembrane_regions(tmhmm,-1.0,5-25;-1.0,31-51)
124	90,455	90,793	339	112	hypothetical protein OtV6_119c	Ostreococcus tauri virus RT-2011	AFC35027	45.80%	1.02E-11	70(43-112)		
					hypothetical protein Ngar_c25890	Candidatus Nitrososphaera gargensis Ga9.2	YP_006863168	32.70%	2.76E-02	93(6-98)		

ID	Position		Length		BlastP hit in GenBank nr and/or viruses data set						NCBI conserved domain (ID, E-value, alignment length in aa, alignment start - end position)	Interproscan matches (ID, E-value, alignment start - end position)
	start	end	nt	aa	ORF, protein encoded, or mass	Species	Accession	Aa identity	E Value	Alignment length in aa (Start-end position)		
125	90,786	91,337	552	183								
126	91,346	92,809	1,464	487								transmembrane_regions(tmhmm,-1.0,195-215;-1.0,238-258)
127	92,833	93,180	348	115	hypothetical protein BpV1_099c	Bathycoccus sp. RCC1105 virus BpV1	YP_004061529	57.80%	6.43E-38	108(5-112)	PDDEXK_3(pfam13366,1.71e-32,89,11-99)	PDDEXK_3(PF13366,1.5E-31,5-107)
					hypothetical protein HMPREF0659_A6051	Prevotella melaninogenica ATCC 25845	YP_003814119	46.30%	1.70E-20	95(5-99)		
128	93,167	94,072	906	301	hypothetical protein OLOG_00199	Ostreococcus lucimarinus virus OlV4	AET84656	53.80%	6.32E-102	286(15-300)		Pox_VLTF3(PF04947,5.6E-55,130-299)
129	94,101	94,268	168	55								
130	94,254	94,496	243	80								
131	94,528	95,265	738	245	hypothetical protein OsV5_115f	Ostreococcus virus OsV5	YP_001648192	53.90%	2.94E-92	244(1-244)	PCNA(cd00577,7.41e-50,242,4-245)	PROLIFERATING CELL NUCLEAR ANTIGEN(PTHR11352,8.9E-51,1-245)
					proliferating cell nuclear antigen II	Nicotiana tabacum	AAD19905	32.60%	2.39E-45	245(1-245)	PHA03383(PHA03383,2.87e-83,244,1-244)	DNA clamp(SSF55979,5.3E-30,1-124;4.2E-23,125-244)
132	95,289	95,690	402	133	hypothetical protein BpV2_094	Bathycoccus sp. RCC1105 virus BpV2	ADQ91261	44.70%	2.62E-33	128(1-128)		
133	95,740	96,186	447	148								signal-peptide(SignalP-NN(euk),-1.0,1-23) transmembrane_regions(tmhmm,-1.0,5-25)
134	96,214	96,582	369	122	hypothetical protein OtV2_097	Ostreococcus tauri virus 2	YP_004063530	39.40%	6.48E-21	121(1-121)		
					hypothetical protein VOLCADRAFT_99764	Volvox carteri f. nagariensis	XP_002958512	27.80%	7.93E-02	78(14-91)		

ID	Position		Length		BlastP hit in GenBank nr and/or viruses data set						NCBI conserved domain (ID, E-value, alignment length in aa, alignment start - end position)	Interproscan matches (ID, E-value, alignment start - end position)
	start	end	nt	aa	ORF, protein encoded, or mass	Species	Accession	Aa identity	E Value	Alignment length in aa (Start-end position)		
135	96,627	97,433	807	268	hypothetical protein BpV2_091c	Bathycoccus sp. RCC1105 virus BpV2	ADQ91258	37.10%	1.49E-38	225(4-228)		
136	97,457	97,669	213	70	hypothetical protein MPVG_00125	Micromonas pusilla virus 12T	YP_007676191	48.60%	1.56E-18	70(1-70)		transmembrane_regions(tmhmm,-1.0,10-25;-1.0,44-64)
137	97,693	98,166	474	157	hypothetical protein MpV1_101c	Micromonas sp. RCC1109 virus MpV1	YP_004061984	34.40%	8.62E-21	150(1-150)		transmembrane_regions(tmhmm,-1.0,91-109)
					EsV-1-42	Ectocarpus siliculosus	CBN80346	24.20%	3.75E-06	136(1-136)		
138	98,175	98,792	618	205	hypothetical protein MPVG_00127	Micromonas pusilla virus 12T	YP_007676193	40.60%	3.52E-44	176(2-177)		transmembrane_regions(tmhmm,-1.0,50-70)
139	98,817	99,086	270	89	hypothetical protein BpV1_087c	Bathycoccus sp. RCC1105 virus BpV1	YP_004061517	40.60%	6.41E-03	60(30-89)		
140	99,108	100,094	987	328	hypothetical protein MpV1_098c	Micromonas sp. RCC1109 virus MpV1	YP_004061981	36.40%	3.15E-47	327(2-328)		signal-peptide(SignalP-NN(euk),-1.0,1-20)
141	100,215	101,390	1,176	391	hypothetical protein OtV6_076	Ostreococcus tauri virus RT-2011	AFC34984	42.90%	7.60E-99	391(1-391)	Capsid_NCLDV(pfam04451,9.72e-31,182,206-387)	Capsid_NCLDV(PF04451,3.1E-28,205-386)
												Adenovirus PII,hexon,subdomain4(G3DSA:2.70.9.10,1.1E-33,35-223)
												Group II dsDNA viruses VP(SSF49749,5.0E-37,25-222;2.1E-15,223-391)
												African swine fever virus, p72, major capsid (G3DSA:2.70.9.20,5.3E-20,225-391)
142	101,513	102,070	558	185	hypothetical protein	Ostreococcus tauri	AFC34987	45.10%	9.38E-51	181(4-184)	Ap6A_hydrolase(cd03673,2.08e-	NUDIX(PS51462,9.302,4-168)

ID	Position		Length		BlastP hit in GenBank nr and/or viruses data set						NCBI conserved domain (ID, E-value, alignment length in aa, alignment start - end position)	Interproscan matches (ID, E-value, alignment start - end position)
	start	end	nt	aa	ORF, protein encoded, or mass	Species	Accession	Aa identity	E Value	Alignment length in aa (Start-end position)		
					OtV6_079	virus RT-2011					06,64,16-79)	
143	102,078	102,290	213	70								signal-peptide(SignalP-NN(euk),-1.0,1-4)
					glycosyl transferase group 8	Lactobacillus zeae KCTC 3804	ZP_09452772	25.70%	1.27E-02	118(17-134)	GT8_A4GalT_like(cd04194,1.29e-06,177,18-194)	Glyco_transf_8(PF01501,9.0E-10,72-213)
144	102,321	103,271	951	316								signal-peptide(SignalP-NN(euk),-1.0,1-19)
												Nucleotide-diphospho-sugar transferases(SSF53448,1.5E-14,1-234)
145	103,287	104,315	1029	342	hypothetical protein MpV1_083	Micromonas sp. RCC1109 virus MpV1	YP_004061966	36.40%	2.87E-57	292(13-304)		
					EsV-1-45	Ectocarpus siliculosus	CBN80349	27.00%	1.00E-04			Herpes_UL52(PF03121,6.4E-9,247-289)
146	104,603	104,932	330	109	hypothetical protein MPWG_00172	Micromonas pusilla virus PL1	AET43659	50.00%	3.65E-09	66(17-82)		
					YD repeat (two copies)	Lachnospiraceae bacterium 10-1	WP_016229777	28.00%	4.00E-11	268(40-307)	FN3(cd00063,1.91e-04,82,33-114;8.40e-03,72,212-283)	Fibronectin type III(SSF49265,8.3E-14,37-120;1.9E-10,120-213;2.8E-6,212-304)
147	105,118	106,791	1,674	557								Immunoglobulin-like fold(G3DSA:2.60.40.10,7.6E-10,33-109;2.8E-6,121-213;2.3E-5,214-285)
148	106,866	107,126	261	86								signal-peptide(SignalP-NN(euk),-1.0,1-28)
												transmembrane_regions(tmhmm,-1.0,4-19)
149	107,148	107,774	627	208	hypotheical protein OtV2_075	Ostreococcus tauri virus 2	YP_004063508	67.30%	9.21E-87	191(3-193)	PRK13435(PRK13435,8.30e-03,38,30-67)	
					EsV-1-137	Ectocarpus	CBN80435	27.00%	2.00E-03			

ID	Position		Length		BlastP hit in GenBank nr and/or viruses data set						NCBI conserved domain (ID, E-value, alignment length in aa, alignment start - end position)	Interproscan matches (ID, E-value, alignment start - end position)
	start	end	nt	aa	ORF, protein encoded, or mass	Species	Accession	Aa identity	E Value	Alignment length in aa (Start-end position)		
						siliculosus						
150	107,811	108,482	672	223	hypotheical proein OlV1_090	Ostreococcus lucimarinus virus OlV1	YP_004061723	42.20%	4.67E-50	223(1-223)	AdoMet_MTases(cd02440,3.91e-10,101,26-126)	Pox_MCEL(PF03291,3.8E-12,3-68;2.0E-4,87-152)
					hypotheical proein Ecym_2270	Eremohecium cymbalariae DBVPG#7215	XP_003644833	32.80%	3.09E-18	200(3-202)		S-adenosyl-L-methionine-dependent methyltransferases(SSF53335,3.1E-20,17-221)
151	108,481	108,912	432	143	hypothetical protein MPXG_00018	Micromonas pusilla virus SP1	AET84816	39.00%	5.61E-26	138(4-141)	WLM(pfam08325,7.65e-06,58,68-125)	WLM(PF08325,5.3E-8,66-126)
												signal-peptide(SignalP-NN(euk),-1.0,1-20)
												transmembrane_regions(tmhmm,-1.0,5-20)
152	108,909	109,409	501	166	hypothetical protein BpV1_077c	Bahycoccus sp. RCC1105 virus BpV1	YP_004061507	39.00%	4.75E-20	138(25-162)		transmembrane_regions(tmhmm,-1.0,104-122;-1.0,141-159)
153	109,450	110,235	786	261	VV A32 virion packaging ATPase	Ostreococcus tauri virus 2	YP_004063517	62.10%	1.55E-128	261(1-261)	AAA(smart00382,1.60e-03,122,17-138)	no descripion(G3DSA:3.40.50.300,2.0E-4,17-140)
154	110,268	113,168	2,901	966	hypothetical protein OlV1_097	Ostreococcus lucimarinus virus OlV1	YP_004061730	31.60%	3.96E-44	323(643-965)	Capsid_NCLDV(pfam04451,1.50e-26,177,785-961)	Capsid_NCLDV(PF04451,2.2E-30,785-960)
												Adenovirus Pll,hexon,subdomain 4(G3DSA:2.70.9.10,6.5E-23,652-805)
												Group II dsDNA viruses VP(SSF49749,5.8E-33,38-805; 1.7E-25,806-964)
												African swine fever virus, p72,major capsid(G3DSA:2.70.9.20,2.2E-28,809-964)

ID	Position		Length		BlastP hit in GenBank nr and/or viruses data set						NCBI conserved domain (ID, E-value, alignment length in aa, alignment start - end position)	Interproscan matches (ID, E-value, alignment start - end position)
	start	end	nt	aa	ORF, protein encoded, or mass	Species	Accession	Aa identity	E Value	Alignment length in aa (Start-end position)		
155	113,184	114,314	1,131	376	hypothetical protein MPXG_00023	Micromonas pusilla virus SP1	AET84821	31.00%	1.74E-39	357(1-357)	Capsid_NCLDV(pfam04451,7.85e-21,166,191-356)	signal-peptide(SignalP-NN(euk),-1.0,1-21) Capsid_NCLDV(PF04451,5.6E-24,212-356)
					EsV-1-116	Ectocarpus siliculosus	CBN80416	24.00%	9.40E+00			Adenovirus Pll,hexon,subdomain 4(G3DSA:2.70.9.10,1.2E-17,43-155)
												Group II dsDNA viruses VP(SSF49749,3.3E-22,24-211; 6.8E-21,206-365)
												African swine fever virus, p72,major capsid(G3DSA:2.70.9.20,1.4E-24,213-357)
156	114,350	114,721	372	123	hypothetical protein OV2_087	Ostreococcus tauri virus 2	YP_004063520	47.00%	1.61E-31	114(6-119)		
157	114,722	114,892	171	56								
158	114,894	115,178	285	94	hypothetical protein MPXG_00025	Micromonas pusilla virus SP1	AET84823	54.70%	5.39E-26	86(8-93)		transmembrane_regions(tmhmm,-1.0,21-41;-1.0,51-71)
159	115,175	116,185	1,011	336	hypothetical protein ATCVOR07043_989 R	Acanthocystis turfacea Chlorella virus OR0704.3	AGE59592	23.80%	2.37E-14	284(1-284)		transmembrane_regions(tmhmm,-1.0,304-324)
160	116,105	117,286	1,182	393	2OG-Fe(II) oxygenase	Prochlorococcus phage P-RSM4	YP_004323166	40.20%	2.32E-36	185(208-392)	2OG-FeII_Oxy_3(pfam13640, 1.68e-07,77,314-390)	2OG-FeII_Oxy_3(PF13640,5.2E-9,314-390)
					puaive hydroxylase	Maricaulis maris MCS10	YP_756905	21.90%	6.58E-05	133(259-391)		FE2OG_OXY(PS51471,10.828,308-392)
161	117,261	119,087	1,827	608								UNCHARACTERIZED(PHR13029,3.4E-9,482-606)
											Peptidase_S74(pfam13884,3.64e-10,57,502-558)	Peptidase_S74(PF13884,4.5E-12,502-558)

ID	Position		Length		BlastP hit in GenBank nr and/or viruses data set						NCBI conserved domain (ID, E-value, alignment length in aa, alignment start - end position)	Interproscan matches (ID, E-value, alignment start - end position)
	start	end	nt	aa	ORF, protein encoded, or mass	Species	Accession	Aa identity	E Value	Alignment length in aa (Start-end position)		
162	119,098	121,914	2,817	938	Chlorovirus glycoprotein repeat domain-containing protein	Acanthocystis turfacea Chlorella virus GM0701.1	AGE53651	29.00%	3.55E-10	364(137-500)		signal-peptide(SignalP-NN(euk),-1.0,1-15)
163	121,953	122,639	687	228							Laminin_G_3(pfam13385,2.11e-08,88,56-143)	Concanavalin A-like lectins/glucanases(SSF49899,2.5E-14,33-185)<br>Concanavalin A-like lectin/glucanase, subgroup(G3DSA:2.60.120.200,6.2E-10,44-183)<br>Laminin_G_3(PF13385,3.0E-12,47-181)
164	122,636	124,261	1,626	541	LamG domain-conaining protein	Cyclobacerium marinum DSM 745	YP_004772733	31.00%	1.04E-08	180(43-222)	Laminin_G_3(pfam13385,6.66e-21,153,66-218)	LamG-like jellyroll fold domain(SM00560,0.0054,76-215)
					hypothetical protein OSG_eHP7_00185, parial	Environmenal Halophage eHP-7	AFH21718	38.40%	1.42E-08	111(245-355)		Concanavalin A-like lectins/glucanases(SSF49899,1.3E-27,14-224;0.0016,322-535)<br>Concanavalin A-like lecin/glucanase, subgroup(G3DSA:2.60.120.200,4.4E-27 ,15-223)
165	124,290	125,807	1,518	505	hypothetical protein ACD_8C00080G0006	unculured bacerium	EKE19944	44.60%	1.40E-05	198(45-242)	Laminin_G_3(pfam13385,3.40e-09,118,330-447)	signal-pepide(SignalP-NN(euk),-1.0,1-17)<br>Concanavalin A-like lectins/glucanases(SSF49899,2.1E-15,299-483)<br>Concanavalin A-like lectin/glucanase, subgroup(G3DSA:2.60.120.200,1.2E-15,297-482)<br>Laminin_G_3(PF13385,4.7E-12,331-477)

ID	Position		Length		BlastP hit in GenBank nr and/or viruses data set						NCBI conserved domain (ID, E-value, alignment length in aa, alignment start - end position)	Interproscan matches (ID, E-value, alignment start - end position)
	start	end	nt	aa	ORF, protein encoded, or mass	Species	Accession	Aa identity	E Value	Alignment length in aa (Start-end position)		
166	125,812	126,075	264	87	Ervl/Alr family protein	Lymphocysis disease virus - isolae China	YP_073647	34.00%	2.70E-08	83(2-84)	Evr1_Alr(pfam04777,1.69e-13,75,5-79)	Evr1_Alr(PF04777,7.2E-12,8-78)
					FAD-linked sulfhydryl oxidase	Galdieria sulphuraria	EME26178	29.00%	1.00E-04			FAD-dependent thiol oxidase(SSF69000,1.0E-12,2-87)<br>ERV_ALR(PS51324,15.999,1-84)<br>signal-peptide(SignalP-NN(euk),-1.0,1-22)
167	126,084	127,895	1,812	603	PREDICTED: uncharacterized protein LOC778550	Ciona intestinalis	XP_002124516	39.60%	2.07E-08	140(344-483)		
168	127,939	129,717	1,779	592	hypothetical protein BRAFLDRAF_119089	Branchiosoma floridae	XP_002590999	49.20%	1.16E-15	128(311-438)		
169	129,714	134,648	4,935	1644	Chlorovirus glycoprotein repeat domain-containing protein	Acanthocystis turfacea Chlorella virus GM0701.1	AGE53649	24.80%	7.04E-10	649(389-1037)		Parallel beat-helix repeats(SM00710,6300.0,18-43;6600.0,57-82;3000.0,101-125;8700.0,199-220<br>4300.0 222-246<br>3000.0 257-282<br>6200.0 332-357<br>7100.0 385-407<br>2600.0 437-459<br>6300.0 476-501<br>6900.0 528-550<br>3800.0 606-631<br>3200.0 645-667<br>5700.0 702-724<br>7600.0 789-811<br>6500.0 841-863<br>1700.0 911-937

ID	Position		Length		BlastP hit in GenBank nr and/or viruses data set						NCBI conserved domain (ID, E-value, alignment length in aa, alignment start - end position)	Interproscan matches (ID, E-value, alignment start - end position)
	start	end	nt	aa	ORF, protein encoded, or mass	Species	Accession	Aa identity	E Value	Alignment length in aa (Start-end position)		
												3900.0 977-1010<br>820.0 1247-1276
170	134,698	134,937	240	79								
171	135,057	135,242	186	61								
172	135,325	135,876	552	183								
173	135,886	136,470	585	194	hypothetical protein MpV1_069	Micromonas sp. RCC1109 virus MpV1	YP_004061952	29.90%	9.99E-08	121(20-140)		transmembrane_regions(tmhmm,-1.0,171-189)
174	136,471	137,040	570	189	hypothetical protein MPVG_00059	Micromonas pusilla virus 12T	YP_007676127	32.40%	2.94E-15	179(8-186)		
175	137,061	137,378	318	105	hypothetical protein OlV1_071	Ostreococcus lucimarinus virus OlV1	YP_004061704	43.80%	3.71E-16	96(8-103)		signal-peptide(SignalP-NN(euk),-1.0,1-27)<br>transmembrane_regions(tmhmm,-1.0,7-25)

ID	Position		Length		BlastP hit in GenBank nr and/or viruses data set						NCBI conserved domain (ID, E-value, alignment length in aa, alignment start - end position)	Interproscan matches (ID, E-value, alignment start - end position)
	start	end	nt	aa	ORF, protein encoded, or mass	Species	Accession	Aa identity	E Value	Alignment length in aa (Start-end position)		
176	137,454	138,530	1,077	358	hypothetical protein MPWG_00261	Micromonas pusilla virus PL1	AET43745	49.20%	3.02E-93	340(2-341)		
					hypothetical protein VOLCADRAFT_99759	Volvox carteri f. nagariensis	XP_002958510	48.30%	2.22E-35	142(119-260)		
177	138,551	138,727	177	58	hypothetical protein MPVG_00056	Micromonas pusilla virus 12T	YP_007676124	46.40%	1.06E-07	54(3-56)		transmembrane_regions(tmhmm,-1.0,36-56)
178	138,724	140,091	1,368	455	hypothetical protein BpV1_050	Bathycoccus sp. RCC1105 virus BpV1	YP_004061480	54.50%	7.10E-162	441(4-444)	DEXDc(cd00046,7.19e-11,123,108-230)	HELICASE_CTER(PS51194,6.68,301-447)
					EsV-1-66	Ectocarpus siliculosus	CBN80369	34.00%	1.00E-65		HELICc(cd00079,1.79e-07,105,298-402)	HELICASE_ATP_BIND_1(PS51192,17.362,100-248)
												P-loop containing nucleoside triphosphate hydrolases(SSF52540,3.1E-34,92-315;4.0E-16,105-443)
179	140,075	140,353	279	92	hypothetical protein MpV1_062	Micromonas sp. RCC1109 virus MpV1	YP_004061945	28.60%	8.38E-06	69(1-69)		
180	140,362	141,735	1,374	457	ESX-1 secretion associated protein EspK, Alanine and Proline rich	Mycobacterium canettii CIPT 140070010	YP_007266598	69.80%	1.24E-06	49(370-418)		transmembrane_regions(tmhmm,-1.0,436-456)
181	141,715	142,584	870	289	hypothetical protein BpV2_045	Bathycoccus sp. RCC1105 virus BpV2	ADQ91212	24.80%	7.70E-04	218(48-265)		transmembrane_regions(tmhmm,-1.0,248-266)
182	142,621	142,803	183	60								signal-peptide(SignalP-NN(euk),-1.0,1-22)
												transmembrane_regions(tmhmm,-1.0,5-25)

ID	Position		Length		BlastP hit in GenBank nr and/or viruses data set						NCBI conserved domain (ID, E-value, alignment length in aa, alignment start - end position)	Interproscan matches (ID, E-value, alignment start - end position)
	start	end	nt	aa	ORF, protein encoded, or mass	Species	Accession	Aa identity	E Value	Alignment length in aa (Start-end position)		
183	142,800	143,579	780	259	FAD-dependent thymidylate synthase	Micromonas pusilla virus SP1	AET85027	56.30%	3.63E-91	253(4-256)	thyX(PRK00847,1.79e-89,210,1-210)	ThyX(MF_01408,28.21,1-215)
					thymidylate synthase	Dictyostelium purpureum	XP_003285756	52.40%	6.37E-88	257(2-258)		
184	143,607	144,269	663	220								signal-peptide(SignalP-NN(euk),-1.0,1-42)
												transmembrane_regions(tmhmm,-1.0,28-50)
185	144,269	144,955	687	228	hypothetical protein MPVG_00042	Micromonas pusilla virus 12T	YP_007676110	56.10%	2.12E-86	227(1-227)	Pox_A22(pfam04848,4.39e-06,158,2-159)	Pox_A22(PF04848,6.1E-13,2-159)
					hypothetical protein CAOG_03684	Capsaspora owczarzaki ATCC 30864	EFW45700	26.00%	2.20E-01			Ribonuclease H-like(SSF53098 ,4.1E-14,2-160)
186	144,915	145,487	573	190	hypothetical protein OtV6_048c	Ostreococcus tauri virus RT-2011	AFC34956	39.60%	1.09E-32	155(31-185)		zf-FCS(PF06467,9.3E-7,26-72)
					EsV-1-96	Ectocarpus siliculosus	CBN80398	34.00%	8.00E-06			signal-peptide(SignalP-NN(euk),-1.0,1-37)
												transmembrane_regions(tmhmm,-1.0,15-35)
187	145,484	145,807	324	107								
188	145,800	146,039	240	79	hypothetical protein OtV2_041	Ostreococcus tauri virus 2	YP_004063474	52.60%	4.05E-17	75(3-77)		
					hypothetical protein VOLCADRAFT_105974	Volvox carteri f. nagariensis	XP_002953453	36.00%	1.14E-05	71(7-77)		
189	146,103	146,270	168	55								
190	146,233	146,880	648	215	transcription elongation factor SII	Micromonas pusilla virus 12T	YP_007676093	39.50%	3.37E-38	185(31-215)	TFIIS_C(pfam01096,1.79e-17,39,176-214)	C2C2 Zinc finger(SM00440,2.2E-18,176-215)
					hypothetical protein COCSUDRAFT_256	Coccomyxa subellipsoidea C-	EIE19640	44.30%	3.14E-17	97(118-214)		TRANSCRIPTION ELONGATION FACTOR S-II(PTHR11477,4.6E-19,172-

ID	Position		Length		BlastP hit in GenBank nr and/or viruses data set						NCBI conserved domain (ID, E-value, alignment length in aa, alignment start - end position)	Interproscan matches (ID, E-value, alignment start - end position)
	start	end	nt	aa	ORF, protein encoded, or mass	Species	Accession	Aa identity	E Value	Alignment length in aa (Start-end position)		
					84	169						214)
												Zinc beta-ribbon(SSF57783,7.2E-16,153-214)
191	146,861	147,748	888	295	capsular polysaccharide synthesis	Acidovorax avenae subsp. avenae ATCC 19860	YP_004232817	31.10%	1.40E-37	270(24-293)	Gly_transf_sug(pfam04488,5.76e-04,80,44-123)	Caps_synth(PF05704,2.8E-27,21-157)
192	147,768	148,403	636	211	Protein of unknown function (DUF3431)	Desulfovibrio sp. U5L	ZP_10077131	36.10%	7.47E-03	72(25-96)	DUF3431(pfam11913,2.32e-04,38,64-101)	signal-peptide(SignalP-NN(euk),-1.0,1-24)<br>signal-peptide(SignalP-NN(euk),-1.0,1-19)<br>transmembrane_regions(tmhmm,-1.0,5-20)
193	148,445	148,861	417	138								
194	149,014	149,295	282	93	hypothetical protein BpV1_020c	Bathycoccus sp. RCC1105 virus BpV1	YP_004061450	36.50%	2.70E-12	83(7-89)		
195	149,261	149,725	465	154								
196	149,827	150,381	555	184	hypothetical protein MPXG_00183	Micromonas pusilla virus SP1	AET84981	38.10%	6.98E-14	97(2-98)	RING(cd00162,8.64e-07,44,4-47)	Zinc finger, RING/FYVE/PHD-type(G3DSA:3.30.40.10,4.7E-11,3-55)
					hypothetical protein COCSUDRAFT_67348	Coccomyxa subellipsoidea C-169	EIE20408	42.90%	1.92E-06	52(3-54)		PEROXISOME ASSEMBLY PROTEIN 10(PTHR23350,1.2E-4,1-48)
197	150,399	150,854	456	151	hypothetical protein OlV1_201	Ostreococcus lucimarinus virus OlV1	YP_004061833	41.90%	1.86E-14	115(15-129)		
					hypothetical protein SSON53_00120	Shigella sonnei 53G	YP_005454613	32.20%	1.34E-02	82(23-104)		
198	150,829	151,323	495	164	hypothetical protein CHLNCDRAFT_298	Chlorella variabilis	EFN58861	26.10%	2.94E-07	152(5-156)		Aspartate decarboxylase-like domain(G3DSA:2.40.40.20,4.4E-9,3-72)

ID	Position		Length		BlastP hit in GenBank nr and/or viruses data set						NCBI conserved domain (ID, E-value, alignment length in aa, alignment start - end position)	Interproscan matches (ID, E-value, alignment start - end position)
	start	end	nt	aa	ORF, protein encoded, or mass	Species	Accession	Aa identity	E Value	Alignment length in aa (Start-end position)		
					82							
199	151,354	152,019	666	221								
200	151,957	152,376	420	139	hypothetical protein BB_N41	Borrelia burgdorferi B31	NP_051452	35.30%	9.54E-02	51(67-117)		
201	152,351	152,722	372	123								
202	152,747	153,151	405	134								
203	153,175	154,995	1,821	606	PBCV-specific basic adaptor domain-containing protein	Acanthocystis turfacea Chlorella virus Canal-1	AGE50033	28.10%	6.21E-12	267(5-271)		
204	155,001	155,333	333	110								
205	155,354	155,614	261	86								
206	155,733	156,143	411	136								
207	156,362	156,598	237	78								
208	156,623	156,862	240	79								
209	156,887	157,078	192	63								
210	157,112	157,495	384	127	hypothetical protein Oscil6304_6037	Oscillatoria acuminata PCC 6304	YP_007100729	27.00%	7.00E-01	84(39-122)		
211	157,520	157,900	381	126	hypothetical protein PGUG_01549	Meyerozyma guilliermondii ATCC 6260	EDK37451	32.60%	1.08E-02	84(30-113)	Acetyltransf_7(pfam13508,8.70e-08,70,22-91)	Acyl-CoA N-acyltransferase(G3DSA:3.40.630.30,1.1E-5,23-87)
212	158,077	158,484	408	135	N-acetyltransferase GCN5	Sebaldella termitidis ATCC 33386	YP_003310592	32.60%	2.88E-03	87(41-127)	Acetyltransf_7(pfam13508,1.41e-04 ,77,49-125)	Acyl-CoA N-acyltransferase(G3DSA:3.40.630.30,1.1E-5,44-129)
213	158,489	158,656	168	55								
214	158,664	158,879	216	71								
215	158,879	159,058	180	59								
216	159,091	159,672	582	193	C3HC4 finger protein	Aspergillus flavus NRRL3357	XP_002378950	41.30%	1.05E-04	59(133-191)	ThylakoidFormat Superfamily(cl12138,4.73e-	Zinc finger, RING/FYVE/PHD-type(G3DSA:3.30.40.10,4.7E-10,132-186)

ID	Position		Length		BlastP hit in GenBank nr and/or viruses data set						NCBI conserved domain (ID, E-value, alignment length in aa, alignment start - end position)	Interproscan matches (ID, E-value, alignment start - end position)
	start	end	nt	aa	ORF, protein encoded, or mass	Species	Accession	Aa identity	E Value	Alignment length in aa (Start-end position)		
											03,37,84-120)	
217	159,664	159,999	336	111	protein ORF144	Cyprinid herpesvirus 2	YP_007003959	41.30%	3.58E-04	46(134-179)	RING(cd00162,7.19e-06,47,137-183)	RING/U-box(SSF57850,5.4E-10,122-186)
					Chain B, Structure Of A Beta-Trcp1-Skp1-Beta-Catenin Complex: Destruction Motif Binding And Lysine Specificity On The Scfbeta-Trcp1 Ubiquitin Ligase	Homo sapiens	1P22_B	31.10%	1.22E-11	111(1-111)	Skp1(smart00512,1.23e-09,80,3-82)	SKP1(PTHR11165,7.0E-12,3-111)
					hypothetical protein FR483_N799R	Paramecium bursaria Chlorella virus FR483	YP_001426431	33.10%	2.47E-09	111(1-111)		BTB/POZ fold(G3DSA:3.30.710.10,7.8E-14,2-111)
218	160,039	160,383	345	114	multidrug resistance protein MdtJ	Yersinia pseudotuberculosis IP 31758	YP_001400993	33.30%	5.88E-04	102(13-114)		transmembrane_regions(tmhmm,-1.0,42-62;-1.0,68-88)
												Multidrug resistance efflux transporter EmrE(SSF103481,6.5E-6,13-113)
219	160,422	160,718	297	98								
220	160,724	160,891	168	55								signal-peptide(SignalP-NN(euk),-1.0,1-39)
												transmembrane_regions(tmhmm,1.0,21-39)
221	161,057	161,791	735	244	hypothetical protein OLOG_00261	Ostreococcus lucimarinus virus OlV4	AET84716	42.20%	1.65E-53	234(1-234)	phage_rel_nuc(TIGR03033,5.29e-03,59,2-60)	Exonuclease, phage-type/RecB, C-terminal(G3DSA:3.90.320.10, 1.0E-9,98-239)
											PDDEXK_1(pfam12705,8.05e-03,59,138-196)	YqaJ(PF09588,5.5E-5,2-199)

ID	Position		Length		BlastP hit in GenBank nr and/or viruses data set						NCBI conserved domain (ID, E-value, alignment length in aa, alignment start - end position)	Interproscan matches (ID, E-value, alignment start - end position)
	start	end	nt	aa	ORF, protein encoded, or mass	Species	Accession	Aa identity	E Value	Alignment length in aa (Start-end position)		
222	161,826	162,107	282	93	CP12 domain protein	Coleofasciculus chthonoplastes	WP_006106059	32.30%	5.50E-02	90(3-92)	CP12(pfam02672,5.22e-03,42,31-72)	CP12(PF02672,2.8E-6,31-73)
223	162,167	162,820	654	217								
224	162,854	163,072	219	72								signal-peptide(SignalP-NN(euk),-1.0,1-20)
225	163,085	163,360	276	91								
226	163,382	164,053	672	223	hypothetical protein MPXG_00109	Micromonas pusilla virus SP1	AET84907	24.00%	1.70E-09	152(16-167)		transmembrane_regions(tmhmm,-1.0,52-70;-1.0,80-100)
227	164,046	164,384	339	112								signal-peptide(SignalP-NN(euk),-1.0,1-28)
228	164,393	164,896	504	167								
229	164,930	165,460	531	176	putative ring zinc finger protein, partial	Cucumis sativus	AEP25855	41.20%	4.83E-06	65(112-176)	RING(cd00162,1.35e-04,50,125-174)	Zinc finger, RING/FYVE/PHD-type(G3DSA:3.30.40.10,2.7E-12,123-175)<br>RING/U-box(SSF57850,8.8E-14,93-175)
230	165,483	165,710	228	75								
231	165,711	165,983	273	90								
232	165,970	166,917	948	315	conserved hypothetical protein	Albugo laibachii Nc14	CCA21495	35.60%	2.25E-47	305(2-306)	DnaJ_bact(TIGR02349,2.40e-70,308,2-309)	DnaJ/Hsp40 cysteine-rich domain(SSF57938,3.8E-6,114-185)<br>Chaperone J-domain(SSF46565,1.6E-26,1-106)<br>HSP40/DnaJ peptide-binding domain(SSF49493,3.2E-12,92-232;7.4E-9,233-305)
233	166,942	167,376	435	144								
234	167,385	169,250	1,866	621								
235	169,311	169,934	624	207	endonuclease HhaII	Paramecium bursaria Chlorella virus CVB-1	AGE50806	33.30%	1.23E-33	203(1-203)		
					hypothetical protein cco10_08933	Campylobacter coli 90-3	ZP_14078815	28.80%	4.23E-12	203(1-203)		

ID	Position		Length		BlastP hit in GenBank nr and/or viruses data set						NCBI conserved domain (ID, E-value, alignment length in aa, alignment start - end position)	Interproscan matches (ID, E-value, alignment start - end position)
	start	end	nt	aa	ORF, protein encoded, or mass	Species	Accession	Aa identity	E Value	Alignment length in aa (Start-end position)		
236	169,911	170,975	1,065	354	site-specific DNA-methyltransferase CviBI	Micromonas pusilla virus 12T	YP_007676256	56.00%	2.19E-103	259(95-353)	dam(TIGR00571,9.03e-69,258,95-352)	dam: DNA adenine methylase(TIGR00571,4.7E-68,95-352)
					N.BstNBI methyltransferase	Geobacillus stearothermophilus	AAK08495	38.90%	3.02E-53	259(95-353)		S-adenosyl-L-methionine-dependent methyltransferases(SSF53335,1.8E-50,94-353)
												signal-peptide(SignalP-NN(euk),-1.0,1-21)
237	170,866	171,573	708	235								
238	171,740	171,925	186	61								
239	171,988	172,296	309	102	dihydrodipicolinate synthetase	Lentibacillus sp. Grbi	ZP_09918746	33.80%	4.84E-02	60(18-77)		
240	172,293	173,111	819	272	hypothetical protein SFHH103_00274	Sinorhizobium fredii HH103	YP_005187601	33.20%	2.92E-18	182(73-254)		
					gp15	Mycobacterium phage Thibault	AEJ93965	29.20%	5.32E-10	133(2-134)		
241	173,145	173,837	693	230	hypothetical protein NAEGRDRAFT_59766	Naegleria gruberi	XP_002670181	43.30%	2.59E-48	211(20-230)	Glyco_hydro_cc（pfam11790，1.59e-48，180，48-227）	Glyco_hydro_cc(PF11790 ,1.9E-52,10-227)
242	173,781	174,059	279	92	248R	Invertebrate iridescent virus 6	NP_149711	35.40%	6.09E-08	75(1-75)		signal-peptide(SignalP-NN(euk),-1.0,1-44)
												transmembrane_regions(tmhmm,-1.0,21-43;-1.0,49-67)
243	174,094	174,714	621	206								
244	174,683	175,435	753	250								
245	175,465	176,760	1,296	431	DNA ligase	Vibrio phage KVP40	NP_899305	37.40%	2.09E-70	427(5-431)	DNA_ligase_A_M（pfam01068，2.28e-20，194，142-335）	DNA_ligase_A_M(PF01068,5.1E-22,146-335)
					hypothetical protein Mpe_B0266	Methylibium petroleiphilum PM1	YP_001023276	35.00%	1.84E-46	427(5-431)		Nucleic acid-binding proteins(SSF50249,3.3E-15,308-422)

ID	Position		Length		BlastP hit in GenBank nr and/or viruses data set						NCBI conserved domain (ID, E-value, alignment length in aa, alignment start - end position)	Interproscan matches (ID, E-value, alignment start - end position)
	start	end	nt	aa	ORF, protein encoded, or mass	Species	Accession	Aa identity	E Value	Alignment length in aa (Start-end position)		
246	176,819	177,091	273	90	hypothetical protein DICPUDRAFT_155857	Dictyostelium purpureum	XP_003291267	39.00%	1.54E-07	49(2-50)	zf-C3HC4_2（pfam13923，3.33e-03，44,2-45）	DNA ligase/mRNA capping enzyme, catalytic domain(SSF56091,2.6E-31,135-335)<br>zf-RING_2(PF13639,7.9E-11,2-45)
					hypothetical protein EXVG_00190	Emiliania huxleyi virus 202	AET42539	37.50%	5.53E-06	63(3-65)		RING FINGER PROTEIN 24-RELATED(PTHR22766,2.4E-8,19-62)<br>RING/U-box(SSF57850,5.3E-11,3-50)
247	177,080	177,313	234	77								
248	177,328	177,870	543	180								
YSLPV2												
1	514	663	150	49								signal-peptide(SignalP-NN(euk),-1.0,1-36)
2	884	2,635	1752	583	tail fiber-like protein	Chromobacterium violaceum ATCC 12472	NP_900088	25.30%	4.29E-06	355(228-582)		
3	2,809	2,970	162	53								
4	3,056	7,192	4137	1378	putative low-complexity protein	Leptolyngbya sp. PCC 7375	WP_006512875.1	28.00%	3.00E-26	519(236-754)		
5	7,221	9,839	2619	872	hypothetical protein SXBG_00214	Synechococcus phage S-CAM1	YP_007673127	45.80%	3.15E-145	651(175-825)		
					hypothetical protein Dred_1404	Desulfotomaculum reducens MI-1	YP_001112759	34.90%	3.45E-04	85(494-578)		
6	9,830	10,435	606	201								
7	10,626	10,808	183	60							SKN1(pfam03935,9.42e-03,35,7-41)	

ID	Position		Length		BlastP hit in GenBank nr and/or viruses data set						NCBI conserved domain (ID, E-value, alignment length in aa, alignment start - end position)	Interproscan matches (ID, E-value, alignment start - end position)
	start	end	nt	aa	ORF, protein encoded, or mass	Species	Accession	Aa identity	E Value	Alignment length in aa (Start-end position)		
8	10,831	15,501	4671	1556	virion structural protein	Cyanophage S-TIM5	YP_007006099.1	30.00%	6.00E-31	452(1103-1554)	COG1357(COG1357,5.33e-05,169,286-454)	Peptidase_S74(PF13884,1.1E-8,1445-1502)
					YapH protein	Bdellovibrio bacteriovorus HD100	NP_968058.1	37.00%	3.00E-07	101(1441-1541)		
9	15,502	15,954	453	150								signal-peptide(SignalP-NN(euk),-1.0,1-16)
10	16,194	16,856	663	220								
11	16,856	17,419	564	187	hypothetical protein	Pseudanabaena sp. PCC 6802	WP_019500721	31.10%	1.43E-12	178(5-182)	2OG-FeII_Oxy_5(cl17879,1.42e-20,94,89-182)	TIGR02466: conserved hypothetical protein(TIGR02466,1.8E-21,4-183)
12	17,420	17,731	312	103	hypothetical protein CYXG_00205	Synechococcus phage S-SSM4	YP_007677394	31.10%	3.80E-11	127(57-183)		2OG-FeII_Oxy_5(PF13759,1.1E-20,89-182)
13	17,685	18,758	1074	357	2OG-Fe(II) oxygenase	Prochlorococcus phage P-RSM4	YP_004323166	34.70%	1.67E-21	175(7-181)	2OG-FeII_Oxy_3 super family(cl17304,1.10e-07,157,23-179;3.28e-04,81,256-336)	2OG-FeII_Oxy_3(PF13640,7.2E-8,103-179;3.8E-6,256-334)
					prolyl 4-hydroxylase subunit alpha	Candidatus Solibacter usitatus Ellin6076	YP_827606	34.00%	7.11E-06	101(252-352)		Prolyl 4-hydroxylase alpha subunit homologue(SM00702,1.9E-4,10-180)

ID	Position		Length		BlastP hit in GenBank nr and/or viruses data set						NCBI conserved domain (ID, E-value, alignment length in aa, alignment start - end position)	Interproscan matches (ID, E-value, alignment start - end position)
	start	end	nt	aa	ORF, protein encoded, or mass	Species	Accession	Aa identity	E Value	Alignment length in aa (Start-end position)		
14	18,786	19,643	858	285								
			1155	384	hypothetical protein	Verrucomicrobia bacterium SCGC AAA164-P11	WP_020036727	40.60%	2.91E-32	173(212-384)	2OG-FeII_Oxy_3(cl17304,9.81e-14,87,292-378)	2OG-FeII_Oxy_3(PF13640,4.1E-9,301-378)
15	19,574	20,728										
												Prolyl 4-hydroxylase alpha subunit homologue(SM00702,7.9E-5,209-379)
16	20,670	21,674	1005	334	transcription factor jumonji jmjC domain-containing protein	Chroococcidiopsis thermalis PCC 7203	YP_007094572	44.10%	5.86E-04	57(12-68)	Cupin_8 super family(cl18657,4.91e-08,45,24-68)	Cupin_8 （PF13621,2.1E-9,20-68;1.5E-9,156-206)
			579	192	prolyl 4-hydroxylase	Synechococcus phage S-CAM1	YP_007672989	43.10%	2.69E-39	182(1-182)		2OG-FeII_Oxy_3(PF13640,2.8E-7,92-180)
17	21,680	22,258			hypothetical protein EMIHUDRAFT_455045	Emiliania huxleyi CCMP1516	EOD36489	40.30%	6.63E-39	180(3-182)	2OG-FeII_Oxy_3 Superfamily(cl17304,1.62e-10,169,12-180)	PROLYL 4-HYDROXYLASE ALPHA SUBUNIT(PTHR10869,1.1E-6,90-155)
18	22,273	23,304	1032	343	hypothetical protein ATCVOR07043_989R	Acanthocystis turfacea Chlorella virus OR0704.3	AGE59592	26.70%	1.64E-06	205(1-205)		transmembrane_regions(tmhmm,-1.0,303-323)
19	23,349	23,630	282	93	hypothetical protein MPXG_00025	Micromonas pusilla virus SP1	AET84823	58.10%	3.04E-18	86(8-93)		

ID	Position		Length		BlastP hit in GenBank nr and/or viruses data set						NCBI conserved domain (ID, E-value, alignment length in aa, alignment start - end position)	Interproscan matches (ID, E-value, alignment start - end position)
	start	end	nt	aa	ORF, protein encoded, or mass	Species	Accession	Aa identity	E Value	Alignment length in aa (Start-end position)		
					hypothetical protein	Verrucomicrobia bacterium SCGC AAA164-N20	WP_020036346	59.80%	5.36E-16	92(2-93)		transmembrane_regions(tmhmm,-1.0,15-33;52-72)
20	23,634	23,801	168	55								
			492	163								signal-peptide(SignalP-NN(euk),-1.0,1-18)
21	23,810	24,301										
												transmembrane_regions(tmhmm,-1.0,75-95)
					hypothetical protein OlV1_097	Ostreococcus lucimarinus virus OlV1	YP_004061730	30.20%	1.83E-39	372(1-372)	Capsid_NCLDV(pfam04451,2.86e-26,179,191-369)	Capsid_NCLDV(PF04451,7.3E-26,191-369)
					hypothetical protein	Verrucomicrobia bacterium SCGC AAA164-N20	WP_020036343	29.10%	1.30E-29	369(1-369)		Group II dsDNA viruses VP(SSF49749,8.2E-22,206-377;1.9E-17,402-5761.7E-21,24-152)
22	24,278	25,450	1173	390								
												African swine fever virus(2.70.9.20,9.1E-25,212-368)
23	25,523	29,176	3654	1217	hypothetical protein MPWG_00160	Micromonas pusilla virus PL1	AET43647.1	32.00%	2.00E-46	312(905-1216)	Capsid_NCLDV(pfam04451,9.10e-26,178,1036-1213)	Capsid_NCLDV(PF04451,8.3E-30,1033-1212)

ID	Position		Length		BlastP hit in GenBank nr and/or viruses data set						NCBI conserved domain (ID, E-value, alignment length in aa, alignment start - end position)	Interproscan matches (ID, E-value, alignment start - end position)
	start	end	nt	aa	ORF, protein encoded, or mass	Species	Accession	Aa identity	E Value	Alignment length in aa (Start-end position)		
					hypothetical protein	Verrucomicrobia bacterium SCGC AAA164-N20	WP_020036344.1	28.00%	5.00E-33	298(920-1217)		Group II dsDNA viruses VP(SSF49749,1.0E-23,1057-1216;1.5E-20,916-1056;2.6E-6,93-164)
												African swine fever virus(2.70.9.20,7.1E-30,1060-1216)
												signal-peptide(SignalP-NN(euk),-1.0,1-44)
												transmembrane_regions(tmhmm,-1.0,28-46)
24	29,049	29,843	795	264	hypothetical protein MPWG_00162	Micromonas pusilla virus PL1	AET43649	65.40%	7.62E-121	240(1-240)	AAA(smart00382,3.30e-05,142,17-158)	P-loop containing nucleoside triphosphate hydrolases(SSF52540,6.4E-9,5-189)
					hypothetical protein	Verrucomicrobia bacterium SCGC AAA164-N20	WP_020036342	57.70%	6.00E-115	262(1-262)		
25	29,876	30,364	489	162	hypothetical protein BpV1_077c	Bathycoccus sp. RCC1105 virus BpV1	YP_004061507	40.30%	1.72E-16	128(31-158)		transmembrane_regions(tmhmm,-1.0,137-155)

ID	Position		Length		BlastP hit in GenBank nr and/or viruses data set						NCBI conserved domain (ID, E-value, alignment length in aa, alignment start - end position)	Interproscan matches (ID, E-value, alignment start - end position)
	start	end	nt	aa	ORF, protein encoded, or mass	Species	Accession	Aa identity	E Value	Alignment length in aa (Start-end position)		
26	30,370	30,819	450	149	hypothetical protein MPXG_00018	Micromonas pusilla virus SP1	AET84816	37.90%	9.57E-26	142(4-145)		WLM(PF08325,3.6E-8,60-136)
					hypothetical protein	Verrucomicrobia bacterium SCGC AAA164-N20	WP_020036273	35.40%	8.26E-22	124(22-145)	WLM super family(cl07077,8.81e-07,51,70-120)	signal-peptide(SignalP-NN(euk),-1.0,1-15)
27	30,825	31,706	882	293	hypothetical protein OtV6_088	Ostreococcus tauri virus RT-2011	AFC34996	43.60%	2.66E-50	220(72-291)		Methyltransf_31(PF13847,4.0E-14,92-202)
					hypothetical protein	Verrucomicrobia bacterium SCGC AAA164-N20	WP_020036272	41.90%	6.91E-45	220(72-291)	AdoMet_MTases(cd02440,4.55e-12,102,96-197)	S-adenosyl-L-methionine-dependent methyltransferases(SSF53335,9.3E-24,70-292)
28	31,580	32,197	618	205	hypothetical protein H665_p085	Ostreococcus tauri virus 1	YP_003212908	69.20%	3.18E-70	182(3-184)		
					hypothetical protein	Verrucomicrobia bacterium SCGC AAA164-N20	WP_020036266	67.00%	5.63E-65	176(1-176)		
29	32,262	32,546	285	94								signal-peptide(SignalP-NN(euk),-1.0,1-24)
												transmembrane_regions(tmhmm,-1.0,4-19)
30	33,012	33,470	459	152	hypothetical protein MPVG_00145	Micromonas pusilla virus 12T	YP_007676210	27.00%	3.37E-06	100(31-130)		signal-peptide(SignalP-NN(euk),-1.0,1-24)

ID	Position		Length		BlastP hit in GenBank nr and/or viruses data set						NCBI conserved domain (ID, E-value, alignment length in aa, alignment start - end position)	Interproscan matches (ID, E-value, alignment start - end position)
	start	end	nt	aa	ORF, protein encoded, or mass	Species	Accession	Aa identity	E Value	Alignment length in aa (Start-end position)		
31	33,322	34,389	1068	355	hypothetical protein MpV1_083	Micromonas sp. RCC1109 virus MpV1	YP_004061966	32.70%	1.56E-51	331(12-342)	Herpes_UL52 super family(cl17300,9.87e-03,35,258-292)	Herpes_UL52(PF03121,5.0E-8,251-294)
					hypothetical protein	Verrucomicrobia bacterium SCGC AAA164-N20	WP_020036261	30.10%	1.59E-39	298(16-313)		
32	34,466	34,723	258	85								
33	34,742	35,302	561	186	hypothetical protein OsV5_084f	Ostreococcus virus OsV5	YP_001648161	46.70%	8.55E-47	181(5-185)	Nudix_Hydrolase super family(cl00447,1.92e-05,56,6-61)	NUDIX(PS51462,9.104,5-169)
					hypothetical protein	Verrucomicrobia bacterium SCGC AAA164-N20	WP_020036259	43.20%	5.72E-40	181(5-185)		
34	35,452	37,182	1731	576	hypothetical protein OlV1_076	Ostreococcus lucimarinus virus OlV1	YP_004061709	56.80%	4.09E-52	175(1-175)	Capsid_NCLDV(pfam04451,5.88e-22,189,384-572)	Capsid_NCLDV(PF04451,1.3E-23,382-571)
					hypothetical protein	Verrucomicrobia bacterium SCGC AAA164-N20	WP_020036256	54.50%	1.91E-50	177(1-177)		Group II dsDNA viruses VP(SSF49749,4.2E-29,25-176;1.9E-17,402-576;0.0041,380-401)
												African swine fever virus(2.70.9.20,1.1E-21,403-576)

ID	Position		Length		BlastP hit in GenBank nr and/or viruses data set						NCBI conserved domain (ID, E-value, alignment length in aa, alignment start - end position)	Interproscan matches (ID, E-value, alignment start - end position)
	start	end	nt	aa	ORF, protein encoded, or mass	Species	Accession	Aa identity	E Value	Alignment length in aa (Start-end position)		
35	37,234	38,313	1080	359	hypothetical protein OlV1_102c	Ostreococcus lucimarinus virus OlV1	YP_004061735	32.00%	6.68E-38	348(2-349)		signal-peptide(SignalP-NN(euk),-1.0,1-20)
					hypothetical protein	Verrucomicrobia bacterium SCGC AAA164-N20	WP_020036348	30.60%	1.68E-27	346(8-353)		
36	38,390	38,662	273	90								
37	38,693	39,310	618	205	hypothetical protein MPVG_00127	Micromonas pusilla virus 12T	YP_007676193	39.90%	1.51E-39	176(2-177)		transmembrane_regions(tmhmm,-1.0,50-65)
					hypothetical protein	Verrucomicrobia bacterium SCGC AAA164-N20	WP_020036350	42.60%	3.89E-11	90(67-156)		
38	39,338	39,826	489	162	hypothetical protein BpV1_089c	Bathycoccus sp. RCC1105 virus BpV1	YP_004061519	35.00%	5.48E-18	150(2-151)		
					hypothetical protein	Verrucomicrobia bacterium SCGC AAA164-N20	WP_020036351	35.00%	5.89E-18	150(2-151)		
39	40,142	40,939	798	265	hypothetical protein BpV1_091c	Bathycoccus sp. RCC1105 virus BpV1	YP_004061521	36.40%	5.27E-30	208(1-208)		
40	40,890	41,147	258	85	hypothetical protein PBCVKS1B_593L	Paramecium bursaria Chlorella virus KS1B	AGE54713	53.80%	2.87E-06	50(12-61)		
					hypothetical protein Dalk_3573	Desulfatibacillum alkenivorans AK-01	YP_002432729	40.00%	2.89E-02	50(13-62)		
41	41,144	41,515	372	123	hypothetical protein OMVG_00158	Ostreococcus lucimarinus virus OlV3	AFK66157	47.20%	2.33E-25	123(1-123)		

ID	Position		Length		BlastP hit in GenBank nr and/or viruses data set						NCBI conserved domain (ID, E-value, alignment length in aa, alignment start - end position)	Interproscan matches (ID, E-value, alignment start - end position)
	start	end	nt	aa	ORF, protein encoded, or mass	Species	Accession	Aa identity	E Value	Alignment length in aa (Start-end position)		
42	41,522	42,190	669	222	hypothetical protein	Verrucomicrobia bacterium SCGC AAA164-N20	WP_020036584	33.60%	7.85E-17	122(1-122)		signal-peptide(SignalP-NN(euk),-1.0,1-24) transmembrane_regions(tmhmm,-1.0,5-25)
43	42,267	42,668	402	133	hypothetical protein BpV2_094	Bathycoccus sp. RCC1105 virus BpV2	ADQ91261	39.00%	1.55E-28	129(1-129)		
					hypothetical protein	Verrucomicrobia bacterium SCGC AAA164-N20	WP_020036355	36.80%	1.71E-25	129(1-129)		
44	42,695	43,438	744	247	hypothetical protein OlV1_111	Ostreococcus lucimarinus virus OlV1	YP_004061744	52.60%	1.07E-81	246(1-246)	PCNA(cl09515,4.85e-47,242,4-245)	pcna: proliferating cell nuclear antigen (pcna)（TIGR00590，2.8E-51，3-246）
					hypothetical protein	Verrucomicrobia bacterium SCGC AAA164-N20	WP_020036354	53.30%	5.40E-81	246(1-246)		
45	43,465	44,376	912	303	hypothetical protein OMVG_00153	Ostreococcus lucimarinus virus OlV3	AFK66153	51.50%	1.26E-89	293(11-303)	Pox_VLTF3(pfam04947,7.98e-56,174,129-302)	Pox_VLTF3(PF04947,3.6E-53,131-301)
46	44,588	44,938	351	116	hypothetical protein BpV1_099c	Bathycoccus sp. RCC1105 virus BpV1	YP_004061529	54.00%	6.50E-35	112(4-115)	PDDEXK_3(cl16254,5.04e-26,99,11-109)	PDDEXK_3(PF13366,4.2E-34,5-106)
					hypothetical protein	candidate division DUSEL4 archaeon SCGC AAA011-	WP_018203135	38.40%	2.07E-20	108(1-108)		

ID	Position		Length		BlastP hit in GenBank nr and/or viruses data set						NCBI conserved domain (ID, E-value, alignment length in aa, alignment start - end position)	Interproscan matches (ID, E-value, alignment start - end position)
	start	end	nt	aa	ORF, protein encoded, or mass	Species	Accession	Aa identity	E Value	Alignment length in aa (Start-end position)		
						L22						
47	44,972	45,370	399	132	hypothetical protein OtV6_119c	Ostreococcus tauri virus RT-2011	AFC35027	40.70%	5.58E-07	79(51-129)		
48	45,480	46,274	795	264	hypothetical protein MPXG_00049	Micromonas pusilla virus SP1	AET84847	38.10%	2.26E-48	257(1-257)	Patatin_and_cPLA2(cl11396,3.12e-14,131,53-183)	FabD/lysophospholipase-like(SSF52151,6.3E-21,1-198)
					Patatin	Pelosinus	WP_007935048	30.90%	8.42E-12	177(7-183)		Patatin(PF01734,5.5E-14,6-183)
49	46,382	46,762	381	126	hypothetical protein OMVG_00142	Ostreococcus lucimarinus virus OlV3	AFK66142	53.40%	4.39E-20	126(1-126)		signal-peptide(SignalP-NN(euk),-1.0,1-58)
					hypothetical protein PRUPE_ppa026813mg	Prunus persica	EMJ18658	34.40%	1.90E-04	109(10-118)		transmembrane_regions(tmhmm,-1.0,10-28;42-62;72-94;100-118)
50	46,769	47,128	360	119								
51	47,219	47,587	369	122	hypothetical protein OlV1_155	Ostreococcus lucimarinus virus OlV1	YP_004061788	40.70%	4.39E-14	113(6-118)		
					PREDICTED: uncharacterized protein C6orf72 homolog	Saimiri boliviensis boliviensis	XP_003943628	29.00%	2.85E-02	65(23-87)		
52	47,644	48,117	474	157	hypothetical protein MpV1_129c	Micromonas sp. RCC1109 virus MpV1	YP_004062012	43.80%	4.10E-24	145(4-148)		signal-peptide(SignalP-NN(euk),-1.0,1-26)

ID	Position		Length		BlastP hit in GenBank nr and/or viruses data set						NCBI conserved domain (ID, E-value, alignment length in aa, alignment start - end position)	Interproscan matches (ID, E-value, alignment start - end position)
	start	end	nt	aa	ORF, protein encoded, or mass	Species	Accession	Aa identity	E Value	Alignment length in aa (Start-end position)		
					hypothetical protein	Marine Group II euryarchaeote SCGC AB-629-J06	WP_018035657	32.60%	1.03E-05	127(28-154)		transmembrane_regions(tmhmm,-1.0,5-25)
53	48,150	48,701	552	183	hypothetical protein OLOG_00166	Ostreococcus lucimarinus virus OlV4	AET84626	37.40%	5.06E-25	174(1-174)		
54	48,737	49,132	396	131								
					hypothetical protein MPXG_00056	Micromonas pusilla virus SP1	AET84854	53.80%	1.51E-32	142(13-154)	SWIB super family(cl02489,1.82e-07,76,76-151)	SWIB/MDM2 domain(SSF47592,2.1E-13,65-153)
55	49,078	49,650	573	190								
					hypothetical protein	Bacteria	WP_018003588	28.90%	4.27E-06	131(23-153)		BRG-1 ASSOCIATED FACTOR 60 (BAF60)(PTHR13844,8.1E-6,63-155)
					hypothetical protein OtV6_130	Ostreococcus tauri virus RT-2011	AFC35038	60.50%	2.24E-88	210(14-223)	RNaseIII(TIGR02191,5.70e-66,215,19-233)	RNase_III(MF_00104,23.898,14-222)
56	49,682	50,485	804	267								dsrm(PF00035,3.1E-12,163-223)
												RNase III domain-like(SSF69065,9.9E-40,1-169)
57	50,388	51,119	732	243	hypothetical protein OlV1_148c	Ostreococcus lucimarinus virus OlV1	YP_004061781	57.80%	1.73E-69	196(1-196)	YqaJ(cl09232,1.63e-34,124,20-143)	Restriction endonuclease-like(SSF52980,1.0E-38,1-193)
												YqaJ(PF09588,9.7E-31,20-145)

ID	Position		Length		BlastP hit in GenBank nr and/or viruses data set						NCBI conserved domain (ID, E-value, alignment length in aa, alignment start - end position)	Interproscan matches (ID, E-value, alignment start - end position)
	start	end	nt	aa	ORF, protein encoded, or mass	Species	Accession	Aa identity	E Value	Alignment length in aa (Start-end position)		
58	51,046	51,240	195	64								ZF_BBOX（PS50119，9.058，1-33）
59	51,237	51,686	450	149								signal-peptide(SignalP-NN(euk),-1.0,125-139)
60	51,686	52,006	321	106	hypothetical protein OtV6_138	Ostreococcus tauri virus RT-2011	AFC35046	68.40%	2.71E-41	98(1-98)	NTP-PPase(cl16941,1.52e-10,75,10-84)	all-alpha NTP pyrophosphatases（SSF101386,3.6E-15,1-97）
					hypothetical protein	Flexithrix dorotheae	WP_020533483	30.30%	1.38E-08	99(1-99)		
61	52,032	52,259	228	75								signal-peptide(SignalP-NN(euk),-1.0,1-26) transmembrane_regions(tmhmm,-1.0,9-29;48-66)
62	52,317	52,700	384	127	hypothetical protein OtV2_125	Ostreococcus tauri virus 2	YP_004063558	40.70%	1.53E-22	123(4-126)		
63	52,742	56,029	3288	1095	hypothetical protein NY2A_B832R	Paramecium bursaria Chlorella virus NY2A	YP_001498028	42.80%	0	1095(1-1095)	RNR_PFL(cl09939,6.85e-119,263,147-409)	RIBONUCLEOSIDE-DIPHOSPHATE REDUCTASE LARGE CHAIN(PTHR11573,0.0,1-1072)
					ribonucleoside-diphosphate reductase	Gillisia sp. CBA3202	WP_010231459	57.90%	2.16E-165	409(1-409)	PLN02437(PLN02437,0,409,1-409)	

ID	Position		Length		BlastP hit in GenBank nr and/or viruses data set						NCBI conserved domain (ID, E-value, alignment length in aa, alignment start - end position)	Interproscan matches (ID, E-value, alignment start - end position)
	start	end	nt	aa	ORF, protein encoded, or mass	Species	Accession	Aa identity	E Value	Alignment length in aa (Start-end position)		
											Ribonuc_red_lgC(pfam02867,9.67e-122,314,746-1059)	
64	56,051	56,260	210	69								signal-peptide(SignalP-NN(euk),-1.0,1-18)
65	56,283	56,570	288	95	conserved hypothetical protein	Bathycoccus prasinos	CCO17397	41.50%	2.05E-06	48(3-50)	COG5219(COG5219,4.59e-06,50,2-51)	RING/U-box(SSF57850,9.9E-6,2-53)
66	56,596	57,183	588	195								transmembrane_regions(tmhmm,-1.0,89-109;124-144;158-176)
67	57,161	57,697	537	178	conserved hypothetical protein	Phytophthora infestans T30-4	XP_002906186	32.80%	9.87E-11	131(1-131)		signal-peptide(SignalP-NN(euk),-1.0,1-26)<br>transmembrane_regions(tmhmm,-1.0,12-32;79-99;143-163)
68	57,684	58,133	450	149								signal-peptide(SignalP-NN(euk),-1.0,1-29)<br>transmembrane_regions(tmhmm,-1.0,10-30;36-54;75-95;125-143)
69	58,130	58,510	381	126								

ID	Position		Length		BlastP hit in GenBank nr and/or viruses data set						NCBI conserved domain (ID, E-value, alignment length in aa, alignment start - end position)	Interproscan matches (ID, E-value, alignment start - end position)
	start	end	nt	aa	ORF, protein encoded, or mass	Species	Accession	Aa identity	E Value	Alignment length in aa (Start-end position)		
70	58,500	58,970	471	156	hypothetical protein	Acanthamoeba castellanii mamavirus	AEQ60808	54.30%	4.52E-02	35(28-62)		signal-peptide(SignalP-NN(euk),-1.0,1-16)
71	58,938	59,327	390	129								
72	59,296	59,688	393	130	hypothetical protein	Marine Group II euryarchaeote SCGC AB-629-J06	WP_018035887	48.40%	6.55E-23	93(2-94)	HNHc(cl00083,1.10e-05,51,20-70)	HNH nucleases(SM00507,0.0038,18-69)
					hypothetical protein crov535	Cafeteria roenbergensis virus BV-PW1	YP_003970168	34.10%	1.40E-05	81(2-82)		
73	59,621	60,142	522	173								
74	60,132	60,404	273	90								
75	60,376	60,723	348	115								
76	60,777	61,241	465	154	hypothetical protein	Sporichthya polymorpha	WP_019874963	41.20%	9.36E-22	133(2-134)		
77	61,559	62,194	636	211								transmembrane_regions(tmhmm,-1.0,21-41)
78	62,011	63,141	1131	376	hypothetical protein MPWG_00119	Micromonas pusilla virus PL1	AET43607	51.30%	1.31E-122	346(2-347)	DEXDc(cd00046,1.22e-16,133,28-160)	SNF2_N(PF00176,1.6E-52,10-273)

ID	Position		Length		BlastP hit in GenBank nr and/or viruses data set						NCBI conserved domain (ID, E-value, alignment length in aa, alignment start - end position)	Interproscan matches (ID, E-value, alignment start - end position)
	start	end	nt	aa	ORF, protein encoded, or mass	Species	Accession	Aa identity	E Value	Alignment length in aa (Start-end position)		
											HELICc(cd00079,1.83e-03,50,293-342)	SWI/SNF-RELATED MATRIX-ASSOCIATED ACTIN-DEPENDENT REGULATOR OF CHROMATIN SUBFAMILY-RELATED(PTHR10799,1.2E-69,9-341)
												DEAD-like helicases superfamily(SM00487,3.0E-24,2-192)
79	63,222	65,579	2358	785	hypothetical protein	Streptomyces sp. Amel2xE9	WP_019984021	51.20%	1.56E-07	84(281-364)		
80	65,588	66,382	795	264	hypothetical protein H665_p140	Ostreococcus tauri virus 1	YP_003212963	48.40%	1.81E-69	251(7-257)	TBP_TLF(cl08263,1.52e-11,88,146-233)	TATA-box binding protein-like（SSF55945，6.6E-13，139-234）
81	66,413	66,655	243	80	hypothetical protein	Ruminococcus sp. JC304	WP_019163451	36.10%	2.40E-05	86(148-233)		

ID	Position		Length		BlastP hit in GenBank nr and/or viruses data set						NCBI conserved domain (ID, E-value, alignment length in aa, alignment start - end position)	Interproscan matches (ID, E-value, alignment start - end position)
	start	end	nt	aa	ORF, protein encoded, or mass	Species	Accession	Aa identity	E Value	Alignment length in aa (Start-end position)		
82	66,731	67,381	651	216	kinase protein	Acanthocystis turfacea Chlorella virus Canal-1	AGE50200	35.70%	2.63E-22	165(1-165)	Tmk(COG0125,5.81e-15,179,2-180)	Deoxynucleoside kinase(PIRSF000705,1.6E-12,1-187)
					Chain A, Crystal Structure Of Drosophila Deoxyribonucleoside Kinase	Drosophila melanogaster	1J90_A	32.80%	3.50E-19	172(2-173)		P-loop containing nucleoside triphosphate hydrolases(SSF52540,5.4E-33,1-180)
83	67,461	67,661	201	66								
84	67,634	68,113	480	159	hypothetical protein OtV6_143	Ostreococcus tauri virus RT-2011	AFC35051	30.20%	5.42E-11	123(32-154)	RING(cl17238,1.32e-07,49,32-80)	zf-RING_2(PF13639,1.1E-11,32-77)
					PREDICTED: RING finger protein 141-like	Bombus terrestris	XP_003401476	35.50%	2.35E-09	62(19-80)		RING FINGER PROTEIN 38(PTHR14155:SF2,6.5E-9,32-99)
85	68,119	69,024	906	301	hypothetical protein BpV2_144c	Bathycoccus sp. RCC1105 virus BpV2	ADQ91311	38.70%	3.43E-49	250(47-296)		
					hypothetical protein VOLCADRAFT_119457	Volvox carteri f. nagariensis	XP_002956623	27.00%	1.37E-11	277(23-299)		
86	69,072	69,275	204	67	hypothetical protein MpV1_149	Micromonas sp. RCC1109 virus MpV1	YP_004062032	31.80%	1.71E-05	66(1-66)		signal-peptide(SignalP-NN(euk),-1.0,1-40)

ID	Position		Length		BlastP hit in GenBank nr and/or viruses data set						NCBI conserved domain (ID, E-value, alignment length in aa, alignment start - end position)	Interproscan matches (ID, E-value, alignment start - end position)
	start	end	nt	aa	ORF, protein encoded, or mass	Species	Accession	Aa identity	E Value	Alignment length in aa (Start-end position)		
												transmembrane_regions(tmhmm,-1.0,15-35;45-65)
87	69,321	69,614	294	97	hypothetical protein H665_p144	Ostreococcus tauri virus 1	YP_003212967	41.50%	5.33E-11	92(6-97)	Thioredoxin_like(cl00388,5.66e-11,57,38-96)	Thioredoxin-like（SSF52833，3.5E-11，26-96）
					glutaredoxin-like protein	Clostridium cellulovorans 743B	YP_003845639	38.10%	1.03E-05	56(39-94)		Glutaredoxin（PF00462，3.1E-7，39-72）
88	69,619	71,709	2091	696	hypothetical protein MpV1_151c	Micromonas sp. RCC1109 virus MpV1	YP_004062034	30.70%	5.51E-15	257(282-538)	PKc_like(cl09925,1.69e-07,187,194-380)	PROTEIN_KINASE_DOM （PS50011，10.464，188-506）
					hypothetical protein CAPTEDRAFT_105698	Capitella teleta	ELU04274	41.80%	6.56E-03	67(304-370)		
89	71,728	72,408	681	226	hypothetical protein MPXG_00075	Micromonas pusilla virus SP1	AET84873	31.50%	3.41E-24	210(1-210)		
90	72,491	73,573	1083	360	translation initiation factor IF-2	Mycobacterium intracellulare ATCC 13950	YP_005339067	48.90%	1.47E-02	41(85-125)		
91	73,597	73,887	291	96	hypothetical protein OlV1_127c	Ostreococcus lucimarinus virus OlV1	YP_004061760	46.40%	6.01E-09	56(4-59)		signal-peptide(SignalP-NN(euk),-1.0,1-16)
												transmembrane_regions(tmhmm,-1.0,42-62)

ID	Position		Length		BlastP hit in GenBank nr and/or viruses data set						NCBI conserved domain (ID, E-value, alignment length in aa, alignment start - end position)	Interproscan matches (ID, E-value, alignment start - end position)
	start	end	nt	aa	ORF, protein encoded, or mass	Species	Accession	Aa identity	E Value	Alignment length in aa (Start-end position)		
92	73,911	74,204	294	97	hypothetical protein MPVG_00082	Micromonas pusilla virus 12T	YP_007676150	42.60%	3.38E-05	54(7-60)		signal-peptide(SignalP-NN(euk),-1.0,1-24)
												transmembrane_regions(tmhmm,-1.0,5-23;42-60)
93	74,226	74,414	189	62								
94	74,411	75,361	951	316	hypothetical protein OtV6_156c	Ostreococcus tauri virus RT-2011	AFC35064	62.90%	9.82E-142	315(2-316)	RNRR2(cd01049,4.32e-115,275,12-286)	RIBONUCLEOSIDE-DIPHOSPHATE REDUCTASE SMALL CHAIN(PTHR23409,3.4E-184,2-316)
					PREDICTED: ribonucleoside-diphosphate reductase small chain-like	Glycine max	XP_003547645	58.30%	9.39E-137	315(2-316)		
95	75,454	75,873	420	139	hypothetical protein MPWG_00044	Micromonas pusilla virus PL1	AET43534	47.20%	7.76E-12	107(12-118)		
					hypothetical protein	Verrucomicrobia bacterium SCGC AAA164-N20	WP_020036363	46.00%	3.72E-11	112(12-123)		
96	75,888	76,601	714	237	hypothetical protein	Arthrobacter sp. 131MFCol6.1	WP_018773486	41.60%	1.83E-45	224(14-237)	Glyco_hydro_cc(pfam11790,1.88e-48,191,44-234)	Glyco_hydro_cc(PF11790,9.1E-51,13-234)
97	76,949	77,407	459	152								

ID	Position		Length		BlastP hit in GenBank nr and/or viruses data set						NCBI conserved domain (ID, E-value, alignment length in aa, alignment start - end position)	Interproscan matches (ID, E-value, alignment start - end position)
	start	end	nt	aa	ORF, protein encoded, or mass	Species	Accession	Aa identity	E Value	Alignment length in aa (Start-end position)		
98	77,396	77,956	561	186								signal-peptide(SignalP-NN(euk),-1.0,1-26)<br>transmembrane_regions(tmhmm,-1.0,9-29)
99	77,975	78,382	408	135								
100	78,412	78,816	405	134							SEC14(cl15787,4.48e-04,121,5-125)	CRAL_TRIO（PF00650,1.4E-4,45-133）
101	78,921	79,244	324	107	DSBA oxidoreductase	Burkholderia sp. H160	WP_008922466	32.50%	2.41E-02	83(7-89)		signal-peptide(SignalP-NN(euk),-1.0,1-23)<br>transmembrane_regions(tmhmm,-1.0,4-22;43-61)
102	79,237	79,434	198	65								PROKAR_LIPOPROTEIN(PS51257,5.0,1-17)<br>signal-peptide(SignalP-NN(euk),-1.0,1-20)<br>transmembrane_regions(tmhmm,-1.0,10-28;42-60)
103	80,055	80,219	165	54								

ID	Position		Length		BlastP hit in GenBank nr and/or viruses data set						NCBI conserved domain (ID, E-value, alignment length in aa, alignment start - end position)	Interproscan matches (ID, E-value, alignment start - end position)
	start	end	nt	aa	ORF, protein encoded, or mass	Species	Accession	Aa identity	E Value	Alignment length in aa (Start-end position)		
104	80,284	80,877	594	197	conserved unknown protein	Ectocarpus siliculosus	CBN79497	33.70%	4.94E-05	79(116-194)	zf-C3HC4_2(pfam13923,1.82e-06,41,146-186)	ZF_RING_2(PS50089,11.191,149-185)<br>zf-C3HC4_3(PF13920,2.0E-10,146-190)
105	81,061	81,423	363	120								
106	81,446	81,643	198	65								signal-peptide(SignalP-NN(euk),-1.0,1-25)<br>transmembrane_regions(tmhmm,-1.0,10-30;40-58)
107	81,648	81,974	327	108	S-adenosylmethionine decarboxylase	Symbiobacterium thermophilum IAM 14863	YP_074250	41.50%	3.16E-14	93(13-105)	AdoMet_dc super family(cl00687,1.25e-25,83,23-105)	AdoMet_dc(PF02675,3.4E-22,16-96)
108	81,984	82,403	420	139								
109	82,404	82,808	405	134								signal-peptide(SignalP-NN(euk),-1.0,1-38)<br>transmembrane_regions(tmhmm,-1.0,23-41)
110	82,948	84,351	1404	467	hypothetical protein OsV5_190f	Ostreococcus virus OsV5	YP_001648266	63.10%	0	447(21-467)	Capsid_NCLDV(pfam04451,1.63e-59,242,222-463)	Capsid_NCLDV（PF04451,1.6E-69,221-463)

ID	Position		Length		BlastP hit in GenBank nr and/or viruses data set						NCBI conserved domain (ID, E-value, alignment length in aa, alignment start - end position)	Interproscan matches (ID, E-value, alignment start - end position)
	start	end	nt	aa	ORF, protein encoded, or mass	Species	Accession	Aa identity	E Value	Alignment length in aa (Start-end position)		
												Group II dsDNA viruses VP(SSF49749,2.8E-55,45-241; 2.4E-45,242-467)
												African swine fever virus, p72, major capsid(2.70.9.20,1.2E-63,244-467)
111	84,382	84,657	276	91	hypothetical protein MPWG_00084	Micromonas pusilla virus PL1	AET43573	44.80%	1.01E-02	65(20-84)		
	84,686	86,623	1938	645	hypothetical protein MPVG_00067	Micromonas pusilla virus 12T	YP_007676135	50.60%	0	542(98-639)	COG3378(COG3378,1.64e-09,157,369-525)	SF3_HELICASE_1(PS51206,11.757,331-505)
112												P-loop containing nucleoside triphosphate hydrolases(SSF52540,3.9E-10,345-506)
113	86,623	88,833	2211	736								
	88,814	89,404	591	196	hypothetical protein OtV2_147	Ostreococcus tauri virus 2	YP_004063580	45.00%	1.72E-38	185(11-195)		mRNA_triPase(PF02940,1.2E-12,33-169)
114											CYTH-like_Pase(cl11964,8.97e-09,123,70-192)	CYTH-like phosphatases(SSF55154,2.8E-12,23-196)

ID	Position		Length		BlastP hit in GenBank nr and/or viruses data set						NCBI conserved domain (ID, E-value, alignment length in aa, alignment start - end position)	Interproscan matches (ID, E-value, alignment start - end position)
	start	end	nt	aa	ORF, protein encoded, or mass	Species	Accession	Aa identity	E Value	Alignment length in aa (Start-end position)		
115	89,202	90,218	1017	338	hypothetical protein OtV6_162c	Ostreococcus tauri virus RT-2011	AFC35070	38.00%	2.40E-54	261(75-335)	UCH(pfam00443,4.60e-24,244,81-324)	UCH_2_3(PS50235,17.907,81-338)
					ubiquitin carboxyl-terminal hydrolase 12	Arthroderma otae CBS 113480	XP_002846325	34.80%	3.88E-13	105(77-181)		Cysteine proteinases(SSF54001,2.2E-40,77-337)
116	90,232	90,750	519	172								
117	90,756	91,634	879	292	hypothetical protein MpV1_160	Micromonas sp. RCC1109 virus MpV1	YP_004062043	40.20%	4.88E-57	273(17-289)	Adenylation_DNA_ligase_like(cl12015,8.29e-47,187,15-201)	MRNA-CAPPING ENZYME(PTHR10367,3.7E-33,24-288)
118	91,702	92,337	636	211	hypothetical protein MpV1_159	Micromonas sp. RCC1109 virus MpV1	YP_004062042	35.80%	2.83E-37	210(1-210)		
119	92,365	93,120	756	251	hypothetical protein MpV1_158	Micromonas sp. RCC1109 virus MpV1	YP_004062041	64.70%	5.78E-118	238(6-243)		Nucleotide-diphospho-sugar transferases（SSF53448，2.3E-10，1-233）
120	93,148	94,125	978	325	hypothetical protein OtV6_158	Ostreococcus tauri virus RT-2011	AFC35066	39.80%	8.08E-68	319(7-325)	SUA7(COG1405,5.82e-24,278,32-309)	TRANSCRIPTION INITIATION FACTOR IIB-RELATED（PTHR11618，2.9E-33，8-316）
					transcription initiation factor IIB	Archaeoglobus fulgidus DSM 4304	NP_070128	28.20%	1.20E-21	290(24-313)		
121	94,189	94,479	291	96								transmembrane_regions(tmhmm,-1.0,25-

ID	Position		Length		BlastP hit in GenBank nr and/or viruses data set						NCBI conserved domain (ID, E-value, alignment length in aa, alignment start - end position)	Interproscan matches (ID, E-value, alignment start - end position)
	start	end	nt	aa	ORF, protein encoded, or mass	Species	Accession	Aa identity	E Value	Alignment length in aa (Start-end position)		
												43)
122	94,466	95,587	1122	373	hypothetical protein MPWG_00064	Micromonas pusilla virus PL1	AET43554	31.80%	8.38E-40	332(39-370)	RFC1(pfam08519,1.68e-04,128,243-370)	DNA polymerase III clamp loader subunits, C-terminal domain(SSF48019,7.0E-6,223-314)
					hypothetical protein VICG_01339	Vittaforma corneae ATCC 50505	ELA41591	23.60%	3.67E-03	223(50-272)		signal-peptide(SignalP-NN(euk),-1.0,1-20)
123	95,672	95,977	306	101	hypothetical protein PBCVMA1D_454L	Paramecium bursaria Chlorella virus MA-1D	AGE54931	55.70%	2.78E-25	92(3-94)		
124	96,308	96,907	600	199								
125	96,931	97,335	405	134	putative phage N-6-adenine-methyltransferase	Clostridium botulinum	WP_003385021	45.00%	8.94E-25	129(5-133)	Dam(cl05442,7.87e-26,128,6-133)	Dam(PF05869,9.4E-27,6-133)
126	97,387	97,641	255	84							Peptidase_M6(cl11525,6.60e-03,29,32-60)	transmembrane_regions(tmhmm,-1.0,19-39;54-76)
127	97,686	98,816	1131	376	ornithine decarboxylase 1	Chlamydomonas reinhardtii	XP_001697502	44.80%	1.36E-99	362(7-368)	LysA(COG0019,2.57e-73,340,20-359)	ARGININE/DIAMINOPIMELATE/ORNITHINE DECARBOXYLASE(PTHR11482,6.3E-119,18-359)
128	99,003	99,584	582	193	hypothetical protein MpV1_184c	Micromonas sp. RCC1109 virus MpV1	YP_004062067	43.30%	4.32E-43	178(11-188)	Clp_protease_like(cl15763,2.99e-27,131,62-192)	ClpP/crotonase(SSF52096,1.1E-33,3-193)

ID	Position		Length		BlastP hit in GenBank nr and/or viruses data set						NCBI conserved domain (ID, E-value, alignment length in aa, alignment start - end position)	Interproscan matches (ID, E-value, alignment start - end position)
	start	end	nt	aa	ORF, protein encoded, or mass	Species	Accession	Aa identity	E Value	Alignment length in aa (Start-end position)		
129	99,663	100,571	909	302	ATP-dependent Clp protease, proteolytic subunit	Phenylobacterium zucineum HLK1	YP_002130657	35.30%	2.28E-17	134(60-193)		
					hypothetical protein FR483_N500R	Paramecium bursaria Chlorella virus FR483	YP_001426132	34.40%	8.15E-41	238(65-302)		
					hypothetical protein SDRG_07749	Saprolegnia diclina VS20	EQC34951	31.80%	4.16E-37	291(11-301)		
130	100,596	100,823	228	75								signal-peptide(SignalP-NN(euk),-1.0,1-21)
131	100,843	101,472	630	209	hypothetical protein MpV1_231c	Micromonas sp. RCC1109 virus MpV1	YP_004062114	53.40%	1.63E-71	202(4-205)	PhoH(cl17668,4.63e-86,202,4-205)	PhoH(PF02562,3.5E-70,3-205)
					phosphate starvation protein PhoH	Verrucomicrobia bacterium SCGC AAA164-N20	WP_020036220	48.80%	3.80E-65	202(4-205)		P-loop containing nucleoside triphosphate hydrolases(SSF52540,2.5E-30,2-185)
132	101,526	101,900	375	124							tolB(PRK04922,1.54e-03,56,44-99)	
133	101,908	103,233	1326	441	uncharacterized protein BN772_00442	Bacteroides sp. CAG:754	CDA86821	27.30%	1.12E-06	148(39-186)	HNHc(cl00083,2.45e-05,54,378-431)	HNH(PF01844,4.4E-7,387-432)
134	103,754	104,101	348	115								transmembrane_regions(tmhmm,-1.0,33-53)
135	104,020	104,409	390	129	PREDICTED: SCF ubiquitin ligase complex protein SKP1b-like	Glycine max	XP_003545933	34.50%	1.08E-09	102(26-127)	SKP1(COG5201,1.29e-07,107,21-127)	SKP1(PTHR11165,2.6E-6,26-127)
					SKP1-like protein	Paramecium bursaria Chlorella virus AP110A	AGE48994	30.80%	4.58E-09	104(24-127)		

ID	Position		Length		BlastP hit in GenBank nr and/or viruses data set						NCBI conserved domain (ID, E-value, alignment length in aa, alignment start - end position)	Interproscan matches (ID, E-value, alignment start - end position)
	start	end	nt	aa	ORF, protein encoded, or mass	Species	Accession	Aa identity	E Value	Alignment length in aa (Start-end position)		
136	104,535	104,768	234	77								
137	104,792	105,454	663	220	pyrimidine 5-nucleotidase	Dacryopinax sp. DJM-731 SS1	EJU06105	30.60%	9.58E-03	155(41-195)	HAD_2(pfam13419,3.92e-04,6-186)	HAD-like(SSF56784,1.6E-6,1-206)
138	105,484	105,723	240	79								signal-peptide(SignalP-NN(euk),-1.0,1-39) transmembrane_regions(tmhmm,-1.0,19-37;52-72)
139	105,763	106,197	435	144	hypothetical protein H665_p199	Ostreococcus tauri virus 1	YP_003213022	57.30%	5.98E-40	143(1-143)	trimeric_dUTPase(cl00493,5.62e-28,90,23-112)	DEOXYURIDINE 5'-TRIPHOSPHATE NUCLEOTIDOHYDROLASE(PTHR11241,7.9E-56,1-144)
140	106,235	106,627	393	130	DUTP diphosphatase	Oxytricha trifallax	EJY68335	47.90%	1.42E-36	142(3-144)		dUTPase(PF00692,6.4E-35,12-143) signal-peptide(SignalP-NN(euk),-1.0,1-33)
141	106,665	109,589	2925	974	hypothetical protein SXBG_00214	Synechococcus phage S-CAM1	YP_007673127	41.20%	7.25E-83	446(513-958)		
					hypothetical protein	alpha proteobacterium SCGC AAA076-E06	WP_016579595	35.00%	7.64E-07	100(583-682)		
142	109,609	110,340	732	243								
143	110,382	112,988	2607	868	hypothetical protein MpV1_220	Micromonas sp. RCC1109 virus MpV1	YP_004062103	54.60%	0	842(4-845)	PolB(COG0417,3.37e-129,849,17-865)	pol2: DNA polymerase (pol2)(TIGR00592,1.7E-76,457-842)
					PREDICTED: DNA polymerase delta catalytic subunit	Vitis vinifera	XP_002264385	37.90%	3.30E-167	845(18-862)	DnaQ_like_exo(cl10012,2.40e-59,225,182-406)	DNA_pol_B_exo1(PF03104,3.6E-48,26-349)

ID	Position		Length		BlastP hit in GenBank nr and/or viruses data set						NCBI conserved domain (ID, E-value, alignment length in aa, alignment start - end position)	Interproscan matches (ID, E-value, alignment start - end position)
	start	end	nt	aa	ORF, protein encoded, or mass	Species	Accession	Aa identity	E Value	Alignment length in aa (Start-end position)		
											POLBc(cl10023,3.19e-136,390,456-845)	DNA_pol_B(PF00136,9.5E-116,423-849)
144	112,975	113,265	291	96	unknown	Zea mays	ACR34968	41.30%	1.09E-07	80(6-85)	Ion_trans_2(pfam07885,1.76e-05,41,43-83)	
145	113,326	113,646	321	106	hypothetical protein MPXG_00150	Micromonas pusilla virus SP1	AET84948	40.00%	2.65E-08	74(4-77)		
146	113,662	114,129	468	155	hypothetical protein BpV2_191	Bathycoccus sp. RCC1105 virus BpV2	ADQ91358	55.80%	2.49E-41	123(1-123)		
147	114,058	116,358	2301	766	hypothetical protein MPWG_00028	Micromonas pusilla virus PL1	AET43518	28.30%	5.53E-49	425(336-760)	Capsid_NCLDV(pfam04451,5.73e-20,178,586-763)	Capsid_NCLDV(PF04451,8.3E-27 ,554-762)
												Group II dsDNA viruses VP(SSF49749,8.4E-29,353-571;1.2E-22,572-766)
												African swine fever virus(2.70.9.20,2.0E-27,575-765)
148	116,359	119,514	3156	1051	hypothetical protein OtV6_232c	Ostreococcus tauri virus RT-2011	AFC35140.1	52.00%	0	1048(4-1051)	HATPase_c(cl00075,2.21e-03,111,45-155)	Ribosomal protein S5 domain 2-like(SSF54211,1.3E-23,225-370)
					hypothetical protein CHLNCDRAFT_137783	Chlorella variabilis	EFN59062.1	44.00%	0	1046(3-1048)	PLN03128(PLN03128,0,1050,2-1051)	DNA TOPOISOMERASE 2(PTHR10169:SF19,0,2-1051)

ID	Position		Length		BlastP hit in GenBank nr and/or viruses data set						NCBI conserved domain (ID, E-value, alignment length in aa, alignment start - end position)	Interproscan matches (ID, E-value, alignment start - end position)
	start	end	nt	aa	ORF, protein encoded, or mass	Species	Accession	Aa identity	E Value	Alignment length in aa (Start-end position)		
											TopoII_MutL_Trans(cl02783,5.17e-27,130,236-365)<br>TOPRIM_TopoIIA(cd03365,2.18e-62,118,407-524)<br>TOP4c Superfamily(cl00148,3.04e-97,410,642-1051)	
149	119,538	120,194	657	218								signal-peptide(SignalP-NN(euk),-1.0,1-35)<br>transmembrane_regions(tmhmm,-1.0,9-31;45-65;74-92;120-138;153-175;189-209)
150	120,195	120,659	465	154	thiol oxidoreductase	Turbot reddish body iridovirus	ADE34387	34.70%	7.26E-14	98(24-121)	Evr1_Alr(cl02107,1.25e-16,89,26-114)	FAD-dependent thiol oxidase(SSF69000,1.2E-21,15-128)
					FAD dependent sulfhydryl oxidase Erv2	Pyrenophora tritici-repentis Pt-1C-BFP	XP_001933180	38.60%	1.60E-10	83(29-111)		ALR/ERV(PTHR12645,6.1E-10,29-111)
151	120,641	121,984	1344	447								
152	121,934	122,593	660	219								
153	122,586	122,861	276	91	hypothetical protein OtV2_215	Ostreococcus tauri virus 2	YP_004063648	39.70%	1.60E-04	58(11-68)		signal-peptide(SignalP-NN(euk),-1.0,1-27)

ID	Position		Length		BlastP hit in GenBank nr and/or viruses data set						NCBI conserved domain (ID, E-value, alignment length in aa, alignment start - end position)	Interproscan matches (ID, E-value, alignment start - end position)
	start	end	nt	aa	ORF, protein encoded, or mass	Species	Accession	Aa identity	E Value	Alignment length in aa (Start-end position)		
154	122,915	123,343	429	142								transmembrane_regions(tmhmm,-1.0,5-23;47-67)
155	123,321	124,262	942	313	hypothetical protein MPXG_00160	Micromonas pusilla virus SP1	AET84958	44.20%	3.31E-80	296(11-306)	RfaG(COG0438,1.30e-11,161,89-249)	Glycos_transf_1(PF00534,1.4E-10,137-249)
					glycosyl transferase family 1	Aquimarina agarilytica	WP_010182818	24.20%	1.68E-11	218(76-293)		UDP-Glycosyltransferase/glycogen phosphorylase(SSF53756,6.6E-15,89-249)
156	124,259	125,269	1011	336	GDP-mannose 4,6-dehydratase	Methanococcus maripaludis C7	YP_001329560	58.90%	3.52E-132	334(1-334)	Gmd(COG1089,2.60e-174,334,1-334)	NAD DEPENDENT EPIMERASE/DEHYDRATASE(PTHR10366,2.2E-161,1-334)
												gmd: GDP-mannose 4,6-dehydratase(TIGR01472,4.4E-139,2-335)
												NAD(P)-binding Rossmann-fold domains(SSF51735,1.2E-67,2-336)
157	125,276	125,446	171	56								

ID	Position		Length		BlastP hit in GenBank nr and/or viruses data set						NCBI conserved domain (ID, E-value, alignment length in aa, alignment start - end position)	Interproscan matches (ID, E-value, alignment start - end position)
	start	end	nt	aa	ORF, protein encoded, or mass	Species	Accession	Aa identity	E Value	Alignment length in aa (Start-end position)		
158	125,485	125,817	333	110							Multi_Drug_Res(cl00910,5.71e-06,105,1-105)	
159	125,838	126,122	285	94	hypothetical protein MpV1_191c	Micromonas sp. RCC1109 virus MpV1	YP_004062074	33.30%	6.97E-04	72(1-72)		
160	126,146	126,478	333	110								signal-peptide(SignalP-NN(euk),-1.0,1-24)
161	126,479	127,240	762	253	putative N-acetylglucosaminyltransferase	Flavobacteria bacterium BAL38	WP_008254071	33.00%	2.70E-29	228(1-228)	Glyco_transf_17(pfam04724,3.28e-30,243,3-245)	Glyco_transf_17(PF04724,3.7E-35,3-230)
162	127,247	127,912	666	221	hypothetical protein MpV1_031c	Micromonas sp. RCC1109 virus MpV1	YP_004061914	42.00%	9.91E-59	217(3-219)	Glyco_transf_34 Superfamily(cl05288,2.69e-19,178,23-200)	Glyco_transf_34(PF05637,9.0E-16,21-89;2.5E-5,94-201)
					hypothetical protein COCSUDRAFT_54970	Coccomyxa subellipsoidea C-169	EIE18426	28.10%	8.09E-16	196(24-219)		
163	127,919	128,392	474	157	hypothetical protein MPXG_00213	Micromonas pusilla virus SP1	AET85011	65.80%	1.84E-74	156(1-156)	TPP_PYR_DXS_TK_like(cd07033, 5.16e-34,152,5-156)	Transket_pyr(PF02779,5.4E-13,1-133)
					hypothetical protein ACD_12C00312G0008	uncultured bacterium	EKE14751	52.50%	1.28E-52	156(1-156)		Thiamin diphosphate-binding fold (THDP-binding)(SSF52518,1.6E-25,1-157)

ID	Position		Length		BlastP hit in GenBank nr and/or viruses data set						NCBI conserved domain (ID, E-value, alignment length in aa, alignment start - end position)	Interproscan matches (ID, E-value, alignment start - end position)
	start	end	nt	aa	ORF, protein encoded, or mass	Species	Accession	Aa identity	E Value	Alignment length in aa (Start-end position)		
164	128,298	129,002	705	234	transketolase domain-containing protein	Micromonas pusilla virus SP1	AET85010	68.10%	3.52E-99	209(1-209)	TPP_enzymes super family(cl01629,3.36e-31,150,3-152)	Transketolase_N(PF00456,5.6E-12,7-155)
					hypothetical protein ACD_12C00312G0009	uncultured bacterium	EKE14752	40.20%	4.87E-45	195(1-195)		Thiamin diphosphate-binding fold (THDP-binding)(SSF52518,1.5E-27,5-165)
165	128,983	129,828	846	281	UDP-glucose 4-epimerase	Cystobacter fuscus	WP_020918602	30.20%	2.43E-25	277(1-277)	SDR_e(cd08946,2.01e-34,203,3-205)	NAD DEPENDENT EPIMERASE/DEHYDRATASE(PTHR10366,2.0E-38,1-274)
											WcaG(COG0451,3.76e-39,275,1-275)	NAD(P)-binding Rossmann-fold domains(SSF51735,2.7E-51,2-273)
166	129,911	130,612	702	233	hypothetical protein MPXG_00211	Micromonas pusilla virus SP1	AET85009	48.60%	6.57E-64	230(3-232)	Methyltransf_21 super family(cl17387,1.09e-18,136,56-191)	fkbM_fam: methyltransferase, FkbM family(TIGR01444,1.6E-21,56-191)

ID	Position		Length		BlastP hit in GenBank nr and/or viruses data set						NCBI conserved domain (ID, E-value, alignment length in aa, alignment start - end position)	Interproscan matches (ID, E-value, alignment start - end position)
	start	end	nt	aa	ORF, protein encoded, or mass	Species	Accession	Aa identity	E Value	Alignment length in aa (Start-end position)		
					putative methyltransferase	Cyclobacteriaceae bacterium AK24	WP_010853020	28.10%	9.33E-12	159(33-191)		S-adenosyl-L-methionine-dependent methyltransferases(SSF53335,3.2E-6,33-103)
167	130,646	131,593	948	315	NAD dependent epimerase/dehydratase family protein	Trichomonas vaginalis G3	XP_001297395	48.10%	3.63E-96	306(3-308)	dTDP_GD_SDR_e(cd05246,1.17e-139,313,1-313)	DTDP-GLUCOSE 4,6-DEHYDRATASE(PTHR10366:SF41,1.3E-132,1-314)
												NAD DEPENDENT EPIMERASE/DEHYDRATASE(PTHR10366,1.3E-132,1-314)
168	131,580	131,936	357	118	hypothetical protein BpV2_001c	Bathycoccus sp. RCC1105 virus BpV2	ADQ91168	51.30%	1.97E-27	113(1-113)	rI.-1(PHA02604,3.53e-18,111,7-117)	
					hypothetical protein	Salmonella phage PhiSH19	YP_007008052	33.90%	2.05E-10	115(1-115)		
169	131,959	132,399	441	146								
170	132,396	132,776	381	126								
171	133,045	133,602	558	185								
172	133,610	133,810	201	66								
173	133,861	134,031	171	56	predicted protein	Micromonas sp. RCC299	XP_002503824	72.90%	7.71E-03	48(9-56)		

ID	Position		Length		BlastP hit in GenBank nr and/or viruses data set						NCBI conserved domain (ID, E-value, alignment length in aa, alignment start - end position)	Interproscan matches (ID, E-value, alignment start - end position)
	start	end	nt	aa	ORF, protein encoded, or mass	Species	Accession	Aa identity	E Value	Alignment length in aa (Start-end position)		
174	134,012	134,260	249	82								
175	134,310	134,543	234	77								
176	134,586	134,774	189	62								
177	134,773	135,228	456	151								
178	135,253	135,489	237	78								
179	135,567	136,472	906	301	vanillate demethylase oxygenase subunit	Streptosporangium roseum DSM 43021	YP_003342065	32.10%	1.39E-21	156(4-159)	Rieske super family(cl00938,6.11e-18,112,4-115)	ISP domain(SSF50022,2.6E-18,3-107)
											HcaE(COG4638,5.37e-27,293,3-295)	Bet v1-like(SSF55961,1.1E-18,98-301)
180	136,666	137,073	408	135	putative phage N-6-adenine-methyltransferase	Clostridium botulinum	WP_003385021	47.50%	9.37E-28	130(5-134)	Dam(cl05442,1.43e-29,129,6-134)	Dam(PF05869,1.6E-28,6-134)
181	137,098	137,322	225	74								
182	137,319	138,032	714	237								
183	138,264	138,791	528	175								
184	139,099	139,596	498	165	GNAT family acetyltransferase	Streptococcus henryi	WP_018164889	32.50%	5.93E-09	82(44-125)	NAT_SF(cd04301,1.92e-07,57,51-107)	Acyl-CoA N-acyltransferases (Nat)(SSF55729,7.3E-17,24-132)
185	139,614	140,180	567	188	hypothetical protein	Lachnospiraceae bacterium 3-2	WP_016222948	29.60%	3.35E-02	80(108-187)		
186	140,211	140,714	504	167								
187	140,739	141,179	441	146	hypothetical protein BpV1_044c	Bathycoccus sp. RCC1105 virus BpV1	YP_004061474	40.90%	4.22E-10	114(13-126)		
					hypothetical protein	Verrucomicrobia bacterium SCGC AAA164-N20	WP_020036363	40.00%	1.40E-09	114(13-126)		

ID	Position		Length		BlastP hit in GenBank nr and/or viruses data set						NCBI conserved domain (ID, E-value, alignment length in aa, alignment start - end position)	Interproscan matches (ID, E-value, alignment start - end position)
	start	end	nt	aa	ORF, protein encoded, or mass	Species	Accession	Aa identity	E Value	Alignment length in aa (Start-end position)		
188	141,176	141,469	294	97								
189	141,475	142,383	909	302	hypothetical protein SDRG_07749	Saprolegnia diclina VS20	EQC34951	32.10%	5.09E-38	291(11-301)		
					hypothetical protein ATCVBr0604L_169R	Acanthocystis turfacea Chlorella virus Br0604L	AGE49070	33.50%	1.23E-37	238(65-302)		
190	142,401	143,075	675	224	hypothetical protein MYCTH_2050929	Myceliophthora thermophila ATCC 42464	XP_003660640	31.00%	2.13E-05	175(5-179)	Pyr-5-nucltdase(TIGR01993,9.41e-09,183,5-187)	HAD_2(PF13419,3.3E-7,8-179)
191	143,079	143,858	780	259								
192	144,078	144,242	165	54								transmembrane_regions(tmhmm,-1.0,21-41)
193	144,414	144,887	474	157	hypothetical protein OlV1_207	Ostreococcus lucimarinus virus OlV1	YP_004061839	56.60%	2.03E-41	143(14-156)	trimeric_dUTPase(cl00493,1.40e-26,89,37-125)	
					dUTPase	Pfiesteria piscicida	ACU44961	52.80%	2.85E-36	142(15-156)		
194	144,904	145,380	477	158								transmembrane_regions(tmhmm,-1.0,19-41;56-76;82-100)
195	145,410	148,031	2622	873	hypothetical protein BpV2_189	Bathycoccus sp. RCC1105 virus BpV2	ADQ91356	51.30%	0	837(1-837)	PolB(COG0417,3.61e-137,817,21-837)	pol2: DNA polymerase (pol2)(TIGR00592,3.0E-75,445-835)
											DnaQ_like_exo(cl10012,2.62e-69,223,173-395)	DNA_pol_B_exo1(PF03104,6.7E-46,37-340)

ID	Position		Length		BlastP hit in GenBank nr and/or viruses data set						NCBI conserved domain (ID, E-value, alignment length in aa, alignment start - end position)	Interproscan matches (ID, E-value, alignment start - end position)
	start	end	nt	aa	ORF, protein encoded, or mass	Species	Accession	Aa identity	E Value	Alignment length in aa (Start-end position)		
											POLBc(cl10023,4.03e-134,390,447-836)	DNA_pol_B(PF00136,2.9E-114,414-850)
196	148,062	148,349	288	95	hypothetical protein MPWG_00030	Micromonas pusilla virus PL1	AET43520	34.60%	2.54E-10	77(1-77)		
197	148,377	148,850	474	157	hypothetical protein BpV1_186	Bathycoccus sp. RCC1105 virus BpV1	YP_004061616	57.40%	5.60E-43	116(1-116)		
198	148,738	151,074	2337	778	hypothetical protein BpV1_187c	Bathycoccus sp. RCC1105 virus BpV1	YP_004061617	30.90%	4.03E-52	428(348-775)	Capsid_NCLDV(pfam04451,5.73e-20,178,586-763)	Capsid_NCLDV(PF04451,5.9E-23 ,565-774)
												Group II dsDNA viruses VP(SSF49749,3.4E-25,375-583;2.3E-21,584-778)
												African swine fever virus(2.70.9.20,3.0E-25,587-777)
199	151,077	154,280	3204	1067	DNA topoisomerase II	Ostreococcus tauri virus 2	YP_004063644	50.70%	0	1066(2-1067)	PLN03128(PLN03128,0,1065,3-1067)	DNA TOPOISOMERASE/GYRASE(PTHR10169,0.0,3-1064)
					DNA topoisomerase II	Enterocytozoon bieneusi H348	XP_002649941	42.80%	0	1064(3-1066)	TopoII_MutL_Trans(cl02783,1.51e-18,132,235-366)	
											TOPRIM_TopoIIA(cd03365,2.78e-61,118,407-524)	

ID	Position		Length		BlastP hit in GenBank nr and/or viruses data set						NCBI conserved domain (ID, E-value, alignment length in aa, alignment start - end position)	Interproscan matches (ID, E-value, alignment start - end position)
	start	end	nt	aa	ORF, protein encoded, or mass	Species	Accession	Aa identity	E Value	Alignment length in aa (Start-end position)		
											TOP4c Superfamily(cl00148,4.03e-103,412,656-1067)	
200	154,330	154,704	375	124	hypothetical protein EhV003	Emiliania huxleyi virus 86	YP_293757	51.90%	2.19E-10	53(14-66)		signal-peptide(SignalP-NN(euk),-1.0,1-51)
					hypothetical protein	Archaea	WP_018035822	48.40%	7.28E-09	59(1-59)		transmembrane_regions(tmhmm,-1.0,10-30;36-56;103-121)
201	154,714	155,088	375	124	erv-family thiol oxidoreductase	Pandoravirus dulcis	YP_008319152	43.50%	2.98E-15	81(9-89)	Evr1_Alr(cl02107,1.04e-16,81,10-90)	Evr1_Alr(PF04777,2.5E-16,12-90)
					predicted protein, partial	Physcomitrella patens subsp. patens	XP_001774132	43.50%	1.25E-08	66(24-89)		FAD-dependent thiol oxidase(SSF69000,5.8E-16,1-91)
202	155,098	155,763	666	221	hypothetical protein ATCVCanal1_254R	Acanthocystis turfacea Chlorella virus Canal-1	AGE50121	38.80%	4.58E-43	214(5-218)	AdoMet_MTases(cl17173,9.31e-06,81,57-137)	Methyltransf_24(PF13578,1.4E-9,57-163)
					hypothetical protein	Paenibacillus elgii	WP_010502997	29.90%	2.19E-15	164(51-214)		
203	155,787	156,278	492	163	peptidoglycan-binding protein LysM	Enterococcus mundtii	WP_010735712	38.70%	4.96E-02	62(101-162)		
204	156,303	156,683	381	126								
205	156,702	157,163	462	153	hypothetical protein MPVG_00242	Micromonas pusilla virus 12T	YP_007676306	53.30%	5.33E-04	30(124-153)		
206	157,178	157,432	255	84	hypothetical protein OLOG_00122	Ostreococcus lucimarinus virus OlV4	AET84583	41.40%	8.11E-07	58(9-66)		signal-peptide(SignalP-NN(euk),-1.0,1-24)

ID	Position		Length		BlastP hit in GenBank nr and/or viruses data set						NCBI conserved domain (ID, E-value, alignment length in aa, alignment start - end position)	Interproscan matches (ID, E-value, alignment start - end position)
	start	end	nt	aa	ORF, protein encoded, or mass	Species	Accession	Aa identity	E Value	Alignment length in aa (Start-end position)		
												transmembrane_regions(tmhmm,-1.0,4-24;49-69)
207	157,449	157,799	351	116								
208	157,759	158,703	945	314	hypothetical protein OtV6_236c	Ostreococcus tauri virus RT-2011	AFC35144	43.20%	1.02E-78	309(4-312)	RfaG(COG0438,8.97e-11,248,99-246)	GLYCOSYLTRANSFERASE(PTHR12526,5.2E-9,138-245)
					hypothetical protein	Sciscionella marina	WP_020499425	29.70%	8.55E-11	178(97-274)		Glycos_transf_1(PF00534,1.4E-8,193-246)<br>signal-peptide(SignalP-NN(euk),-1.0,1-22)
209	158,700	159,719	1020	339	GDP-mannose 4,6-dehydratase	Methanococcus maripaludis C7	YP_001329560	60.30%	3.04E-140	337(1-337)	Gmd(COG1089,0,337,1-337)	gmd: GDP-mannose 4,6-dehydratase(TIGR01472,3.5E-141,2-338)<br>NAD DEPENDENT EPIMERASE/DEHYDRATASE(PTHR10366,3.1E-171,1-337)
210	159,926	160,252	327	108	hypothetical protein	Acinetobacter bouvetii	WP_005009806.1	27.00%	0.82	102(1-102)	Multi_Drug_Res(cl00910,1.80e-04,102,1-102)	Multi_Drug_Res(F00893,8.1E-7,1-92)
211	160,276	160,569	294	97	hypothetical protein OLOG_00294	Ostreococcus lucimarinus virus OlV4	AET84745	40.30%	8.18E-03	62(14-75)		
212	160,490	161,287	798	265	N-acetylglucosaminyltransferase	Bdellovibrio bacteriovorus HD100	NP_968551	33.80%	1.01E-30	234(1-234)	Glyco_transf_17(pfam04724,1.37e-23,208,4-211)	Glyco_transf_17(PF04724,1.8E-26,4-238)

ID	Position		Length		BlastP hit in GenBank nr and/or viruses data set						NCBI conserved domain (ID, E-value, alignment length in aa, alignment start - end position)	Interproscan matches (ID, E-value, alignment start - end position)
	start	end	nt	aa	ORF, protein encoded, or mass	Species	Accession	Aa identity	E Value	Alignment length in aa (Start-end position)		
					glycosyltransferase	Paramecium bursaria Chlorella virus IL-5-2s1	AGE54320	29.40%	2.08E-20	228(2-229)		BETA-1,4-MANNOSYL-GLYCOPROTEIN BETA-1,4-N-ACETYLGLUCOSAMINYL-TRANSFERASE(PTHR12224,5.7E-15,4-121)
213	161,277	162,017	741	246	hypothetical protein BpV1_025	Bathycoccus sp. RCC1105 virus BpV1	YP_004061455	52.00%	1.96E-81	244(1-244)		
					conserved hypothetical protein, partial	Brucella suis	WP_004690517	45.50%	7.36E-15	96(4-99)		
214	162,022	162,999	978	325	hypothetical protein OlV1_020c	Ostreococcus lucimarinus virus OlV1	YP_004061653	36.60%	2.11E-29	208(16-223)	Methyltransf_21(cl17387,5.07e-05,49,91-139)	Methyltransf_21(PF05050,4.8E-6,91-152)
					hypothetical protein	Nitrospina sp. SCGC AAA288-L16	WP_017952561	33.60%	1.04E-27	222(2-223)		
215	163,019	163,372	354	117	hypothetical protein BpV2_001c	Bathycoccus sp. RCC1105 virus BpV2	ADQ91168	46.80%	4.94E-23	109(4-112)	rI.-1(PHA02604,5.50e-19,115,2-116)	
216	163,413	164,819	1407	468	hypothetical protein CC1G_07068	Coprinopsis cinerea okayama7#130	XP_001836421	22.90%	1.08E-02	160(154-313)		

ID	Position		Length		BlastP hit in GenBank nr and/or viruses data set						NCBI conserved domain (ID, E-value, alignment length in aa, alignment start - end position)	Interproscan matches (ID, E-value, alignment start - end position)
	start	end	nt	aa	ORF, protein encoded, or mass	Species	Accession	Aa identity	E Value	Alignment length in aa (Start-end position)		
217	164,829	165,494	666	221	putative prolyl 4-hydroxylase	Acanthamoeba polyphaga mimivirus	YP_003987108	44.20%	1.36E-53	189(33-221)	2OG-FeII_Oxy_3(cl17304,2.66e-26,170,48-217)	FE2OG_OXY(PS51471,10.012,107-218)
					oxidoreductase, 2OG-Fe(II) oxygenase family protein	Acidithiobacillus sp. GGI-221	WP_009569450	37.20%	9.34E-31	178(44-221)		PROLYL 4-HYDROXYLASE ALPHA SUBUNIT(PTHR10869,4.3E-29,35-221)
218	165,623	166,297	675	224	hypothetical protein OtV2_235	Ostreococcus tauri virus 2	YP_004063668	42.60%	8.59E-11	101(70-170)		
219	166,294	166,677	384	127	hypothetical protein BpV2_179	Bathycoccus sp. RCC1105 virus BpV2	ADQ91346	41.70%	1.91E-15	108(7-114)		signal-peptide(SignalP-NN(euk),-1.0,1-26)
												transmembrane_regions(tmhmm,-1.0,5-25;89-107)
220	166,757	167,395	639	212								
221	167,388	167,570	183	60								
222	167,567	168,949	1383	460	hypothetical protein OLNG_00139	Ostreococcus lucimarinus virus OlV5	YP_007674781	34.90%	4.66E-85	456(2-457)		ARIADNE RING ZINC FINGER(PTHR11685,4.8E-8,238-295)
					hypothetical protein	Trichomonas vaginalis G3	XP_001301335	44.00%	5.22E-16	90(209-298)		RING/U-box(SSF57850,5.4E-9,245-302)
223	168,955	169,821	867	288	mannosyltransferase	Acidovorax sp. MR-S7	WP_020226670	32.60%	3.95E-36	256(21-276)	Gly_transf_sug(pfam04488,1.73e-05,81,44-124)	Caps_synth(PF05704,2.3E-21,29-171)

ID	Position		Length		BlastP hit in GenBank nr and/or viruses data set						NCBI conserved domain (ID, E-value, alignment length in aa, alignment start - end position)	Interproscan matches (ID, E-value, alignment start - end position)
	start	end	nt	aa	ORF, protein encoded, or mass	Species	Accession	Aa identity	E Value	Alignment length in aa (Start-end position)		
224	169,845	170,021	177	58								signal-peptide(SignalP-NN(euk),-1.0,1-23)
225	170,428	170,799	372	123	hypothetical protein MAR_ORF180	Marseillevirus	YP_003406923	36.80%	4.11E-04	86(35-120)	T5orf172(cl17462,2.90e-03,88,35-122)	
YSLPV3												
1	344	1501	1158	385	hypothetical protein OtV6_076	Ostreococcus tauri virus RT-2011	AFC34984	45.80%	1.46E-94	385(1-385)	Capsid_NCLDV(pfam04451,1.68e-25,182,200-381)	Capsid_NCLDV(PF04451, 1.7E-21,200-381)
					hypothetical protein	Verrucomicrobia bacterium SCGC AAA164-N20	WP_020036256	41.30%	3.26E-85	385(1-385)		
2	1,524	2,621	1098	365	hypothetical protein MpV1_098c	Micromonas sp. RCC1109 virus MpV1	YP_004061981	30.90%	1.65E-28	357(2-358)		signal-peptide(SignalP-NN(euk),-1.0,1-15)
					hypothetical protein	Verrucomicrobia bacterium SCGC AAA164-N20	WP_020036348	28.40%	2.32E-18	350(8-357)		
3	2,612	2,929	318	105								
4	2,950	3,588	639	212	hypothetical protein OtV6_102c	Ostreococcus tauri virus RT-2011	AFC35010	42.10%	1.91E-43	186(2-187)		transmembrane_regions(tmhmm,-1.0,48-66)
					hypothetical protein	Verrucomicrobia bacterium SCGC AAA164-N20	WP_020036350	37.60%	5.23E-09	93(64-156)		
5	3,597	4,145	549	182	hypothetical protein	Bathycoccus sp.	YP_004061519	46.60%	6.94E-30	148(1-148)		

ID	Position		Length		BlastP hit in GenBank nr and/or viruses data set						NCBI conserved domain (ID, E-value, alignment length in aa, alignment start - end position)	Interproscan matches (ID, E-value, alignment start - end position)
	start	end	nt	aa	ORF, protein encoded, or mass	Species	Accession	Aa identity	E Value	Alignment length in aa (Start-end position)		
					BpV1_089c	RCC1105 virus BpV1						
					hypothetical protein	Verrucomicrobia bacterium SCGC AAA164-N20	WP_020036351	46.60%	9.86E-30	148(1-148)		
6	4,176	4,391	216	71	hypothetical protein MPWG_00152	Micromonas pusilla virus PL1	AET43639	46.40%	9.23E-11	65(1-65)		transmembrane_regions(tmhmm ,-1.0,9-27)
					hypothetical protein	Verrucomicrobia bacterium SCGC AAA164-N20	WP_020036352	47.50%	1.29E-08	59(2-60)		
7	4,418	5,236	819	272	hypothetical protein MPWG_00151	Micromonas pusilla virus PL1	AET43638	35.70%	2.00E-34	272(1-272)		
8	5,309	5,971	663	220								
9	5,931	6,314	384	127	hypothetical protein OlV1_108c	Ostreococcus lucimarinus virus OlV1	YP_004061741	36.90%	6.25E-19	122(5-126)		
					hypothetical protein	Verrucomicrobia bacterium SCGC AAA164-N20	WP_020036584	38.50%	1.14E-14	115(13-127)		
10	6,346	6,876	531	176								signal-peptide(SignalP-NN(euk),-1.0,1-24)
												transmembrane_regions(tmhmm ,-1.0,5-23)
11	6,943	7,344	402	133	hypothetical protein BpV2_094	Bathycoccus sp. RCC1105 virus BpV2	ADQ91261	45.10%	7.75E-34	129(1-129)		

ID	Position		Length		BlastP hit in GenBank nr and/or viruses data set						NCBI conserved domain (ID, E-value, alignment length in aa, alignment start - end position)	Interproscan matches (ID, E-value, alignment start - end position)
	start	end	nt	aa	ORF, protein encoded, or mass	Species	Accession	Aa identity	E Value	Alignment length in aa (Start-end position)		
12					hypothetical protein	Verrucomicrobia bacterium SCGC AAA164-N20	WP_020036355	44.40%	6.09E-32	129(1-129)		
	7,369	8,109	741	246	PCNA	Ostreococcus tauri virus 2	YP_004063533	53.90%	1.52E-83	244(1-244)	PCNA(cd00577,1.88e-45,242,4-245)	pcna: proliferating cell nuclear antigen (pcna)(TIGR00590,1.6E-49,3-245)
					hypothetical protein	Verrucomicrobia bacterium SCGC AAA164-N20	WP_020036354	54.10%	1.53E-82	244(1-244)	PHA03383(PHA03383,3.51e-81,244,1-244)	
13	8,132	9,040	909	302	hypothetical protein OMVG_00153	Ostreococcus lucimarinus virus OlV3	AFK66153	52.50%	5.03E-91	293(10-302)	Pox_VLTF3(pfam04947,1.16e-56,174,128-301)	Pox_VLTF3(PF04947,4.7E-53,130-300)
14	9,076	9,744	669	222	hypothetical protein PBCVAN69C_686L	Paramecium bursaria Chlorella virus AN69C	AGE48616	39.40%	1.89E-27	184(3-186)	HNHc(cl00083,3.66e-03,40,129-168)	
15	9,704	10,096	393	130	hypothetical protein BpV1_099c	Bathycoccus sp. RCC1105 virus BpV1	YP_004061529	51.50%	7.09E-30	101(4-104)	PDDEXK_3(cl16254,5.47e-31,100,16-115)	PDDEXK_3(PF13366,3.2E-29,10-114)
					hypothetical protein tll0632	Thermosynechococcus elongatus BP-1	NP_681421	42.90%	4.57E-20	101(4-104)		
16	10,155	10,889	735	244								transmembrane_regions(tmhmm ,-1.0,76-96;168-186)
17	10,901	11,323	423	140	hypothetical protein OtV6_119c	Ostreococcus tauri virus RT-2011	AFC35027	42.50%	3.19E-07	78(53-130)		

ID	Position		Length		BlastP hit in GenBank nr and/or viruses data set						NCBI conserved domain (ID, E-value, alignment length in aa, alignment start - end position)	Interproscan matches (ID, E-value, alignment start - end position)
	start	end	nt	aa	ORF, protein encoded, or mass	Species	Accession	Aa identity	E Value	Alignment length in aa (Start-end position)		
18	11,336	12,130	795	264	hypothetical protein MPXG_00049	Micromonas pusilla virus SP1	AET84847	36.40%	3.78E-39	257(2-258)	Patatin_and_cPLA2(cl11396,2.93e-09,127,59-185)	FabD/lysophospholipase-like(SSF52151,5.9E-23,1-244)
					patatin-like protein phospholipase family protein	uncultured bacterium	EKD54247	23.90%	5.75E-12	244(15-258)		Patatin(PF01734,3.1E-13,10-186)
19												signal-peptide(SignalP-NN(euk),-1.0,1-13)
	12,143	12,514	372	123	hypothetical protein BpV1_114c	Bathycoccus sp. RCC1105 virus BpV1	YP_004061544	51.60%	7.10E-20	121(3-123)		signal-peptide(SignalP-NN(euk),-1.0,1-52)
												transmembrane_regions(tmhmm ,-1.0,9-27;37-55;65-85;91-113)
20	12,521	12,913	393	130								
21	12,910	13,257	348	115	hypothetical protein MpV1_128c	Micromonas sp. RCC1109 virus MpV1	YP_004062011	41.10%	7.35E-15	112(2-113)		
22	13,335	13,811	477	158	hypothetical protein OtV6_125c	Ostreococcus tauri virus RT-2011	AFC35033	53.40%	4.16E-25	88(61-148)		signal-peptide(SignalP-NN(euk),-1.0,1-26)
					hypothetical protein	Marine Group II euryarchaeote SCGC AB-629-J06	WP_018035657	39.80%	1.75E-04	86(68-153)		transmembrane_regions(tmhmm ,-1.0,5-20)
23	13,844	14,389	546	181	hypothetical protein	Ostreococcus virus	YP_001648217	36.90%	4.20E-22	168(1-168)		

ID	Position		Length		BlastP hit in GenBank nr and/or viruses data set						NCBI conserved domain (ID, E-value, alignment length in aa, alignment start - end position)	Interproscan matches (ID, E-value, alignment start - end position)
	start	end	nt	aa	ORF, protein encoded, or mass	Species	Accession	Aa identity	E Value	Alignment length in aa (Start-end position)		
					OsV5_140r	OsV5						
24	14,423	14,803	381	126								transmembrane_regions(tmhmm ,-1.0,105-125)
25	14,835	15,302	468	155	hypothetical protein OlV1_150c	Ostreococcus lucimarinus virus OlV1	YP_004061783	57.40%	5.04E-32	119(9-127)	SWIB(cl02489,1.90e-05,79,47-125)	SWIB/MDM2 domain(SSF47592,3.7E-10,38-135)
					hypothetical protein	Verrucomicrobia bacterium SCGC AB-629-E09	WP_018003588	27.70%	7.27E-06	115(12-126)		
26	15,386	16,150	765	254	hypothetical protein OtV6_130	Ostreococcus tauri virus RT-2011	AFC35038	58.60%	2.38E-86	215(12-226)	RNaseIII(TIGR02191,2.66e-61,22-226)	RNaseIII: ribonuclease III(TIGR02191,3.5E-55,20-226)
27	16,089	16,838	750	249	hypothetical protein BpV2_126	Bathycoccus sp. RCC1105 virus BpV2	ADQ91293	52.90%	5.68E-58	189(21-209)	YqaJ(cl09232,1.46e-33,125,29-153)	Restriction endonuclease-like(SSF52980,4.6E-46,5-217)
28	16,985	17,458	474	157								transmembrane_regions(tmhmm ,-1.0,137-155)

ID	Position		Length		BlastP hit in GenBank nr and/or viruses data set						NCBI conserved domain (ID, E-value, alignment length in aa, alignment start - end position)	Interproscan matches (ID, E-value, alignment start - end position)
	start	end	nt	aa	ORF, protein encoded, or mass	Species	Accession	Aa identity	E Value	Alignment length in aa (Start-end position)		
29	17,451	17,768	318	105	hypothetical protein OtV6_138	Ostreococcus tauri virus RT-2011	AFC35046	69.40%	1.41E-40	98(1-98)	NTP-PPase(cl16941,3.85e-11,74,11-84)	Predicted pyrophosphohydrolase, MazG-related, Bacilli type(PIRSF036521,2.9E-6,1-102)
					hypothetical protein	Flexithrix dorotheae	WP_020533483	30.30%	2.89E-09	99(1-99)		all-alpha NTP pyrophosphatases(SSF101386,5.1E-15,1-96)
30	17,792	18,019	228	75								signal-peptide(SignalP-NN(euk),-1.0,1-20)
31	18,228	18,611	384	127	hypothetical protein OtV2_125	Ostreococcus tauri virus 2	YP_004063558	45.50%	7.48E-26	123(4-126)		
32	18,650	20,926	2277	758	ribonucleoside-diphosphate reductase	Algoriphagus sp. PR1	WP_008202901	55.50%	0	756(1-756)	PLN02437(PLN02437,0,757,1-757)	RIBONUCLEOSIDE-DIPHOSPHATE REDUCTASE LARGE CHAIN(PTHR11573,0.0,1-756)
					hypothetical protein OlV1_143	Ostreococcus lucimarinus virus OlV1	YP_004061776	63.00%	0	746(1-746)	RNR_PFL(cl09939,0,571,152-722)	
33	20,947	21,381	435	144								
34	21,353	21,910	558	185								

ID	Position		Length		BlastP hit in GenBank nr and/or viruses data set						NCBI conserved domain (ID, E-value, alignment length in aa, alignment start - end position)	Interproscan matches (ID, E-value, alignment start - end position)
	start	end	nt	aa	ORF, protein encoded, or mass	Species	Accession	Aa identity	E Value	Alignment length in aa (Start-end position)		
35	21,941	22,471	531	176	hypothetical protein OlV1_144	Ostreococcus lucimarinus virus OlV1	YP_004061777	26.30%	1.00E-05	121(27-147)		
36	22,530	22,859	330	109								
37	22,979	23,197	219	72								signal-peptide(SignalP-NN(euk),-1.0,1-22)
												transmembrane_regions(tmhmm ,-1.0,5-27;46-66)
38	23,205	23,447	243	80	hypothetical protein MPXG_00060	Micromonas pusilla virus SP1	AET84858	56.10%	8.09E-05	59(3-61)		
					EsV-1-52	Ectocarpus siliculosus	CBN80356	56.80%	5.49E-02	43(3-45)		
39	23,487	23,984	498	165	GNAT family acetyltransferase	Streptococcus henryi	WP_018164889	31.30%	1.19E-08	82(44-125)	NAT_SF(cl17182,7.15e-07,57,51-107)	Acyl-CoA N-acyltransferases (Nat)(SSF55729,8.0E-15,24-132)
40	24,327	24,860	534	177	hypothetical protein OMVG_00253	Ostreococcus lucimarinus virus OlV3	AFK66247	43.80%	2.72E-25	121(3-123)	GIY-YIG_SF(cl15257,3.03e-09,55,5-59)	zf-CCHC(PF00098,2.2E-6,103-118;1.5E-4,159-174)
					nucleic acid binding protein / zinc ion binding protein	Galdieria sulphuraria	EME30645	34.20%	2.23E-24	171(5-175)	PTZ00368(PTZ00368,6.21e-06,85,89-173)	GIY-YIG(PF01541,9.3E-9,5-70)
41	24,909	25,070	162	53	hypothetical protein PGCG_00053	Phaeocystis globosa virus	YP_008052372	59.60%	3.05E-09	52(1-52)		

ID	Position		Length		BlastP hit in GenBank nr and/or viruses data set						NCBI conserved domain (ID, E-value, alignment length in aa, alignment start - end position)	Interproscan matches (ID, E-value, alignment start - end position)
	start	end	nt	aa	ORF, protein encoded, or mass	Species	Accession	Aa identity	E Value	Alignment length in aa (Start-end position)		
42	25,071	25,235	165	54								signal-peptide(SignalP-NN(euk),-1.0,1-20)<br>transmembrane_regions(tmhmm ,-1.0,5-23;29-47)
43	25,265	26,170	906	301	rieske (2Fe-2S) domain protein	Nostoc flagelliforme str. Sunitezuoqi	ADO19015	26.40%	1.30E-22	266(4-269)	Rieske(cl00938,7.09e-18,112,4-115)<br>HcaE(COG4638,5.95e-27,293,3-295)	IRON-SULFUR DOMAIN CONTAINING PROTEIN(PTHR21266,3.3E-19,3-154)
44	26,163	26,546	384	127								
45	26,556	26,939	384	127								
46	26,965	27,189	225	74								
47	27,214	27,444	231	76								
48	27,452	27,802	351	116							NAT_SF(cl17182,6.01e-09,69,22-90)	Acyl-CoA N-acyltransferase(3.40.630.30,1.2E-4,23-74)
49	27,827	28,135	309	102								
50	28,289	28,453	165	54								transmembrane_regions(tmhmm ,-1.0,21-43)
51	28,484	29,029	546	181								

ID	Position		Length		BlastP hit in GenBank nr and/or viruses data set						NCBI conserved domain (ID, E-value, alignment length in aa, alignment start - end position)	Interproscan matches (ID, E-value, alignment start - end position)
	start	end	nt	aa	ORF, protein encoded, or mass	Species	Accession	Aa identity	E Value	Alignment length in aa (Start-end position)		
52	29,007	29,243	237	78							COG3956(COG3956,6.26e-04,47,32-78)	
53	29,257	29,436	180	59								
54	29,437	29,625	189	62								
55	29,897	30,619	723	240	hypothetical protein	Vibrio cyclitrophicus	WP_016789112	24.20%	3.80E-02	141(55-195)	DUF4231(pfam14015,1.97e-04,88,59-146)	DUF4231(PF14015,4.7E-7,60-146)
												transmembrane_regions(tmhmm ,-1.0,89-109)
56	30,547	31,635	1089	362	ornithine decarboxylase 1	Chlamydomonas reinhardtii	XP_001697502	42.20%	4.83E-93	338(16-353)	PLPDE_III(cl00261,2.10e-139,335,18-352)	ARGININE/DIAMINOPIMELATE/ORNITHINE DECARBOXYLASE(PTHR11482,2.6E-112,18-362)
											LysA(COG0019，3.55e-64，333，20-352）	
57	31,881	32,471	591	196								
58	32,404	33,087	684	227								transmembrane_regions(tmhmm ,-1.0,32-54;69-89)
59	33,252	33,533	282	93	hypothetical protein PBCVMA1D_454L	Paramecium bursaria Chlorella virus MA-1D	AGE54931	60.20%	6.21E-24	93(1-93)		
60	33,627	34,307	681	226								

ID	Position		Length		BlastP hit in GenBank nr and/or viruses data set						NCBI conserved domain (ID, E-value, alignment length in aa, alignment start - end position)	Interproscan matches (ID, E-value, alignment start - end position)
	start	end	nt	aa	ORF, protein encoded, or mass	Species	Accession	Aa identity	E Value	Alignment length in aa (Start-end position)		
61	34,675	34,854	180	59								transmembrane_regions(tmhmm ,-1.0,34-54)
62	34,853	35,854	1002	333	hypothetical protein OlV1_184c	Ostreococcus lucimarinus virus OlV1	YP_004061816	31.20%	4.51E-35	304(23-326)	RFC1(pfam08519,4.41e-03,116,217-332)	DNA polymerase III clamp loader subunits, C-terminal domain(SSF48019,7.4E-6,171-287)
63	35,896	36,903	1008	335	putative type II DNA methyltransferase	Organic Lake phycodnavirus 1	ADX05980	56.30%	4.89E-99	244(90-333)	AdoMet_MTases(cl17173,5.57e-05,81,93-173)	S-adenosyl-L-methionine-dependent methyltransferases(SSF53335,1.0E-7,65-172)
					restriction endonuclease subunit M	Campylobacter upsaliensis	WP_004278076	41.80%	1.34E-85	333(3-335)		
64	36,878	37,447	570	189	hypothetical protein	Campylobacter upsaliensis	WP_004278077	31.60%	1.26E-10	132(28-159)		
					hypothetical protein 162313455	Organic Lake phycodnavirus 1	ADX05979	33.60%	1.95E-04	110(20-129)		
65	37,488	38,069	582	193	hypothetical protein MPVG_00171	Micromonas pusilla virus 12T	YP_007676236	42.90%	6.60E-44	177(15-191)	Clp_protease_like(cl15763,1.73e-23,131,61-191)	CLP_protease(PF00574,2.1E-26,56-191)
					ATP-dependent Clp protease proteolytic subunit	Clostridium sp. HGF2	WP_002605985	35.50%	1.47E-14	136(56-191)		ClpP/crotonase(SSF52096,1.5E-27,61-192)

ID	Position		Length		BlastP hit in GenBank nr and/or viruses data set						NCBI conserved domain (ID, E-value, alignment length in aa, alignment start - end position)	Interproscan matches (ID, E-value, alignment start - end position)
	start	end	nt	aa	ORF, protein encoded, or mass	Species	Accession	Aa identity	E Value	Alignment length in aa (Start-end position)		
66	38,136	38,489	354	117								signal-peptide(SignalP-NN(euk),-1.0,1-3)
												transmembrane_regions(tmhmm ,-1.0,51-71)
67	38,462	39,421	960	319	transcription initiation factor IIB	Micromonas pusilla virus PL1	AET43585	44.50%	5.80E-77	310(7-316)	SUA7(COG1405,1.34e-21,259,47-305)	TRANSCRIPTION INITIATION FACTOR IIB-RELATED(PTHR11618,1.8E-30,49-301)
					general transcription factor TFIIB	Ogataea parapolymorpha DL-1	EFW96389	27.80%	1.29E-18	260(22-281)		
68	39,434	40,177	744	247	hypothetical protein MpV1_158	Micromonas sp. RCC1109 virus MpV1	YP_004062041	61.40%	1.64E-109	241(7-247)		signal-peptide(SignalP-NN(euk),-1.0,1-15)
												transmembrane_regions(tmhmm ,-1.0,5-23)
												Nucleotide-diphospho-sugar transferases(SSF53448,7.6E-7,53-236)
69	40,194	40,829	636	211	hypothetical protein OtV6_160	Ostreococcus tauri virus RT-2011	AFC35068	38.70%	1.22E-39	199(9-207)		

ID	Position		Length		BlastP hit in GenBank nr and/or viruses data set						NCBI conserved domain (ID, E-value, alignment length in aa, alignment start - end position)	Interproscan matches (ID, E-value, alignment start - end position)
	start	end	nt	aa	ORF, protein encoded, or mass	Species	Accession	Aa identity	E Value	Alignment length in aa (Start-end position)		
70	40,919	41,749	831	276	hypothetical protein H665_p156	Ostreococcus tauri virus 1	YP_003212979	44.90%	5.58E-68	270(5-274)	Adenylation_DNA_ligase_like(cl12015,2.44e-41,170,18-187)	MRNA-CAPPING ENZYME(PTHR10367,8.2E-35,17-274)
					mRNA capping enzyme family protein (ISS), partial	Ostreococcus tauri	XP_003075327	32.20%	6.39E-28	259(16-274)	mRNA_cap_C(pfam03919,4.22e-05,81,192-272)	
71	41,831	42,523	693	230	hypothetical protein OLNG_00095	Ostreococcus lucimarinus virus OlV5	YP_007674737	35.10%	1.86E-29	188(1-188)	Peptidase_C19(cl02553,4.73e-13,111,3-113)	UCH_2_3(PS50235,11.627,2-230)
					PREDICTED: ubiquitin carboxyl-terminal hydrolase 33-like	Meleagris gallopavo	XP_003208801	34.60%	2.69E-12	129(1-129)	UCH(pfam00443,4.08e-23,202,2-203)	Cysteine proteinases(SSF54001,1.3E-28,1-199)
72	42,553	43,170	618	205	hypothetical protein BpV2_156	Bathycoccus sp. RCC1105 virus BpV2	ADQ91323	38.90%	2.17E-36	183(20-202)	CYTH-like_Pase(cl11964,3.79e-14,118,85-202)	mRNA_triPase(PF02940,4.2E-12,45-179)
					hypothetical protein SCHCODRAFT_233597	Schizophyllum commune H4-8	XP_003034557	26.90%	4.62E-02	115(85-199)		CYTH-like phosphatases(SSF55154,7.2E-12,32-205)
73	43,167	45,695	2529	842								Beta2-adaptin/TBP, C-terminal domain(3.30.310.10,9.8E-4,148-216)
74	45,714	47,639	1926	641	hypothetical protein OlV1_165c	Ostreococcus lucimarinus virus OlV1	YP_004061797	54.40%	0	516(121-636)	primase_Cterm(TIGR01613,2.40e-14,147,370-516)	SF3_HELICASE_1(PS51206,12.854,340-504)

ID	Position		Length		BlastP hit in GenBank nr and/or viruses data set						NCBI conserved domain (ID, E-value, alignment length in aa, alignment start - end position)	Interproscan matches (ID, E-value, alignment start - end position)
	start	end	nt	aa	ORF, protein encoded, or mass	Species	Accession	Aa identity	E Value	Alignment length in aa (Start-end position)		
												D5_N(PF08706,1.2E-4,194-285)<br>P-loop containing nucleoside triphosphate hydrolases(SSF52540,2.2E-12,344-523)
75	47,663	47,974	312	103	hypothetical protein MPWG_00084	Micromonas pusilla virus PL1	AET43573	41.90%	1.03E-04	74(12-85)		
76	48,029	49,471	1443	480	hypothetical protein OtV6_168	Ostreococcus tauri virus RT-2011	AFC35076	51.70%	1.86E-159	478(3-480)	Capsid_NCLDV(pfam04451,6.50e-16,88,205-292;1.59e-34,117,360-476)	Capsid_NCLDV(PF04451,3.7E-58,205-476)<br>Group II dsDNA viruses VP(SSF49749,3.0E-49,26-222;7.7E-45,223-480)<br>African swine fever virus, p72, major capsid(2.70.9.20,2.5E-55,328-480)
77	49,556	50,914	1359	452	hypothetical protein OlV1_167	Ostreococcus lucimarinus virus OlV1	YP_004061799	62.30%	0	452(1-452)	Capsid_NCLDV(pfam04451,4.80e-21,73,201-273;1.51e-34,100,349-448)	Capsid_NCLDV(PF04451,7.2E-66,200-448)

ID	Position		Length		BlastP hit in GenBank nr and/or viruses data set						NCBI conserved domain (ID, E-value, alignment length in aa, alignment start - end position)	Interproscan matches (ID, E-value, alignment start - end position)
	start	end	nt	aa	ORF, protein encoded, or mass	Species	Accession	Aa identity	E Value	Alignment length in aa (Start-end position)		
												Group II dsDNA viruses VP(SSF49749,9.5E-56,25-222;1.9E-44,223-452)
												African swine fever virus, p72, major capsid(2.70.9.20,7.1E-60,344-452)
78	51,016	51,336	321	106	S-adenosylmethionine decarboxylase proenzyme	Desulfobacter postgatei	WP_004074803	41.60%	1.82E-16	77(20-96)	AdoMet_dc(cl00687,6.69e-28,78,19-96)	AdoMet_dc(PF02675,3.6E-25,16-105)
79	51,345	51,527	183	60								signal-peptide(SignalP-NN(euk),-1.0,1-21)
												transmembrane_regions(tmhmm ,-1.0,5-27;37-55)
80	51,548	51,913	366	121								
81	52,087	52,650	564	187	hypothetical protein CHLNCDRAFT_138957	Chlorella variabilis	EFN52536	47.90%	2.80E-06	45(134-178)	RING(cl17238,8.28e-05,42,135-176)	Zinc finger, RING/FYVE/PHD-type(3.30.40.10,3.2E-11,124-181)
											zf-C3HC4_2(pfam13923,9.17e-08,42,133-174)	

ID	Position		Length		BlastP hit in GenBank nr and/or viruses data set						NCBI conserved domain (ID, E-value, alignment length in aa, alignment start - end position)	Interproscan matches (ID, E-value, alignment start - end position)
	start	end	nt	aa	ORF, protein encoded, or mass	Species	Accession	Aa identity	E Value	Alignment length in aa (Start-end position)		
82	53,119	53,793	675	224	hypothetical protein	Mesorhizobium sp. STM 4661	WP_006332065	41.80%	3.11E-23	146(73-218)		
					putative nuclease	Moumouvirus Monve	AEX62994	32.40%	4.42E-20	219(3-221)		
83	53,812	54,624	813	270	FAD-dependent thymidylate synthase	Micromonas pusilla virus SP1	AET85027	57.40%	3.74E-90	246(3-248)	Thy1(cl03630,1.15e-86,213,1-213)	Thymidylate synthase-complementing protein Thy1(SSF69796,1.1E-63,1-215)
					thymidylate synthase	Dictyostelium fasciculatum	XP_004366976	52.10%	1.30E-84	252(2-253)		
84	54,647	54,991	345	114	hypothetical protein	Marine Group II euryarchaeote SCGC AB-629-J06	WP_018035887	49.40%	3.10E-22	87(2-88)	HNHc(cl00083,1.05e-05,51,20-70)	HNH nucleases(SM00507,0.0031,16-69)
					hypothetical protein crov535	Cafeteria roenbergensis virus BV-PW1	YP_003970168	37.20%	3.00E-07	77(2-78)		
85	55,681	56,040	360	119								
86	56,059	56,451	393	130								CRAL-TRIO domain(3.40.525.10,7.5E-4,19-127)
87	56,423	57,136	714	237	conserved hypothetical protein	Culex quinquefasciatus	XP_001846879	35.70%	8.75E-20	156(2-157)	Glyco_transf_25(cl01298,1.89e-18,69,2-70)	Glyco_transf_25(PF01755,5.7E-17,2-97)
88	57,295	57,843	549	182								signal-peptide(SignalP-NN(euk),-1.0,1-1)

ID	Position		Length		BlastP hit in GenBank nr and/or viruses data set						NCBI conserved domain (ID, E-value, alignment length in aa, alignment start - end position)	Interproscan matches (ID, E-value, alignment start - end position)
	start	end	nt	aa	ORF, protein encoded, or mass	Species	Accession	Aa identity	E Value	Alignment length in aa (Start-end position)		
89	57,858	58,610	753	250	hypothetical protein MpV1_186c	Micromonas sp. RCC1109 virus MpV1	YP_004062069	26.80%	2.79E-12	151(3-153)		transmembrane_regions(tmhmm ,-1.0,43-63;69-89)
90	58,881	59,174	294	97								signal-peptide(SignalP-NN(euk),-1.0,1-41)
												transmembrane_regions(tmhmm ,-1.0,22-42)
91	59,191	59,481	291	96	hypothetical protein PANDA_008648, partial	Ailuropoda melanoleuca	EFB28322	55.00%	7.13E-02	40(47-86)		signal-peptide(SignalP-NN(euk),-1.0,1-39)
												transmembrane_regions(tmhmm ,-1.0,24-44)
92	59,496	60,473	978	325	hypothetical protein OtV6_156c	Ostreococcus tauri virus RT-2011	AFC35064	63.00%	4.46E-140	316(10-325)	RNRR2(cd01049,6.54e-112,275,20-294)	RIBONUCLEOSIDE-DIPHOSPHATE REDUCTASE SMALL CHAIN(PTHR23409,1.1E-180,7-325)
					ribonucleoside-diphosphate reductase	Arabidopsis lyrata subsp. lyrata	XP_002870664	59.90%	9.13E-136	316(10-325)		transmembrane_regions(tmhmm ,-1.0,164-184)

ID	Position		Length		BlastP hit in GenBank nr and/or viruses data set						NCBI conserved domain (ID, E-value, alignment length in aa, alignment start - end position)	Interproscan matches (ID, E-value, alignment start - end position)
	start	end	nt	aa	ORF, protein encoded, or mass	Species	Accession	Aa identity	E Value	Alignment length in aa (Start-end position)		
93	60,516	60,815	300	99								signal-peptide(SignalP-NN(euk),-1.0,1-20)
												transmembrane_regions(tmhmm ,-1.0,9-29)
94	60,812	61,075	264	87	hypothetical protein MPVG_00082	Micromonas pusilla virus 12T	YP_007676150	36.00%	3.72E-05	50(11-60)	DUF3671(pfam12420,3.55e-03,64,3-66)	signal-peptide(SignalP-NN(euk),-1.0,1-23)
												transmembrane_regions(tmhmm ,-1.0,10-28;42-60)
95	61,090	61,395	306	101	hypothetical protein OlV1_127c	Ostreococcus lucimarinus virus OlV1	YP_004061760	56.50%	9.90E-10	46(14-59)		signal-peptide(SignalP-NN(euk),-1.0,1-20)
												transmembrane_regions(tmhmm ,-1.0,10-32;42-62)
96	61,429	63,357	1929	642								
97	63,435	64,373	939	312								
98	64,448	65,128	681	226	hypothetical protein MPVG_00083	Micromonas pusilla virus 12T	YP_007676151	45.70%	9.96E-48	203(1-203)		
99	65,175	67,232	2058	685	hypothetical protein OtV6_148c	Ostreococcus tauri virus RT-2011	AFC35056	29.00%	2.13E-13	278(255-532)	PKc_like(cl09925,1.18e-12,184,161-344)	Pkinase(PF00069,3.0E-13,159-344)
					putative protein kinase, fragment,	Trypanosoma vivax Y486	CCC49202	31.40%	4.94E-06	169(161-329)		

ID	Position		Length		BlastP hit in GenBank nr and/or viruses data set						NCBI conserved domain (ID, E-value, alignment length in aa, alignment start - end position)	Interproscan matches (ID, E-value, alignment start - end position)
	start	end	nt	aa	ORF, protein encoded, or mass	Species	Accession	Aa identity	E Value	Alignment length in aa (Start-end position)		
					partial							
100	67,241	67,525	285	94	hypothetical protein H665_p144	Ostreococcus tauri virus 1	YP_003212967	30.10%	1.06E-07	92(3-94)	Thioredoxin_like(cl00388,2.68e-08,59,35-93)	Thioredoxin-like(SSF52833,1.2E-8,34-93)
					hypothetical protein	Methylophilus methylotrophus	WP_018987379	34.00%	1.14E-07	93(1-93)		signal-peptide(SignalP-NN(euk),-1.0,1-25)
												transmembrane_regions(tmhmm ,-1.0,5-27)
101	67,560	67,763	204	67	hypothetical protein MpV1_149	Micromonas sp. RCC1109 virus MpV1	YP_004062032	33.30%	3.38E-06	66(1-66)		signal-peptide(SignalP-NN(euk),-1.0,1-36)
												transmembrane_regions(tmhmm ,-1.0,15-35)
102	67,804	68,670	867	288	hypothetical protein BpV1_139c	Bathycoccus sp. RCC1105 virus BpV1	YP_004061569	38.90%	4.74E-40	242(42-283)	Chalcone(cl03589,6.49e-03,76,174-249)	
					hypothetical protein VOLCADRAFT_119457	Volvox carteri f. nagariensis	XP_002956623	25.00%	1.11E-04	226(60-285)		
103	68,657	69,148	492	163	hypothetical protein OsV5_164f	Ostreococcus virus OsV5	YP_001648241	30.00%	3.05E-12	129(34-162)	RING(cl17238,5.82e-08,47,34-80)	RING/U-box(SSF57850,8.0E-13,27-98)
					uncharacterized protein LOC100303800	Zea mays	NP_001158906	46.30%	1.01E-07	52(32-83)		

ID	Position		Length		BlastP hit in GenBank nr and/or viruses data set						NCBI conserved domain (ID, E-value, alignment length in aa, alignment start - end position)	Interproscan matches (ID, E-value, alignment start - end position)
	start	end	nt	aa	ORF, protein encoded, or mass	Species	Accession	Aa identity	E Value	Alignment length in aa (Start-end position)		
104	69,185	69,520	336	111	PREDICTED: LOW QUALITY PROTEIN: E3 ubiquitin-protein ligase synoviolin	Cavia porcellus	XP_005003028	45.70%	1.50E-08	46(27-72)	RING(cl17238,1.12e-06,41,30-70)	RING/U-box(SSF57850,8.1E-16,24-93)
											zf-C3HC4_2(pfam13923,2.61e-06,44,28-71)	
105	69,414	70,070	657	218	kinase protein	Paramecium bursaria Chlorella virus NE-JV-1	AGE56325	36.80%	1.35E-25	181(1-181)	NK(cl17190,1.37e-31,166,2-167)	Deoxynucleoside kinase(PIRSF000705,1.4E-11,1-189)
					deoxynucleoside kinase	Prevotella sp. CAG:474	CDC97404	35.20%	6.61E-19	165(3-167)	COG1428(COG1428,6.21e-18,182,1-182)	
106	70,328	71,125	798	265	hypothetical protein OMVG_00121	Ostreococcus lucimarinus virus OlV3	AFK66121	50.90%	8.76E-86	262(2-263)	TBP_TLF(cl08263,8.16e-07,167,58-224)	TATA-box binding protein-like(SSF55945,1.5E-11,135-230)
					hypothetical protein GUITHDRAFT_160489	Guillardia theta CCMP2712	EKX55535	27.50%	3.02E-07	126(96-221)		
107	71,176	73,824	2649	882								
108	73,872	75,308	1437	478	hypothetical protein MpV1_136	Micromonas sp. RCC1109 virus MpV1	YP_004062019	56.50%	0	473(4-476)	DEXDc(cl17251,3.57e-15,131,31-161)	helicase superfamily c-terminal domain(SM00490,4.8E-22,324-407)

ID	Position		Length		BlastP hit in GenBank nr and/or viruses data set						NCBI conserved domain (ID, E-value, alignment length in aa, alignment start - end position)	Interproscan matches (ID, E-value, alignment start - end position)
	start	end	nt	aa	ORF, protein encoded, or mass	Species	Accession	Aa identity	E Value	Alignment length in aa (Start-end position)		
											HELICc(cl17351,3.00e-23,121,294-414)	DEAD-like helicases superfamily(SM00487,3.8E-26,5-189)
											SNF2_N(pfam00176,1.98e-52,258,12-269)	SWI/SNF-RELATED MATRIX-ASSOCIATED ACTIN-DEPENDENT REGULATOR OF CHROMATIN SUBFAMILY-RELATED(PTHR10799,5.3E-99,11-476)
											SrmB(COG0513,2.02e-07,117,293-409)	
109	75,315	75,557	243	80	hypothetical protein MPXG_00060	Micromonas pusilla virus SP1	AET84858	53.80%	4.95E-04	58(4-61)		
					EsV-1-52	Ectocarpus siliculosus	CBN80356	58.10%	9.63E-02	42(4-45)		
110	75,565	75,789	225	74								signal-peptide(SignalP-NN(euk),-1.0,1-23)
												transmembrane_regions(tmhmm ,-1.0,10-32;47-67)

ID	Position		Length		BlastP hit in GenBank nr and/or viruses data set						NCBI conserved domain (ID, E-value, alignment length in aa, alignment start - end position)	Interproscan matches (ID, E-value, alignment start - end position)
	start	end	nt	aa	ORF, protein encoded, or mass	Species	Accession	Aa identity	E Value	Alignment length in aa (Start-end position)		
111	75,909	76,265	357	118								
112	76,324	76,854	531	176	hypothetical protein OlV1_144	Ostreococcus lucimarinus virus OlV1	YP_004061777	26.30%	1.09E-05	121(27-147)		
113	76,866	77,462	597	198								
114	77,451	77,879	429	142								
115	77,879	78,049	171	56	hypothetical protein PGCG_00053	Phaeocystis globosa virus	YP_008052372	62.50%	1.14E-11	56(1-56)		
116	78,069	80,354	2286	761	hypothetical protein OtV6_135c	Ostreococcus tauri virus RT-2011	AFC35043	61.70%	0	749(1-749)	ATP-cone(pfam03477,4.86e-07,90,1-90)	RIBONUCLEOSIDE-DIPHOSPHATE REDUCTASE LARGE CHAIN(PTHR11573,0.0,1-759)
					hypothetical protein	Monosiga brevicollis MX1	XP_001746543	55.60%	0	761(1-761)	RNR_PFL(cl09939,0,571,152-722)	PFL-like glycyl radical enzymes(SSF51998,6.1E-185,200-733)
											PLN02437(PLN02437,0,760,1-760)	
117	80,394	80,777	384	127	hypothetical protein OLNG_00118	Ostreococcus lucimarinus virus OlV5	YP_007674760	47.20%	1.51E-26	123(4-126)		
118	80,778	81,029	252	83								signal-peptide(SignalP-NN(euk),-1.0,1-32)

ID	Position		Length		BlastP hit in GenBank nr and/or viruses data set						NCBI conserved domain (ID, E-value, alignment length in aa, alignment start - end position)	Interproscan matches (ID, E-value, alignment start - end position)
	start	end	nt	aa	ORF, protein encoded, or mass	Species	Accession	Aa identity	E Value	Alignment length in aa (Start-end position)		
119	81,053	81,280	228	75								transmembrane_regions(tmhmm ,-1.0,10-30;59-79)<br>signal-peptide(SignalP-NN(euk),-1.0,1-20)<br>transmembrane_regions(tmhmm ,-1.0,9-29;43-63)
120	81,304	81,621	318	105	hypothetical protein OtV6_138	Ostreococcus tauri virus RT-2011	AFC35046	69.40%	3.30E-40	98(1-98)	NTP-PPase(cl16941,6.38e-11,73,11-83)	MazG(PF03819,1.5E-6,29-95)
					hypothetical protein	Flexithrix dorotheae	WP_020533483	31.30%	3.09E-10	99(1-99)		all-alpha NTP pyrophosphatases(SSF101386,1.6E-14,1-96)
121	81,614	82,087	474	157								transmembrane_regions(tmhmm ,-1.0,137-155)
122	82,068	82,292	225	74								Zinc finger, AN1-type(4.10.1110.10,2.4E-4,17-70)
123	82,246	82,983	738	245	hypothetical protein MPWG_00120	Micromonas pusilla virus PL1	AET43608	53.60%	1.57E-57	191(15-205)	YqaJ(cl09232,1.06e-31,125,29-153)	Restriction endonuclease-like(SSF52980,1.2E-45,5-203)

ID	Position		Length		BlastP hit in GenBank nr and/or viruses data set						NCBI conserved domain (ID, E-value, alignment length in aa, alignment start - end position)	Interproscan matches (ID, E-value, alignment start - end position)
	start	end	nt	aa	ORF, protein encoded, or mass	Species	Accession	Aa identity	E Value	Alignment length in aa (Start-end position)		
124	82,922	83,686	765	254	hypothetical protein OtV6_130	Ostreococcus tauri virus RT-2011	AFC35038	57.70%	9.14E-85	215(12-226)	RIBOc(cl00258,3.81e-39,128,29-156)	RIBONUCLEASE III(PTHR11207,7.8E-43,26-226)
											DSRM(cl00054,1.26e-14,62,165-226)	
											RNaseIII(TIGR02191,1.32e-62,205,22-226)	
125	83,796	84,272	477	158	hypothetical protein MPXG_00056	Micromonas pusilla virus SP1	AET84854	55.80%	1.85E-30	117(11-127)		
					hypothetical protein	Verrucomicrobia bacterium SCGC AB-629-E09	WP_018003588	27.70%	3.06E-05	115(12-126)	SWIB(cl02489,5.57e-07,79,47-125)	SWIB/MDM2 domain(SSF47592,4.0E-11,38-135)
126	84,304	84,690	387	128								transmembrane_regions(tmhmm ,-1.0,107-127)
127	84,724	85,269	546	181	hypothetical protein OsV5_140r	Ostreococcus virus OsV5	YP_001648217	37.50%	4.66E-23	168(1-168)		
128	85,302	85,775	474	157	hypothetical protein OtV6_125c	Ostreococcus tauri virus RT-2011	AFC35033	53.30%	2.73E-25	90(58-147)		signal-peptide(SignalP-NN(euk),-1.0,1-26)
					hypothetical protein	Marine Group II euryarchaeote SCGC AB-629-J06	WP_018035657	39.80%	1.71E-04	86(67-152)		transmembrane_regions(tmhmm ,-1.0,5-20)
129	85,840	86,187	348	115	hypothetical protein OtV6_124c	Ostreococcus tauri virus RT-2011	AFC35032	42.00%	1.12E-15	112(2-113)		
130	86,447	86,932	486	161								

ID	Position		Length		BlastP hit in GenBank nr and/or viruses data set						NCBI conserved domain (ID, E-value, alignment length in aa, alignment start - end position)	Interproscan matches (ID, E-value, alignment start - end position)
	start	end	nt	aa	ORF, protein encoded, or mass	Species	Accession	Aa identity	E Value	Alignment length in aa (Start-end position)		
131	86,973	87,767	795	264	hypothetical protein MPXG_00049	Micromonas pusilla virus SP1	AET84847	37.40%	1.53E-39	257(2-258)	Patatin_and_cPLA2(cl11396,7.32e-09,117,59-175)	Patatin(PF01734,2.5E-12,10-181)
					patatin-like protein phospholipase family protein	uncultured bacterium	EKD54247	23.60%	5.63E-11	244(15-258)		FabD/lysophospholipase-like(SSF52151,1.7E-21,1-186)
132	87,780	88,196	417	138	hypothetical protein OtV6_119c	Ostreococcus tauri virus RT-2011	AFC35027	42.00%	8.28E-06	67(64-130)		
133	88,193	88,522	330	109								
134	88,586	89,890	1305	434								transmembrane_regions(tmhmm ,-1.0,59-79)
135	89,944	90,333	390	129	hypothetical protein BpV1_099c	Bathycoccus sp. RCC1105 virus BpV1	YP_004061529	53.50%	5.18E-30	101(4-104)	PDDEXK_3(cl16254,2.08e-28,88,16-103)	PDDEXK_3(PF13366,3.1E-29,7-108)
					hypothetical protein	candidate division DUSEL4 archaeon SCGC AAA011-L22	WP_018203135	43.70%	1.02E-19	99(6-104)		
136	90,335	91,243	909	302	hypothetical protein OMVG_00153	Ostreococcus lucimarinus virus OlV3	AFK66153	52.20%	2.17E-90	293(10-302)	Pox_VLTF3(pfam04947,1.84e-56,74,128-301)	Pox_VLTF3(PF04947,4.0E-53,130-300)
137	91,267	92,007	741	246	hypothetical protein BpV2_095	Bathycoccus sp. RCC1105 virus BpV2	ADQ91262	55.30%	7.93E-84	244(1-244)	PCNA(cd00577,8.07e-44,242,4-245)	PROLIFERATING CELL NUCLEAR ANTIGEN(PTHR11352,5.9E-50,6-245)
					hypothetical protein	Verrucomicrobia bacterium SCGC	WP_020036354	54.50%	8.71E-83	244(1-244)	PHA03383(PHA03383,2.46e-80,244,1-244)	

ID	Position		Length		BlastP hit in GenBank nr and/or viruses data set						NCBI conserved domain (ID, E-value, alignment length in aa, alignment start - end position)	Interproscan matches (ID, E-value, alignment start - end position)
	start	end	nt	aa	ORF, protein encoded, or mass	Species	Accession	Aa identity	E Value	Alignment length in aa (Start-end position)		
						AAA164-N20						
138	92,032	92,433	402	133	hypothetical protein MPVG_00121	Micromonas pusilla virus 12T	YP_007676187	47.80%	8.36E-35	127(1-127)		
139	92,499	93,029	531	176	bacteriocin ABC transporter putative	Clostridium perfringens SM101	YP_697983	31.20%	3.41E-02	75(1-75)		signal-peptide(SignalP-NN(euk),-1.0,1-24)
												transmembrane_regions(tmhmm ,-1.0,5-23)
140	93,061	93,453	393	130	hypothetical protein OlV1_108c	Ostreococcus lucimarinus virus OlV1	YP_004061741	34.70%	5.66E-18	121(5-125)		
					hypothetical protein	Verrucomicrobia bacterium SCGC AAA164-N20	WP_020036584	32.50%	4.75E-14	121(5-125)		
141	93,474	94,280	807	268	hypothetical protein BpV2_091c	Bathycoccus sp. RCC1105 virus BpV2	ADQ91258	37.00%	1.64E-35	230(1-230)		
142	94,307	94,522	216	71	hypothetical protein MPWG_00152	Micromonas pusilla virus PL1	AET43639	46.40%	1.50E-10	65(1-65)		transmembrane_regions(tmhmm ,-1.0,9-27)
					hypothetical protein	Verrucomicrobia bacterium SCGC AAA164-N20	WP_020036352	47.50%	1.06E-08	59(2-60)		
143	94,553	95,098	546	181	hypothetical protein BpV1_089c	Bathycoccus sp. RCC1105 virus BpV1	YP_004061519	45.30%	5.51E-30	148(1-148)		

ID	Position		Length		BlastP hit in GenBank nr and/or viruses data set						NCBI conserved domain (ID, E-value, alignment length in aa, alignment start - end position)	Interproscan matches (ID, E-value, alignment start - end position)
	start	end	nt	aa	ORF, protein encoded, or mass	Species	Accession	Aa identity	E Value	Alignment length in aa (Start-end position)		
					hypothetical protein	Verrucomicrobia bacterium SCGC AAA164-N20	WP_020036351	45.30%	8.51E-30	148(1-148)		
144	95,071	96,039	969	322								
	96,030	97,133	1104	367	hypothetical protein MpV1_098c	Micromonas sp. RCC1109 virus MpV1	YP_004061981	30.20%	1.08E-28	359(2-360)		signal-peptide(SignalP-NN(euk),-1.0,1-15)
145												
					hypothetical protein	Verrucomicrobia bacterium SCGC AAA164-N20	WP_020036348	28.70%	4.79E-20	352(8-359)		
	97,156	98,331	1176	391	hypothetical protein MPVG_00151	Micromonas pusilla virus 12T	YP_007676216	42.90%	2.21E-92	391(1-391)	Capsid_NCLDV(pfam04451,5.49e-22,188,200-387)	Capsid_NCLDV(PF04451,4.6E-21,200-387)
					hypothetical protein	Verrucomicrobia bacterium SCGC AAA164-N20	WP_020036256	39.60%	3.84E-82	391(1-391)		Group II dsDNA viruses VP(SSF49749,1.2E-37,25-217;8.2E-13,218-391)
146												
												African swine fever virus, p72, major capsid(2.70.9.20,1.9E-18,248-391)
147	98,369	99,610	1242	413	hypothetical protein COCSUDRAFT_64687	Coccomyxa subellipsoidea C-169	EIE26852	40.90%	1.52E-46	217(55-271)		
148	99,553	100,425	873	290								
149	100,594	100,863	270	89							PRK06943(PRK06943,9.69e-03,24,54-77)	

ID	Position		Length		BlastP hit in GenBank nr and/or viruses data set						NCBI conserved domain (ID, E-value, alignment length in aa, alignment start - end position)	Interproscan matches (ID, E-value, alignment start - end position)
	start	end	nt	aa	ORF, protein encoded, or mass	Species	Accession	Aa identity	E Value	Alignment length in aa (Start-end position)		
150	100,886	101,956	1071	356	hypothetical protein MpV1_083	Micromonas sp. RCC1109 virus MpV1	YP_004061966	35.90%	1.07E-51	282(12-293)	Herpes_UL52 super family(cl17300,1.98e-03,39,238-276)	Herpes_UL52(PF03121,4.4E-8,239-276)
					hypothetical protein	Verrucomicrobia bacterium SCGC AAA164-N20	WP_020036261	35.70%	2.16E-48	278(16-293)		
151	102,175	102,564	390	129								
152	102,788	103,066	279	92								signal-peptide(SignalP-NN(euk),-1.0,1-27)
												transmembrane_regions(tmhmm ,-1.0,5-23)
153	103,088	103,705	618	205	hypothetical protein OtV2_075	Ostreococcus tauri virus 2	YP_004063508	62.60%	1.76E-68	199(3-201)	PRK13435（PRK13435,1.92e-04,43,25-67)	
					hypothetical protein	Verrucomicrobia bacterium SCGC AAA164-N20	WP_020036266	64.40%	2.20E-63	177(1-177)	caca（TIGR00845,4.99e-03,24,150-173)	
154	103,739	104,407	669	222	hypothetical protein BpV1_072	Bathycoccus sp. RCC1105 virus BpV1	YP_004061502	43.20%	9.32E-52	220(1-220)	AdoMet_MTases(cd02440,3.63e-06,100,26-125)	S-adenosyl-L-methionine-dependent methyltransferases(SSF53335,3.2E-23,9-220)
					hypothetical protein	Verrucomicrobia bacterium SCGC AAA164-N20	WP_020036272	42.30%	4.92E-50	220(1-220)		

ID	Position		Length		BlastP hit in GenBank nr and/or viruses data set						NCBI conserved domain (ID, E-value, alignment length in aa, alignment start - end position)	Interproscan matches (ID, E-value, alignment start - end position)
	start	end	nt	aa	ORF, protein encoded, or mass	Species	Accession	Aa identity	E Value	Alignment length in aa (Start-end position)		
155	104,326	104,844	519	172	hypothetical protein MPXG_00018	Micromonas pusilla virus SP1	AET84816	44.60%	8.03E-33	138(34-171)	WLM super family(cl07077,1.68e-03,53,96-148)	transmembrane_regions(tmhmm ,-1.0,26-46)
					hypothetical protein	Verrucomicrobia bacterium SCGC AAA164-N20	WP_020036273	40.80%	3.63E-25	124(48-171)		WLM(PF08325,3.4E-6,94-150)
156	104,915	105,421	507	168	hypothetical protein BpV1_077c	Bathycoccus sp. RCC1105 virus BpV1	YP_004061507	39.10%	3.55E-18	127(37-163)		transmembrane_regions(tmhmm ,-1.0,105-123;142-160)
157	105,529	106,467	939	312	hypothetical protein MPVG_00136	Micromonas pusilla virus 12T	YP_007676201	64.40%	7.74E-128	259(52-310)		
					hypothetical protein	Verrucomicrobia bacterium SCGC AAA164-N20	WP_020036342	67.50%	1.19E-126	236(52-287)	AAA(smart00382,5.35e-03,117,68-185)	no description(3.40.50.300,2.0E-5,68-186)
158	106,419	108,401	1983	660	hypothetical protein MPWG_00161	Micromonas pusilla virus PL1	AET43648	45.10%	3.82E-11	99(3-101)		
					hypothetical protein	Verrucomicrobia bacterium SCGC AAA164-N20	WP_020036343	50.60%	7.27E-10	76(3-78)		Group II dsDNA viruses VP(SSF49749,4.0E-8,25-82)
159	108,898	109,215	318	105	major capsid protein	Ostreococcus tauri virus 2	YP_004063519	36.60%	3.31E-11	99(1-99)	Capsid_NCLDV(pfam04451,1.63e-18,96,1-96)	Capsid_NCLDV(PF04451,8.7E-21,2-96)
												Group II dsDNA viruses VP(SSF49749,7.1E-20,1-100)

ID	Position		Length		BlastP hit in GenBank nr and/or viruses data set						NCBI conserved domain (ID, E-value, alignment length in aa, alignment start - end position)	Interproscan matches (ID, E-value, alignment start - end position)
	start	end	nt	aa	ORF, protein encoded, or mass	Species	Accession	Aa identity	E Value	Alignment length in aa (Start-end position)		
160	109,293	110,480	1188	395	hypothetical protein MPXG_00023	Micromonas pusilla virus SP1	AET84821	31.10%	9.79E-44	383(1-383)	Capsid_NCLDV(pfam04451,1.85e-21,285,199-383)	African swine fever virus, p72, major capsid(2.70.9.20,2.0E-22,2-99) Capsid_NCLDV(PF04451,9.2E-28,199-384)
					hypothetical protein	Verrucomicrobia bacterium SCGC AAA164-N20	WP_020036343	29.50%	3.05E-29	383(1-383)		Group II dsDNA viruses VP(SSF49749,1.4E-25,24-217;1.2E-20,218-391) African swine fever virus, p72, major capsid(2.70.9.20,4.6E-26,224-390)
161	110,508	110,891	384	127	hypothetical protein H665_p095	Ostreococcus tauri virus 1	YP_003212918	49.10%	2.22E-23	116(8-123)		
					hypothetical protein	Verrucomicrobia bacterium SCGC AAA164-N20	WP_020036345	48.30%	7.76E-21	117(7-123)		
162	110,987	111,394	408	135	hypothetical protein MPXG_00025	Micromonas pusilla virus SP1	AET84823	58.00%	6.13E-23	88(46-133)		transmembrane_regions(tmhmm ,-1.0,61-81;95-113)

ID	Position		Length		BlastP hit in GenBank nr and/or viruses data set						NCBI conserved domain (ID, E-value, alignment length in aa, alignment start - end position)	Interproscan matches (ID, E-value, alignment start - end position)
	start	end	nt	aa	ORF, protein encoded, or mass	Species	Accession	Aa identity	E Value	Alignment length in aa (Start-end position)		
163	111,411	112,424	1014	337	hypothetical protein	Verrucomicrobia bacterium SCGC AAA164-N20	WP_020036346	52.00%	4.76E-17	99(35-133)		transmembrane_regions(tmhmm ,-1.0,302-322)
					hypothetical protein BpV2_085c	Bathycoccus sp. RCC1105 virus BpV2	ADQ91252	35.70%	1.28E-07	110(1-110)		
					hypothetical protein	Verrucomicrobia bacterium SCGC AAA164-N20	WP_020036347	32.40%	6.62E-05	138(1-138)		
164	112,443	112,751	309	102								
165	112,756	114,213	1458	485	hypothetical protein ACD_78C00046G0001, partial	uncultured bacterium (gcode 4)	EKD30474	39.10%	2.71E-13	110(355-464)	Peptidase_S74(pfam13884,1.65e-15,46,348-393)	HYPOTHETICAL PROTEIN(PTHR13029:SF12,1.4E-5,348-406)
					hypothetical protein MpV1_073	Micromonas sp. RCC1109 virus MpV1	YP_004061956	38.60%	3.97E-13	131(299-429)		Peptidase_S74(PF13884,4.4E-19,348-401)
166	114,228	118,256	4029	1342								NHL repeat(SSF101898,5.8E-12,1015-1281)
167	118,281	122,579	4299	1432								NHL repeat(SSF101898,2.1E-9,1107-1423)
168	122,643	123,041	399	132								
169	122,983	124,761	1779	592	hypothetical protein	Clostridium sp. JC122	WP_010291791	29.50%	1.83E-27	438(153-590)		
					hypothetical protein SXDG_00036	Synechococcus phage S-RIM8 A.HR1	YP_007518185	25.90%	3.77E-04	172(178-349)		
170	124,856	125,194	339	112	hypothetical protein	Methylobacterium extorquens	WP_003600553	26.20%	3.55E-02	101(7-107)		

ID	Position		Length		BlastP hit in GenBank nr and/or viruses data set						NCBI conserved domain (ID, E-value, alignment length in aa, alignment start - end position)	Interproscan matches (ID, E-value, alignment start - end position)
	start	end	nt	aa	ORF, protein encoded, or mass	Species	Accession	Aa identity	E Value	Alignment length in aa (Start-end position)		
171	125,221	126,165	945	314	hypothetical protein OSG_eHP7_00185, partial	Environmental Halophage eHP-7	AFH21718	33.60%	3.94E-03	104(46-149)		Concanavalin A-like lectins/glucanases(SSF49899, 9.7E-14,105-313)
172	126,162	128,846	2685	894								
173	128,839	129,480	642	213	hypothetical protein OlV1_072	Ostreococcus lucimarinus virus OlV1	YP_004061705	28.70%	5.46E-05	145(15-159)		LPXTG_anchor: LPXTG cell wall anchor domain(TIGR01167,0.0017,183-211)<br>transmembrane_regions(tmhmm ,-1.0,188-206)
174	129,571	132,447	2877	958	hypothetical protein SXBG_00214	Synechococcus phage S-CAM1	YP_007673127	45.80%	1.88E-83	384(500-883)		
					hypothetical protein Dred_1404	Desulfotomaculum reducens MI-1	YP_001112759	31.60%	1.59E-03	94(584-677)		
175	132,466	133,209	744	247								
176	133,227	133,562	336	111	hypothetical protein MPVG_00058	Micromonas pusilla virus 12T	YP_007676126	39.20%	1.60E-10	102(8-109)		signal-peptide(SignalP-NN(euk),-1.0,1-29)<br>transmembrane_regions(tmhmm ,-1.0,9-27)

ID	Position		Length		BlastP hit in GenBank nr and/or viruses data set						NCBI conserved domain (ID, E-value, alignment length in aa, alignment start - end position)	Interproscan matches (ID, E-value, alignment start - end position)
	start	end	nt	aa	ORF, protein encoded, or mass	Species	Accession	Aa identity	E Value	Alignment length in aa (Start-end position)		
177	133,527	134,729	1203	400	hypothetical protein MpV1_067c	Micromonas sp. RCC1109 virus MpV1	YP_004061950	50.00%	1.55E-99	349(36-384)		signal-peptide(SignalP-NN(euk),-1.0,1-27)
					hypothetical protein	Verrucomicrobia bacterium SCGC AAA164-N20	WP_020036370	46.00%	4.14E-89	365(36-400)		
178	134,743	135,045	303	100								signal-peptide(SignalP-NN(euk),-1.0,1-95)
												transmembrane_regions(tmhmm ,-1.0,52-72;78-98)
179	135,124	135,309	186	61	hypothetical protein BpV1_053c	Bathycoccus sp. RCC1105 virus BpV1	YP_004061483	36.10%	7.55E-03	59(2-60)		signal-peptide(SignalP-NN(euk),-1.0,40-60)
180	135,296	135,721	426	141								
181	135,713	138,064	2352	783	VV A18-like intein-containing helicase	Phaeocystis globosa virus	YP_008052508	40.70%	0	754(10-763)	HELICc Superfamily(cl17351,2.15e-05,100,626-725)	DEAD-like helicases superfamily(SM00487,0.0015,402-592)
					hypothetical protein	Verrucomicrobia bacterium SCGC AAA164-N20	WP_020036356	58.60%	9.33E-133	333(450-782)	Hom_end_hint(pfam05203,2.97e-65,334,116-449)	Homing endonucleases(SSF55608,2.8E-16,302-415)
182	138,105	138,380	276	91								transmembrane_regions(tmhmm ,-1.0,68-88)

ID	Position		Length		BlastP hit in GenBank nr and/or viruses data set						NCBI conserved domain (ID, E-value, alignment length in aa, alignment start - end position)	Interproscan matches (ID, E-value, alignment start - end position)
	start	end	nt	aa	ORF, protein encoded, or mass	Species	Accession	Aa identity	E Value	Alignment length in aa (Start-end position)		
183	138,393	139,139	747	248								transmembrane_regions(tmhmm ,-1.0,224-244)
184	139,149	140,597	1449	482							PAN_APPLE Superfamily(cl00112,5.78e-06,46,157-202)	PAN_4(PF14295,9.4E-8,158-202)
185	140,584	140,835	252	83	hypothetical protein MPWG_00255	Micromonas pusilla virus PL1	AET43739	32.70%	5.75E-07	76(4-79)		
					hypothetical protein	Verrucomicrobia bacterium SCGC AAA164-N20	WP_020036358	27.30%	5.26E-05	78(4-81)		
186	140,832	141,383	552	183	aspartyl/asparaginyl beta-hydroxylase	Burkholderia dolosa	WP_006765711	32.60%	3.99E-13	177(2-178)	Asp_Arg_Hydrox Superfamily(cl17820,2.48e-13,173,9-181)	ASPARTYL/ASPARAGINYL BETA-HYDROXYLASE(PTHR12366,2.2E-15,5-179)
					hypothetical protein OLOG_00074	Ostreococcus lucimarinus virus OlV4	AET84537	30.40%	9.59E-13	177(5-181)		
187	141,434	142,663	1230	409	hypothetical protein OLOG_00133	Ostreococcus lucimarinus virus OlV4	AET84594	24.00%	1.59E-03	190(213-402)		transmembrane_regions(tmhmm ,-1.0,380-400)
188	142,660	143,511	852	283	hypothetical protein OlV1_059	Ostreococcus lucimarinus virus OlV1	YP_004061692	24.30%	4.26E-15	264(1-264)		transmembrane_regions(tmhmm ,-1.0,229-251)
189	143,531	143,755	225	74								signal-peptide(SignalP-NN(euk),-1.0,1-29)

ID	Position		Length		BlastP hit in GenBank nr and/or viruses data set						NCBI conserved domain (ID, E-value, alignment length in aa, alignment start - end position)	Interproscan matches (ID, E-value, alignment start - end position)
	start	end	nt	aa	ORF, protein encoded, or mass	Species	Accession	Aa identity	E Value	Alignment length in aa (Start-end position)		
												transmembrane_regions(tmhmm ,-1.0,9-27)
190	143,812	144,459	648	215	hypothetical protein MpV1_028	Micromonas sp. RCC1109 virus MpV1	YP_004061911	46.60%	1.46E-68	214(2-215)		
					hypothetical protein	Acinetobacter sp. P8-3-8	WP_010113641	25.40%	4.21E-07	172(39-210)		
191	144,483	145,526	1044	347	glycylpeptide N-tetradecanoyltransferase 2	Phytophthora infestans T30-4	XP_002904064	44.20%	1.46E-99	343(4-346)	NMT(pfam01233,5.32e-59,143,4-146)	N-MYRISTOYL TRANSFERASE(PTHR11377,5.6E-135,4-347)
											NMT_C(pfam02799,3.83e-56,181,166-346)	
192	145,515	146,210	696	231	hypothetical protein BpV1_043	Bathycoccus sp. RCC1105 virus BpV1	YP_004061473	56.80%	6.30E-82	227(1-227)		
					hypothetical protein	Verrucomicrobia bacterium SCGC AAA164-N20	WP_020036365	55.10%	9.38E-81	229(1-229)	Pox_A22(pfam04848,2.92e-06,160,2-161)	Pox_A22(PF04848,3.6E-12,2-158)
193	146,246	146,806	561	186	hypothetical protein BpV2_040c	Bathycoccus sp. RCC1105 virus BpV2	ADQ91207	40.90%	2.30E-32	180(7-186)		signal-peptide(SignalP-NN(euk),-1.0,1-16)
194	146,809	147,570	762	253								
195	147,509	147,985	477	158								signal-peptide(SignalP-NN(euk),-1.0,1-27)

ID	Position		Length		BlastP hit in GenBank nr and/or viruses data set						NCBI conserved domain (ID, E-value, alignment length in aa, alignment start - end position)	Interproscan matches (ID, E-value, alignment start - end position)
	start	end	nt	aa	ORF, protein encoded, or mass	Species	Accession	Aa identity	E Value	Alignment length in aa (Start-end position)		
												transmembrane_regions(tmhmm ,-1.0,5-23)
196	147,986	148,303	318	105	hypothetical protein H665_p049	Ostreococcus tauri virus 1	YP_003212873	44.20%	3.10E-20	103(3-105)		
					hypothetical protein	actinobacterium SCGC AAA015-M09	WP_020020241	40.80%	2.83E-17	102(3-104)		
197	148,302	149,237	936	311	hypothetical protein AURANDRAFT_60621	Aureococcus anophagefferens	EGB13093	31.80%	1.85E-18	216(49-264)		signal-peptide(SignalP-NN(euk),-1.0,1-41)
												transmembrane_regions(tmhmm ,-1.0,27-45)
198	149,249	149,929	681	226	transcription elongation factor SII	Micromonas pusilla virus 12T	YP_007676093	35.80%	2.70E-27	186(41-226)	TFIIS_C(cl02609,2.58e-14,60,166-225)	TRANSCRIPTION ELONGATION FACTOR S-II(PTHR11477,9.0E-14,146-225)
					hypothetical protein	Verrucomicrobia bacterium SCGC AAA164-N20	WP_020036216	32.60%	2.41E-20	185(41-225)		
199	149,876	150,421	546	181	GIY-YIG catalytic domain-containing endonuclease	Paramecium bursaria Chlorella virus CZ-2	AGE52752	43.70%	6.35E-16	141(13-153)	GIY-YIG_SF(cl15257,7.70e-24,91,4-94)	grpIintron_endo: group I intron endonuclease(TIGR01453,1.2E-20,3-122)

ID	Position		Length		BlastP hit in GenBank nr and/or viruses data set						NCBI conserved domain (ID, E-value, alignment length in aa, alignment start - end position)	Interproscan matches (ID, E-value, alignment start - end position)
	start	end	nt	aa	ORF, protein encoded, or mass	Species	Accession	Aa identity	E Value	Alignment length in aa (Start-end position)		
200	150,479	151,054	576	191								
201	151,098	151,487	390	129								
202	151,609	151,881	273	90	hypothetical protein OLOG_00017	Ostreococcus lucimarinus virus OlV4	AET84483	40.00%	1.57E-10	84(5-88)		
					hypothetical protein	Verrucomicrobia bacterium SCGC AAA164-N20	WP_020036217	31.00%	7.73E-06	82(7-88)		
203	151,856	152,326	471	156								
204	152,283	152,996	714	237	hypothetical protein MPXG_00183	Micromonas pusilla virus SP1	AET84981	41.00%	1.27E-12	78(48-125)	RING(cl17238,9.61e-08,47,52-98)	zf-RING_2(PF13639,3.2E-7,52-95)
					hypothetical protein	Kluyveromyces lactis NRRL Y-1140	XP_455630	42.90%	8.02E-06	56(40-95)	zf-C3HC4_2(pfam13923,3.15e-04,48,49-96)	
205	152,980	153,240	261	86								signal-peptide(SignalP-NN(euk),-1.0,1-33)
												transmembrane_regions(tmhmm ,-1.0,19-39;54-74)
206	153,247	153,687	441	146								signal-peptide(SignalP-NN(euk),-1.0,1-18)
												transmembrane_regions(tmhmm ,-1.0,29-49;64-82;112-132)

ID	Position		Length		BlastP hit in GenBank nr and/or viruses data set						NCBI conserved domain (ID, E-value, alignment length in aa, alignment start - end position)	Interproscan matches (ID, E-value, alignment start - end position)
	start	end	nt	aa	ORF, protein encoded, or mass	Species	Accession	Aa identity	E Value	Alignment length in aa (Start-end position)		
207	153,740	155,320	1581	526	hypothetical protein OtV6_055	Ostreococcus tauri virus RT-2011	AFC34963	38.50%	1.63E-92	469(56-524)	Cupin_8(pfam13621,1.15e-05,42,78-119;1.21e-04,50,216-265)	HYPOXIA-INDUCIBLE FACTOR 1 ALPHA INHIBITOR-RELATED(PTHR12461,3.2E-4,84-119)
					hypothetical protein Dole_0615	Desulfococcus oleovorans Hxd3	YP_001528502	35.40%	7.10E-05	79(446-524)		Cupin_8(PF13621,1.1E-6,200-275)
208	155,353	155,628	276	91								
209	155,649	155,819	171	56								
210	155,799	156,551	753	250	hypothetical protein BpV2_005c	Bathycoccus sp. RCC1105 virus BpV2	ADQ91172	40.80%	5.38E-52	238(2-239)	YqaJ(cl09232,2.09e-04,88,109-196)	YqaJ(PF09588,5.5E-8,7-200)
					hypothetical protein	Verrucomicrobia bacterium SCGC AAA164-N20	WP_020036581	40.30%	6.57E-50	238(2-239)		
211	156,588	156,875	288	95	unknown	Picea sitchensis	ABK26381	41.00%	1.00E-05	59(36-94)	CP12 Superfamily(cl14670,2.29e-08,61,35-95)	CP12(PF02672,1.9E-10,31-94)
212	156,872	157,249	378	125								signal-peptide(SignalP-NN(euk),-1.0,1-20)
												transmembrane_regions(tmhmm ,-1.0,4-22;43-57;67-87;102-120)
213	157,268	157,558	291	96	hypothetical protein	Paramecium bursaria Chlorella virus 1	NP_048837	41.50%	9.17E-04	43(1-43)	COG5540(COG5540,4.01e-03,40,5-44)	RING/U-box(SSF57850,6.5E-6,1-61)

ID	Position		Length		BlastP hit in GenBank nr and/or viruses data set						NCBI conserved domain (ID, E-value, alignment length in aa, alignment start - end position)	Interproscan matches (ID, E-value, alignment start - end position)
	start	end	nt	aa	ORF, protein encoded, or mass	Species	Accession	Aa identity	E Value	Alignment length in aa (Start-end position)		
214	157,765	158,181	417	138								
215	158,201	158,440	240	79								
216	158,442	158,711	270	89								
217	158,683	159,639	957	318	Heat shock protein 40	Ectocarpus siliculosus	CBJ30599	37.20%	8.35E-49	304(1-304)	DnaJ(cl02542,2.15e-21,49,2-50)	Chaperone J-domain(SSF46565,4.7E-29,1-103)
											DnaJ_C Superfamily(cl03262,6.20e-22,206,99-304)	
											DnaJ_bact(TIGR02349,5.86e-79,315,2-316)	
218	159,667	160,065	399	132								
219	160,087	161,685	1599	532	sensor histidine kinase	Parabacteroides johnsonii	WP_008147758	25.40%	3.35E-03	207(307-513)		
220	161,722	161,976	255	84								
221	162,033	162,608	576	191								
222	162,627	163,451	825	274	DNA ligase III	Comamonas testosteroni	WP_003062464	33.30%	9.73E-03	109(9-117)		
223	164,062	164,256	195	64								
224	164,307	164,858	552	183	predicted protein	Populus trichocarpa	XP_002305796	36.10%	6.08E-19	145(19-163)	CDC48_N(pfam02359,5.13e-03,75,20-94)	VESICULAR-FUSION PROTEIN NSF(PTHR23078,2.6E-14,9-159)
											CDC48_2 Superfamily(cl08380,6.85e-03,56,119-174)	
225	164,882	165,097	216	71	hypothetical protein PBCVKS1B_593L	Paramecium bursaria Chlorella	AGE54713	52.00%	3.84E-05	48(8-55)		

ID	Position		Length		BlastP hit in GenBank nr and/or viruses data set						NCBI conserved domain (ID, E-value, alignment length in aa, alignment start - end position)	Interproscan matches (ID, E-value, alignment start - end position)
	start	end	nt	aa	ORF, protein encoded, or mass	Species	Accession	Aa identity	E Value	Alignment length in aa (Start-end position)		
						virus KS1B						
					hypothetical protein Dalk_3573	Desulfatibacillum alkenivorans AK-01	YP_002432729	38.90%	3.31E-03	54(3-56)		
226	165,204	165,509	306	101								
227	165,531	166,034	504	167	hypothetical protein	Arthrobacter sp. M2012083	WP_017197732	37.20%	5.04E-21	159(2-160)		
228	166,031	166,651	621	206	phosphate starvation protein PhoH	Verrucomicrobia bacterium SCGC AAA164-N20	WP_020036220	51.30%	8.71E-63	198(8-205)	PhoH(cl17668,2.51e-88,206,1-206)	PhoH(PF02562,5.9E-71,2-206)
					hypothetical protein MpV1_231c	Micromonas sp. RCC1109 virus MpV1	YP_004062114	52.80%	2.72E-69	197(9-205)		
229	166,634	167,470	837	278								
230	167,469	167,630	162	53								
231	167,696	168,028	333	110								
232	168,031	168,258	228	75								
233	168,277	169,191	915	304							PLN03223(PLN03223,3.22e-03,102,108-209)	
234	169,212	169,895	684	227								
235	169,916	170,608	693	230	hypothetical protein	Arthrobacter sp. 131MFCol6.1	WP_018775835	40.60%	4.96E-48	227(4-230)	Glyco_hydro_cc Superfamily(cl13267,3.17e-51,165,63-227)	Glyco_hydro_cc(PF11790,1.0E-53,10-227)
236	170,654	171,067	414	137	PREDICTED: RING finger protein 145-like	Metaseiulus occidentalis	XP_003744587	43.20%	2.40E-09	73(3-75)	RING(cd00162,8.13e-06,44,2-45)	RING/U-box(SSF57850,5.3E-14,2-49)
											zf-C3HC4_2(pfam13923,7.12e-05,43,2-44)	

ID	Position		Length		BlastP hit in GenBank nr and/or viruses data set						NCBI conserved domain (ID, E-value, alignment length in aa, alignment start - end position)	Interproscan matches (ID, E-value, alignment start - end position)
	start	end	nt	aa	ORF, protein encoded, or mass	Species	Accession	Aa identity	E Value	Alignment length in aa (Start-end position)		
YSLGV												
1	47	226	180	59								signal-peptide(SignalP-NN(euk),-1.0,1-44) transmembrane_regions(tmhmm,-1.0,29-49)
2	341	667	327	108								
3	679	831	153	50								
4	1,100	1,336	237	78	hypothetical protein	Acanthamoeba polyphaga mimivirus	YP_003986937	46.50%	4.43E-03	43(15-57)		
5	1,769	2,698	930	309	hypothetical protein	Ruminococcus sp. JC304	WP_019163853	35.60%	7.59E-38	247(34-280)		
					hypothetical protein LBA_00945	Megavirus lba	YP_007418839	33.20%	5.31E-36	301(2-302)		
6	2,816	2,968	153	50	hypothetical protein SSM2_108	Synechococcus phage S-SM2	YP_004322264	75.90%	1.86E-03	29(13-41)		
7	2,989	3,738	750	249							zf-C3HC4_2(pfam13923,6.65e-03,44,162-205)	
8	3,761	3,982	222	73								
9	4,041	4,730	690	229								
10	4,800	5,444	645	214	hypothetical protein EMIHUDRAFT_451978	Emiliania huxleyi CCMP1516	EOD13544	39.10%	1.40E-06	66(141-206)		ZF_RING_2(PS50089,10.97,165-204) RING/U-box(SSF57850,9.4E-11,160-214)

ID	Position		Length		BlastP hit in GenBank nr and/or viruses data set						NCBI conserved domain (ID, E-value, alignment length in aa, alignment start - end position)	Interproscan matches (ID, E-value, alignment start - end position)
	start	end	nt	aa	ORF, protein encoded, or mass	Species	Accession	Aa identity	E Value	Alignment length in aa (Start-end position)		
11	5,502	5,870	369	122	hypothetical protein OLV2	Organic Lake virophage	ADX05763	38.50%	1.44E-07	90(33-122)		
					hypothetical protein GUITHDRAFT_82952	Guillardia theta CCMP2712	EKX31624	34.10%	1.72E-02	82(41-122)		
12	5,824	6,198	375	124	hypothetical protein ATCVBr0604L_859R	Acanthocystis turfacea Chlorella virus Br0604L	AGE49303	49.40%	3.15E-14	80(43-122)		
13	6,304	6,813	510	169	hypothetical protein 162319347	Organic Lake phycodnavirus 2	ADX06339	43.80%	7.04E-18	126(3-128)		
					hypothetical protein EMIHUDRAFT_463777	Emiliania huxleyi CCMP1516	EOD21788	24.50%	2.06E-02	140(9-148)		
14	6,810	6,983	174	57								
15	7,002	8,900	1,899	632	hypothetical protein 162319347	Organic Lake phycodnavirus 2	ADX06339	35.20%	2.07E-69	500(5-504)	Cas3_I-D(cd09710,8.16e-03,43,208-250)	P-loop containing nucleoside triphosphate hydrolases(SSF52540,2.5E-6,157-251;4.6E-6,24-237)
16	9,117	9,347	231	76								
17	9,425	10,543	1,119	372	late transcription factor VLTF3	Phaeocystis globosa virus	YP_008052476	57.10%	7.81E-122	368(5-372)	Pox_VLTF3(pfam04947,7.26e-38,164,206-369)	Pox_VLTF3(PF04947,3.0E-46,206-370)
18	10,561	10,848	288	95	hypothetical protein 162319399	Organic Lake phycodnavirus 2	ADX06361	68.80%	2.20E-23	77(17-93)		signal-peptide(SignalP-NN(euk),-1.0,1-16)

ID	Position		Length		BlastP hit in GenBank nr and/or viruses data set						NCBI conserved domain (ID, E-value, alignment length in aa, alignment start - end position)	Interproscan matches (ID, E-value, alignment start - end position)
	start	end	nt	aa	ORF, protein encoded, or mass	Species	Accession	Aa identity	E Value	Alignment length in aa (Start-end position)		
					hypothetical protein	Ruminococcus sp. JC304	WP_019163465	49.30%	4.01E-09	72(22-93)		transmembrane_regions(tmhmm,-1.0,27-45;51-71)
19	10,910	11,227	318	105	hypothetical protein PGCG_00067	Phaeocystis globosa virus	YP_008052386	58.40%	1.25E-22	77(29-105)		
20	11,421	11,846	426	141	hypothetical protein PGCG_00067	Phaeocystis globosa virus	YP_008052386	51.10%	1.91E-33	131(1-131)		
21	12,044	12,499	456	151							Peptidase_M13_N(pfam05649,8.60 e-06,123,12-134)	
22	12,683	13,177	495	164	PREDICTED: putative zinc metalloproteinase T16A9.4-like isoform 1	Nasonia vitripennis	XP_001599960	27.00%	2.77E-03	125(39-163)	Peptidase_M13_N super family(cl14911,2.91e-14,161,3-163)	Peptidase_M13_N(PF05649,9.9E-13,11-163)
23	13,281	13,628	348	115	endopeptidase	Lactobacillus casei subsp. casei ATCC 393	BAN74100	48.00%	2.59E-14	75(35-109)	M13(cd08662,4.27e-21,75,36-110)	Peptidase_M13(PF01431,1.0E-16,38-113)
24	13,683	14,000	318	105	putative metalloendopeptidase	Prevotella sp. CAG:485	WP_022405175	35.20%	1.07E-05	100(5-104)	Peptidase_M13 super family(cl17766,8.20e-10,93,5-97)	Peptidase_M13(PF01431,2.0E-13,4-101)
25	14,400	15,239	840	279	VV A32-like packaging ATPase	Phaeocystis globosa virus	YP_008052384	78.10%	3.20E-161	279(1-279)		Pox_A32(PF04665,9.2E-7,94-225)
					hypothetical protein	Verrucomicrobia bacterium SCGC AAA164-N20	WP_020036342	46.40%	3.14E-75	247(1-247)		

ID	Position		Length		BlastP hit in GenBank nr and/or viruses data set						NCBI conserved domain (ID, E-value, alignment length in aa, alignment start - end position)	Interproscan matches (ID, E-value, alignment start - end position)
	start	end	nt	aa	ORF, protein encoded, or mass	Species	Accession	Aa identity	E Value	Alignment length in aa (Start-end position)		
26	15,682	16,863	1,182	393	phosphotransferase GIV83-like protein	Phaeocystis globosa virus	YP_008052382	35.10%	2.85E-42	384(1-384)		
					hypothetical protein	Bacillus subtilis	WP_003239010	26.80%	5.68E-02	184(81-264)		
27	16,772	17,071	300	99	phosphotransferase GIV83-like protein	Organic Lake phycodnavirus 2	ADX06274	54.40%	3.36E-23	90(6-95)		
28	17,043	17,195	153	50								
29	17,423	17,926	504	167	hypothetical protein 162300046	Organic Lake phycodnavirus 2	ADX06204	75.00%	1.25E-67	160(8-167)		
					putative uncharacterized protein	Bacteroides pectinophilus CAG:437	WP_022361645	34.40%	8.25E-07	128(21-148)		
30	18,035	19,054	1,020	339	putative site-specific DNA methyltransferase	Organic Lake phycodnavirus 2	ADX06203	64.00%	2.83E-159	339(1-339)	MethyltransfD12(cl17344,1.05e-51,296,43-338)	dam: DNA adenine methylase(TIGR00571,2.0E-63,43-338)
					hypothetical protein	Verrucomicrobia bacterium SCGC AAA164-N20	WP_020036269	45.50%	3.08E-82	297(43-339)		
31	19,180	19,401	222	73	hypothetical protein PGCG_00059	Phaeocystis globosa virus	YP_008052378	45.80%	1.23E-06	69(5-73)		signal-peptide(SignalP-NN(euk),-1.0,1-20)
												transmembrane_regions(tmhmm,-1.0,37-55)
32	19,440	20,198	759	252								
33	20,174	20,575	402	133	hypothetical protein H072_7704	Dactylellina haptotyla CBS 200.50	EPS38540	50.00%	8.37E-03	32(99-130)	PRK12686(PRK12686,8.78e-03,54,43-96)	
34	20,572	21,567	996	331	hypothetical protein PGCG_00058	Phaeocystis globosa virus	YP_008052377	62.00%	9.24E-67	199(1-199)		

ID	Position		Length		BlastP hit in GenBank nr and/or viruses data set						NCBI conserved domain (ID, E-value, alignment length in aa, alignment start - end position)	Interproscan matches (ID, E-value, alignment start - end position)
	start	end	nt	aa	ORF, protein encoded, or mass	Species	Accession	Aa identity	E Value	Alignment length in aa (Start-end position)		
35	21,642	22,289	648	215	replication factor C small subunit	Phaeocystis globosa virus	YP_008052376	41.20%	1.00E-37	215(1-215)	rfc(PRK00440,1.61e-16,80,9-88)	P-loop containing nucleoside triphosphate hydrolases(SSF52540,1.8E-12,4-107)
					DNA replication ATPase	Thermococcus onnurineus NA1	YP_002307801	41.10%	5.08E-13	107(3-109)		REPLICATION FACTOR C / DNA POLYMERASE III GAMMA-TAU SUBUNIT(PTHR11669,5.1E-14,10-83)
36	22,289	22,531	243	80	unknown	Picea sitchensis	ADE77654	37.70%	5.54E-05	53(26-78)	AAA(cl17189,1.13e-03,25,46-70)	no description(3.40.50.300,2.6E-6,29-77)
37	22,622	22,813	192	63								
38	22,830	24,602	1,773	590	hypothetical protein PGCG_00100	Phaeocystis globosa virus	YP_008052419	35.40%	7.31E-87	588(3-590)		signal-peptide(SignalP-NN(euk),-1.0,1-17)
39	24,617	25,093	477	158	hypothetical protein 162300206	Organic Lake phycodnavirus 2	ADX06279	43.80%	3.09E-30	153(1-153)		
40	25,143	27,155	2,013	670	glycosyl transferase	Actinobacillus minor	WP_005824596	40.60%	6.60E-63	359(3-361)	WcaA(COG0463,1.45e-06,298,3-300)	Glycos_transf_2(PF00535,1.7E-9,10-111)

ID	Position		Length		BlastP hit in GenBank nr and/or viruses data set						NCBI conserved domain (ID, E-value, alignment length in aa, alignment start - end position)	Interproscan matches (ID, E-value, alignment start - end position)
	start	end	nt	aa	ORF, protein encoded, or mass	Species	Accession	Aa identity	E Value	Alignment length in aa (Start-end position)		
											Glyco_tranf_GTA_type(cl11394,9.71e-18,150,5-154)	TETRATRICOPEPTIDE REPEAT PROTEIN, TPR(PTHR23083,1.6E-5,187-517)
											TPR(cl02429,1.76e-05,92,198-289)	Nucleotide-diphospho-sugar transferases(SSF53448,8.3E-17,1-97)
41	27,161	27,523	363	120	hypothetical protein PGCG_00186	Phaeocystis globosa virus	YP_008052504	37.60%	5.25E-13	116(1-116)		
42	27,535	27,879	345	114								
43	27,917	28,126	210	69								
44	28,099	28,593	495	164	hypothetical protein ACD_9C00040G0003	uncultured bacterium	EKE19361	41.60%	2.89E-23	142(2-143)		
45	28,652	29,221	570	189	hypothetical protein PGCG_00184	Phaeocystis globosa virus	YP_008052502	39.60%	4.35E-32	182(5-186)		
46	29,404	29,700	297	98								
47	30,054	30,881	828	275	putative DNA N6-adenine methyltransferase	Cafeteria roenbergensis virus BV-PW1	YP_003969706	60.70%	5.99E-90	272(2-273)	AdoMet_MTases Superfamily(cl17173,1.38e-06,91,38-128)	S-adenosyl-L-methionine-dependent methyltransferases(SSF53335,1.0E-9,6-179)
					hypothetical protein	Sphingomonas echinoides	WP_010402503	31.90%	1.69E-28	258(12-269)		

ID	Position		Length		BlastP hit in GenBank nr and/or viruses data set						NCBI conserved domain (ID, E-value, alignment length in aa, alignment start - end position)	Interproscan matches (ID, E-value, alignment start - end position)
	start	end	nt	aa	ORF, protein encoded, or mass	Species	Accession	Aa identity	E Value	Alignment length in aa (Start-end position)		
48	30,963	31,688	726	241	hypothetical protein PGCG_00183	Phaeocystis globosa virus	YP_008052501	33.50%	3.92E-28	212(5-216)		transmembrane_regions(tmhmm,-1.0,37-71)
49	31,708	32,832	1,125	374	hypothetical protein PGCG_00181	Phaeocystis globosa virus	YP_008052499	38.70%	5.95E-49	285(9-293)		
					GJ23634	Drosophila virilis	XP_002052993	35.30%	1.71E-02	68(279-346)		
50	32,878	33,258	381	126	hypothetical protein	Verrucomicrobia bacterium SCGC AAA164-N20	WP_020036351	27.00%	3.28E-03	87(1-87)		
					hypothetical protein BpV1_089c	Bathycoccus sp. RCC1105 virus BpV1	YP_004061519	27.60%	3.91E-03	87(1-87)		
51	33,328	33,555	228	75								signal-peptide(SignalP-NN(euk),-1.0,1-22)
												transmembrane_regions(tmhmm,-1.0,5-23;33-53)
52	33,564	34,148	585	194	hypothetical protein PGCG_00179	Phaeocystis globosa virus	YP_008052497	46.90%	1.68E-44	162(1-162)		
53	34,250	35,266	1,017	338	DNA-directed RNA polymerase II subunit D	Phaeocystis globosa virus	YP_008052496	42.00%	2.97E-79	316(3-318)	RNAP_RPB11_RPB3(cl11409,3.89e-25,261,3-263)	RBP11-like subunits of RNA polymerase(SSF55257,1.4E-18,2-273)

ID	Position		Length		BlastP hit in GenBank nr and/or viruses data set						NCBI conserved domain (ID, E-value, alignment length in aa, alignment start - end position)	Interproscan matches (ID, E-value, alignment start - end position)
	start	end	nt	aa	ORF, protein encoded, or mass	Species	Accession	Aa identity	E Value	Alignment length in aa (Start-end position)		
					DNA directed RNA polymerase subunit D	uncultured organism MedDCM-OCT-S08-C195	ADD95766	41.20%	4.13E-76	315(1-315)	PRK00783(PRK00783,4.49e-22,263,3-265)	DNA-DIRECTED RNA POLYMERASE(PTHR11800,9.7E-9,2-103)
54	35,212	35,424	213	70	DNA directed RNA polymerase subunit D	uncultured organism MedDCM-OCT-S08-C195	ADD95766	38.60%	3.65E-07	57(14-70)		
					DNA-directed RNA polymerase II subunit D	Phaeocystis globosa virus	YP_008052496	33.30%	3.57E-02	57(14-70)		
55	35,637	36,620	984	327	hypothetical protein PGCG_00176	Phaeocystis globosa virus	YP_008052494	40.40%	1.89E-56	306(18-323)	RNA_lig_T4_1(cl09743,1.29e-05,122,17-138)	
					predicted protein	Naegleria gruberi	XP_002671015	23.80%	2.53E-03	156(17-172)		
56	36,829	37,071	243	80								
57	37,415	37,582	168	55								
58	37,572	38,105	534	177	hypothetical protein PGCG_00175	Phaeocystis globosa virus	YP_008052493	46.40%	2.11E-10	81(5-85)		
					lipoprotein	Myxococcus xanthus DK 1622	YP_630411	65.60%	2.51E-04	32(96-127)		
59	38,158	39,372	1,215	404	hypothetical protein PGCG_00175	Phaeocystis globosa virus	YP_008052493	25.60%	3.04E-22	384(14-397)		
60	39,375	41,225	1,851	616	hypothetical protein PGCG_00175	Phaeocystis globosa virus	YP_008052493	31.10%	4.27E-73	603(7-609)		
61	41,225	43,267	2,043	680	hypothetical protein PGCG_00175	Phaeocystis globosa virus	YP_008052493	30.50%	2.88E-59	665(3-667)		

ID	Position		Length		BlastP hit in GenBank nr and/or viruses data set						NCBI conserved domain (ID, E-value, alignment length in aa, alignment start - end position)	Interproscan matches (ID, E-value, alignment start - end position)
	start	end	nt	aa	ORF, protein encoded, or mass	Species	Accession	Aa identity	E Value	Alignment length in aa (Start-end position)		
62	43,360	43,608	249	82	hypothetical protein	Marine Group II euryarchaeote SCGC AB-629-J06	WP_018036027	51.90%	1.31E-14	79(4-82)		signal-peptide(SignalP-NN(euk),-1.0,1-31)
					hypothetical protein PGCG_00174	Phaeocystis globosa virus	YP_008052492	46.80%	3.72E-13	77(6-82)		transmembrane_regions(tmhmm,-1.0,10-28;53-75)
63	43,713	44,654	942	313	hypothetical protein PGCG_00173	Phaeocystis globosa virus	YP_008052491	21.60%	4.26E-05	287(6-292)		signal-peptide(SignalP-NN(euk),-1.0,1-21)
												transmembrane_regions(tmhmm,-1.0,5-20 ;39-59;74-96;252-272)
64	44,809	45,060	252	83	PREDICTED: VPS10 domain-containing receptor SorCS2	Chrysemys picta bellii	XP_005300817	35.00%	6.83E-02	58(15-72)		signal-peptide(SignalP-NN(euk),-1.0,1-29)
					hypothetical protein PGCG_00172	Phaeocystis globosa virus	YP_008052490	35.40%	5.80E-05	78(3-80)		transmembrane_regions(tmhmm,-1.0,10-30)
65	45,093	46,172	1,080	359	hypothetical protein PGCG_00171	Phaeocystis globosa virus	YP_008052489	25.40%	2.31E-05	217(27-243)		
66	46,423	46,656	234	77	hypothetical protein 162300232	Organic Lake phycodnavirus 2	ADX06292	40.80%	6.10E-13	76(1-76)		signal-peptide(SignalP-NN(euk),-1.0,1-26)

ID	Position		Length		BlastP hit in GenBank nr and/or viruses data set						NCBI conserved domain (ID, E-value, alignment length in aa, alignment start - end position)	Interproscan matches (ID, E-value, alignment start - end position)
	start	end	nt	aa	ORF, protein encoded, or mass	Species	Accession	Aa identity	E Value	Alignment length in aa (Start-end position)		
67	46,747	46,995	249	82	hypothetical protein PGCG_00169	Phaeocystis globosa virus	YP_008052487	40.00%	3.20E-11	77(6-82)		transmembrane_regions(tmhmm,-1.0,5-25)<br>signal-peptide(SignalP-NN(euk),-1.0,1-28)<br>transmembrane_regions(tmhmm,-1.0,15-35)
68	47,027	47,431	405	134	hypothetical protein PGCG_00168	Phaeocystis globosa virus	YP_008052486	42.10%	1.53E-08	90(41-130)		
69	47,675	48,169	495	164								
70	48,233	48,409	177	58								transmembrane_regions(tmhmm,-1.0,26-46)
71	48,630	48,806	177	58							Patatin_and_cPLA2(cl11396,2.04e-03,42,1-42)	
72	48,836	49,108	273	90								transmembrane_regions(tmhmm,-1.0,46-64)
73	49,201	49,533	333	110	Similar to Uncharacterized RING finger protein P4H10.07; acc. no. Q9P7E1	Pyronema omphalodes CBS 100304	CCX29987	34.50%	2.22E-02	55(8-62)		transmembrane_regions(tmhmm,-1.0,87-107)
74	49,819	50,169	351	116	polyA polymerase catalytic subunit	Phaeocystis globosa virus	YP_008052392	51.40%	4.57E-30	107(4-110)		

ID	Position		Length		BlastP hit in GenBank nr and/or viruses data set						NCBI conserved domain (ID, E-value, alignment length in aa, alignment start - end position)	Interproscan matches (ID, E-value, alignment start - end position)
	start	end	nt	aa	ORF, protein encoded, or mass	Species	Accession	Aa identity	E Value	Alignment length in aa (Start-end position)		
75	50,319	51,215	897	298	polyA polymerase catalytic subunit	Phaeocystis globosa virus	YP_008052392	54.70%	2.46E-87	247(52-298)		
76	51,118	52,224	1,107	368	PREDICTED: inactive phospholipase C-like protein 2-like	Xenopus (Silurana) tropicalis	XP_002942363	27.20%	6.63E-03	102(64-165)	PI-PLC-X(cl17177,2.99e-03,119,64-182)	PLC-like phosphodiesterases(SSF51695,4.3E-12,61-352)
					hypothetical protein PGCG_00074	Phaeocystis globosa virus	YP_008052393	29.70%	1.87E-33	347(8-354)	PI-PLCc_GDPD_SF(cl14615,4.87e-03,57,266-322)	
77	52,275	53,966	1,692	563	major capsid protein MCP2	Phaeocystis globosa virus	YP_008052394	47.60%	4.95E-158	562(1-562)	Capsid_NCLDV(pfam04451,4.17e-12,271,289-559)	Capsid_NCLDV(PF04451,3.6E-32,289-543)
												Group II dsDNA viruses VP(SSF49749,2.0E-33,25-309;4.3E-12,419-563)
												African swine fever virus, p72, major capsid(2.70.9.20,2.6E-18,423-562)
78	54,083	54,361	279	92								
79	54,301	54,780	480	159	hypothetical protein	Arthrospira platensis	WP_006624940	34.40%	3.30E-02	64(48-111)		
					hypothetical protein PSSM2_192	Prochlorococcus phage P-SSM2	YP_214424	30.80%	5.54E-04	65(47-111)		

ID	Position		Length		BlastP hit in GenBank nr and/or viruses data set						NCBI conserved domain (ID, E-value, alignment length in aa, alignment start - end position)	Interproscan matches (ID, E-value, alignment start - end position)
	start	end	nt	aa	ORF, protein encoded, or mass	Species	Accession	Aa identity	E Value	Alignment length in aa (Start-end position)		
80	54,883	55,683	801	266	D12 class N6 adenine-specific DNA methyltransferase	Cyanothece sp. PCC 7425	WP_012629081	38.10%	7.78E-55	257(6-262)	MethyltransfD12(cl17344,1.00e-23,242,3-244)	MethyltransfD12(PF02086,1.2E-25,10-247)
												S-adenosyl-L-methionine-dependent methyltransferases(SSF53335,5.8E-36,1-262)
81	55,794	56,366	573	190	hypothetical protein	Desulfotomaculum acetoxidans	WP_015759445	27.70%	1.27E-02	151(1-151)		signal-peptide(SignalP-NN(euk),-1.0,1-18)
82	56,398	56,958	561	186								signal-peptide(SignalP-NN(euk),-1.0,1-50)
												transmembrane_regions(tmhmm ,-1.0,27-47;159-179)
83	57,005	57,418	414	137								signal-peptide(SignalP-NN(euk),-1.0,1-16)
												transmembrane_regions(tmhmm ,-1.0,5-25;71-91;112-132)
84	57,615	58,127	513	170	hypothetical protein	Prochlorothrix hollandica	WP_017711778	43.40%	3.04E-03	74(47-120)		

ID	Position		Length		BlastP hit in GenBank nr and/or viruses data set						NCBI conserved domain (ID, E-value, alignment length in aa, alignment start - end position)	Interproscan matches (ID, E-value, alignment start - end position)
	start	end	nt	aa	ORF, protein encoded, or mass	Species	Accession	Aa identity	E Value	Alignment length in aa (Start-end position)		
85	58,152	58,325	174	57								
86	58,362	59,147	786	261	glycosyl transferase	Phaeocystis globosa virus 14T	AET73754	49.30%	7.45E-71	257(1-257)	Glyco_tranf_GTA_type(cl11394,1.64e-06,59,3-61)	Glycos_transf_2(PF00535,1.7E-5,4-52)
					hypothetical protein	Uliginosibacterium gangwonense	WP_018610783	45.70%	5.72E-37	164(3-166)		Nucleotide-diphospho-sugar transferases(SSF53448,9.5E-10,3-91)
87	59,453	59,701	249	82								transmembrane_regions(tmhmm,-1.0,50-70)
88	59,600	60,913	1,314	437	ZYRO0F06446p	Zygosaccharomyces rouxii	XP_002497478	27.50%	7.99E-17	223(5-227)	AAA(cl17189,2.20e-11,135,33-167)	ATPases associated with a variety of cellula(SM00382,6.1E-8,35-173)
					replication factor C large subunit	Phaeocystis globosa virus	YP_008052362	43.90%	6.41E-83	431(1-431)		AAA(PF00004,1.6E-11,39-155)
												CHROMOSOME TRANSMISSION FIDELITY FACTOR 18(PTHR23389,4.6E-22,26-177)
89	60,938	61,615	678	225								

ID	Position		Length		BlastP hit in GenBank nr and/or viruses data set						NCBI conserved domain (ID, E-value, alignment length in aa, alignment start - end position)	Interproscan matches (ID, E-value, alignment start - end position)
	start	end	nt	aa	ORF, protein encoded, or mass	Species	Accession	Aa identity	E Value	Alignment length in aa (Start-end position)		
90	61,596	61,949	354	117	hypothetical protein 162300258	Organic Lake phycodnavirus 2	ADX06305	35.60%	6.90E-09	102(7-108)		signal-peptide(SignalP-NN(euk),-1.0,1-32)
												transmembrane_regions(tmhmm ,-1.0,9-29)
91	61,985	62,473	489	162	hypothetical protein PGCG_00050	Phaeocystis globosa virus	YP_008052369	44.70%	3.47E-37	159(4-162)		
92	62,489	63,508	1,020	339	alanine racemase	Kribbella flavida DSM 17836	YP_003383843	35.80%	6.89E-50	338(1-338)	Alr(COG0787,6.01e-88,338,1-338)	alr: alanine racemase(TIGR00492,3.7E-79,1-337)
93	63,853	65,310	1,458	485	DNA gyrase/DNA topoisomerase IV subunit A	Phaeocystis globosa virus	YP_008052370	51.10%	8.06E-138	468(2-469)	TOP4c(smart00434,2.92e-115,435,1-435)	DNA TOPOISOMERASE/GYRASE(PTHR10169,1.2E-169,1-478)
					hypothetical protein GUITHDRAFT_158481	Guillardia theta CCMP2712	EKX38687	40.50%	2.79E-100	440(1-440)		DNA_topoisoIV(PF00521,4.2E-102,1-440)
94	65,433	67,364	1,932	643	DNA gyrase/DNA topoisomerase IV subunit A	Phaeocystis globosa virus	YP_008052370	63.30%	0	633(2-634)	TOP2c(smart00433,1.45e-153,553,57-609)	DNA TOPOISOMERASE/GYRASE(PTHR10169,4.8E-248,4-634)
					DNA topoisomerase II	Galdieria sulphuraria	XP_005709173	46.10%	2.19E-171	625(10-634)		TopoisomeraseII(SM00433,1.7E-142,57-637)
95	67,420	67,701	282	93								
96	67,738	68,175	438	145	hypothetical protein PGCG_00095	Phaeocystis globosa virus	YP_008052414	57.90%	2.88E-18	95(1-95)		

ID	Position		Length		BlastP hit in GenBank nr and/or viruses data set						NCBI conserved domain (ID, E-value, alignment length in aa, alignment start - end position)	Interproscan matches (ID, E-value, alignment start - end position)
	start	end	nt	aa	ORF, protein encoded, or mass	Species	Accession	Aa identity	E Value	Alignment length in aa (Start-end position)		
97	68,309	69,949	1,641	546	EsV-1-52	Ectocarpus siliculosus	CBN80356	67.40%	3.49E-05	43(23-65)		
					hypothetical protein 162322300	Organic Lake phycodnavirus 1	ADX05824	30.00%	1.49E-34	437(89-525)		
98	69,970	70,599	630	209								signal-peptide(SignalP-NN(euk),-1.0,1-27)
												transmembrane_regions(tmhmm ,-1.0,9-27)
99	70,714	71,220	507	168								
100	71,385	71,831	447	148								
101	71,912	72,745	834	277	putative proliferating cell nuclear antigen	Phaeocystis globosa virus	YP_008052411	74.00%	4.59E-133	269(9-277)	PCNA(cl09515,2.58e-31,250,19-268)	pcna: proliferating cell nuclear antigen (pcna)(TIGR00590,3.8E-40,20-269)
					hypothetical protein	Verrucomicrobia bacterium SCGC AAA164-N20	WP_020036354	32.60%	8.89E-37	257(17-273)		
102	72,830	73,291	462	153	hypothetical protein, partial	Bacillus cereus	WP_000464019	70.60%	1.11E-14	68(25-92)		

Table S3 Putative emrE in YSLPV1-2 predicted by using HHpred

Protein Name	Best Hit	Description	E-value	Probabilities
YSLPV1-ORF29	3b5d_A	Multidrug transporter EMRE	4.2E-29	100
YSLPV1-ORF218	3b5d_A	Multidrug transporter EMRE	1.4E-04	97.4
YSLPV2-ORF158	3b5d_A	Multidrug transporter EMRE	8.6E-27	99.9
YSLPV2-ORF210	3b5d_A	Multidrug transporter EMRE	2.1E-29	100

Table S4 Homologs shared between virophages and giant viruses

Query (Virophage)		Subject (Giant Virus)		E value	identity
Name	Gene product	Name	Gene product		
Sputnik	V3 FtsK–HerA superfamily ATPase	Acanthamoeba polyphaga mimivirus	MIMI L712	0.01	54%
	V6 Collagen triple-helixrepeat-containing protein		MIMI R196	4.00E-19	53%
	V7 Collagen triple-helixrepeat-containing protein		MIMI R239	0.001	27%
	V12		MIMI R546	5.00E-42	64%
	V13 DNA-polymerase		MIMI L207/206	4.00E-12	29%
Organic lake virophage	OLV17 putative transmembrane protein	Organic Lake phycodnavirus	OLPV Lipoprotein	1.00E-24	40%
	OLV18 hp		OLPV Cyanothece sp. cce_0037-like protein	2.00E-33	65%
	OLV19 hp		OLPV Lipoprotein	9.00E-33	65%
	OLV20 Collagen triple-helixrepeat-containing protein		OLPV hp	1.00E-07	32%
	OLV22		OLPV hp	5.00E-31	56%
Mavirus	putative superfamily 3 helicase	Cafeteria roenbergensis virus	putative RecQ-like ATP-dependent DNA helicase	2.00E-04	25%
	hypothetical protein		putative ubiquitin carboxyl-terminal hydrolase	2.00E-04	32%
	putative cysteine protease		putative cysteine protease	7.00E-06	25%
Ace lake mavirus	ORF02 D5-like helicase-primase		Crov319 hp	1.00E-04	26%
	ORF13 hp		CroV276 hp	3.00E-08	39%
YSLV1	ORF28	Yellowstone Lake phycodnavirus, -mimivirus	YSLGV-ORF11	2.33E-05	38%
			YSLPV1-ORF55	2.45E-09	40%
			YSLPV2-ORF123	1.31E-05	30.70%

Query (Virophage)		Subject (Giant Virus)		E value	identity
Name	Gene product	Name	Gene product		
			YSLPV3-ORF59	2.33E-08	39.70%
	ORF07		YSLPV1-ORF226	2.67E-13	24.70%
			YSLPV3-ORF89	8.30E-16	29.20%
	ORF15		YSLGV-ORF11	> E-02	30.80%
	ORF22		YSLPV2-ORF04	> E-02	24.30%
YSLV2	ORF05	Yellowstone Lake phycodnavirus, -giant virus	YSLPV1-ORF99	> E-02	29.40%
	ORF06 Methyltransferase domain?		OLPV2 hp	6.07E-07	50.00%
	ORF20 OLV2 homolog		YSLGV-ORF11	> E-02	33.30%
			YSLPV1-ORF55	2.69E-10	38.80%
			YSLPV2-ORF123	9.19E-09	40%
			YSLPV3-ORF59	4.17E-15	38.80%
YSLV3	ORF03 OLV2 homolog	Yellowstone Lake phycodnavirus, -giant virus	YSLGV-ORF11	1.76E-05	35.50%
	ORF07 methylase		YSLGV-ORF30 site-specific methyltrasferase	1.22E-04	35.70%
			YSLPV1-ORF236 site-specific methyltrasferase	7.68E-10	25.30%
	ORF16		YSLPV1-ORF86 Serine/threonine-protein kinase	> E-02	31.50%
YSLV4	ORF26 OLV2 homolog	Yellowstone Lake phycodnavirus, -giant virus	YSLGV-ORF11	8.75E-24	53.30%
			YSLPV1-ORF55	5.34E-04	39.10%
			YSLPV2-ORF123	5.35E-05	32.90%
			YSLPV3-ORF59	4.82E-06	35.20%
	ORF30		YSLPV3-ORF236	> E-02	31.10%

Query (Virophage)		Subject (Giant Virus)		E value	identity
Name	Gene product	Name	Gene product		
	ORF32 N-Acetyltransferase		YSLPV2-ORF184 GNAT family acetyltransferase ?	1.08E-04	27.10%
			YSLPV3-ORF39 GNAT family acetyltransferase ?	1.09E-05	29.90%
	ORF04 RNR2		YSLPV1-ORF82 RNR2	1.07E-91	50.50%
			YSLPV2-ORF94 RNR2	1.07E-95	51.30%
			YSLPV3-ORF92 RNR2	2.83E-95	50.80%
YSLV6	ORF18 OLV2 homolog	Yellowstone lake phycodnavirus, -giant virus	YSLGV-ORF11	1.56E-11	37.80%
			YSLPV1-ORF55	2.01E-04	37.30%
			YSLPV2-ORF123	1.50E-06	37.80%
			YSLPV3-ORF59	1.39E-05	37.10%

Table S5 Homologs shared between PgVV and virophages

PgVV gene product	*aa length	Blast hits(aa length)	E-value	Pairwise identity
01	86	OLV2(122)	1.44E-08	35.1%
		YSLV4-ORF26(227)	3.40E-08	36.7%
02	216	Mavirus(165)	1.52E-10	33.1%
		OLV1	1.68E-07	25.3%
04	707	OLV(776)	4.65E-38	23.6%
		YSLV1-ORF04(766)	4.59E-24	25.3%
		Zamilon(778)	6.80E-21	29.8%
		V13(779)	7.51E-19	27.0%
		Sputnik 3(779)	7.51E-19	27.0%
06	729	YSLV4-ORF25(421)	1.31E-02	27.2%
11	199	Mavirus(165)	3.92E-04	43.8%

*aa means amino acid

Legends of supporting figure

Figure S1. Coverage of sequence assembly of four genomic sequences. Blue represents abundances of reads mapped onto the genomes. Numbers on the scale bar represent minimum and maximum sequences mapped onto the genome, respectively. Numbers on consensus and YSLPVs/YSLGV genomic sequences represent position of base pairs.

Figure S2. Encoding information of four YSL sequenes summarized from BLASTp results. Numbers indicate percentage of ORFans and non-ORFans in four genomic sequences.

Figure S3. Unrooted phylogenetic tree reconstructed using amino acid sequences of three conserved genes of VLTF3 (A), Topoisomerase II (B) and VV A32-like packaging ATPase (C) in YSLPVs, YSLGV and selected other giant virus families. YSLPVs and YSLGV are indicated in blue. Phycodnaviridae and Mimiviridae are marked with lines. Bootstrap values (100 iterations) are indicated on the branching of the tree.

Figure. S1

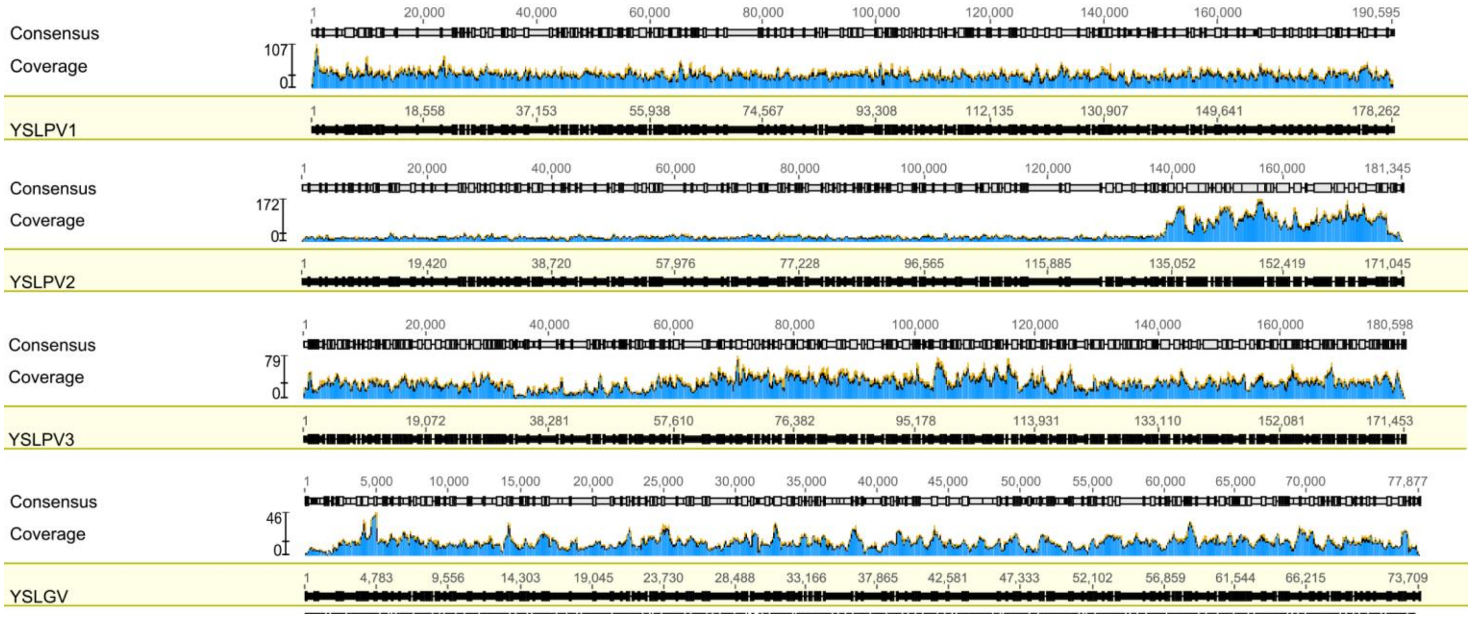
Consensus
Coverage
107
0
1 20,000 40,000 60,000 80,000 100,000 120,000 140,000 160,000 190,595
YSLPV1
1 18,558 37,153 55,938 74,567 93,308 112,135 130,907 149,641 178,262
Consensus
Coverage
172
0
1 20,000 40,000 60,000 80,000 100,000 120,000 140,000 160,000 181,345
YSLPV2
1 19,420 38,720 57,976 77,228 96,565 115,885 135,052 152,419 171,045
Consensus
Coverage
79
0
1 20,000 40,000 60,000 80,000 100,000 120,000 140,000 160,000 180,598
YSLPV3
1 19,072 38,281 57,610 76,382 95,178 113,931 133,110 152,081 171,453
Consensus
Coverage
46
0
1 5,000 10,000 15,000 20,000 25,000 30,000 35,000 40,000 45,000 50,000 55,000 60,000 65,000 70,000 77,877
YSLGV
1 4,783 9,556 14,303 19,045 23,730 28,488 33,166 37,865 42,581 47,333 52,102 56,859 61,544 66,215 73,709

Figure. S2

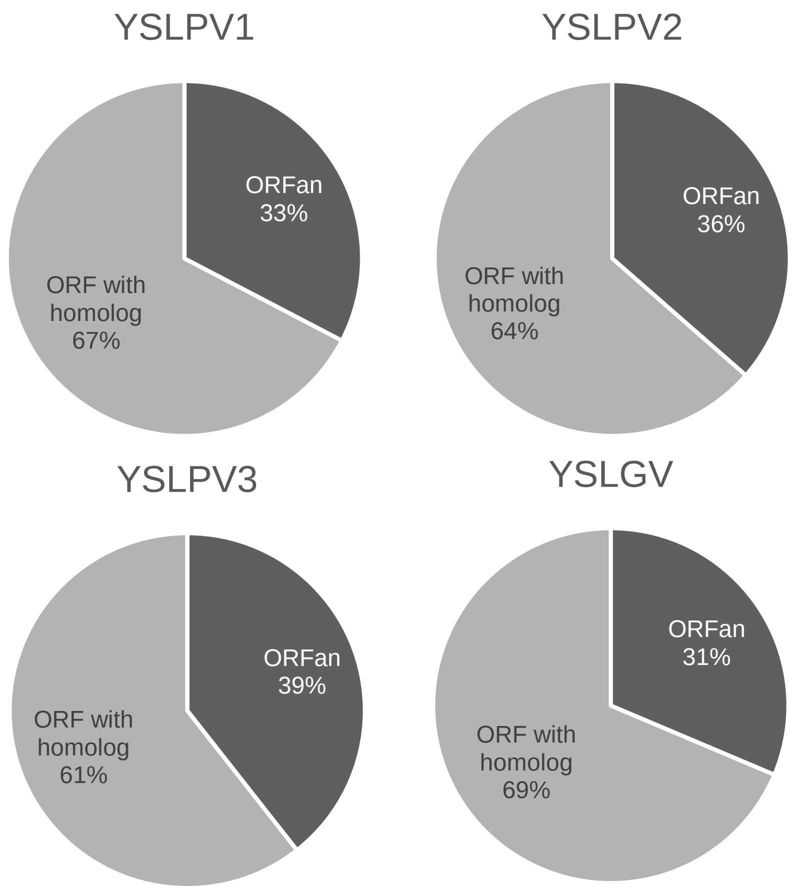
YSLPV1
ORFan
33%
ORF with
homolog
67%
YSLPV2
ORFan
36%
ORF with
homolog
64%
YSLPV3
ORFan
39%
ORF with
homolog
61%
YSLGV
ORFan
31%
ORF with
homolog
69%

Figure. S3

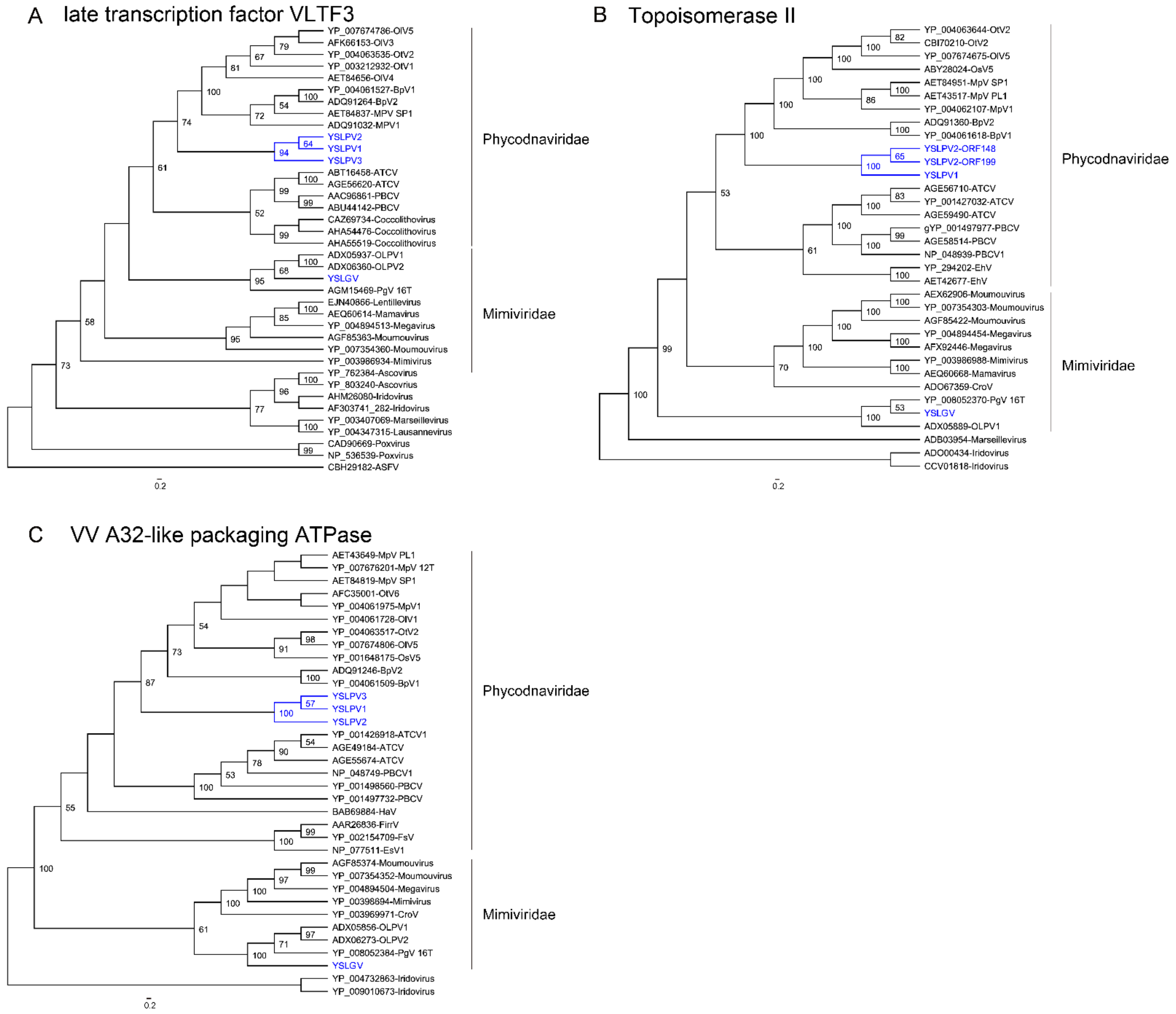
A late transcription factor VLTF3
YP_007674786-OlV5
AFK66153-OlV3
YP_004063535-OtV2
YP_003212932-OtV1
AET84656-OlV4
YP_004061527-BpV1
ADQ91264-BpV2
AET84837-MPV SP1
ADQ91032-MPV1
YSLPV2
YSLPV1
YSLPV3
ABT16458-ATCV
AGE56620-ATCV
AAC96861-PBCV
ABU44142-PBCV
CAZ69734-Coccolithovirus
AHA54476-Coccolithovirus
AHA55519-Coccolithovirus
ADX05937-OLPV1
ADX06360-OLPV2
YSLGV
AGM15469-PgV 16T
EJN40866-Lentillevirus
AEQ60614-Mamavirus
YP_004894513-Megavirus
AGF85363-Moumouvirus
YP_007354360-Moumouvirus
YP_003986934-Mimivirus
YP_762384-Ascovirus
YP_803240-Ascovrius
AHM26080-Iridovirus
AF303741_282-Iridovirus
YP_003407069-Marseillevirus
YP_004347315-Lausannevirus
CAD90669-Poxvirus
NP_536539-Poxvirus
CBH29182-ASFV
Phycodnaviridae
Mimiviridae
0.2
B Topoisomerase II
YP_004063644-OtV2
CBI70210-OtV2
YP_007674675-OlV5
ABY28024-OsV5
AET84951-MpV SP1
AET43517-MpV PL1
YP_004062107-MpV1
ADQ91360-BpV2
YP_004061618-BpV1
YSLPV2-ORF148
YSLPV2-ORF199
YSLPV1
AGE56710-ATCV
YP_001427032-ATCV
AGE59490-ATCV
gYP_001497977-PBCV
AGE58514-PBCV
NP_048939-PBCV1
YP_294202-EhV
AET42677-EhV
AEX62906-Moumouvirus
YP_007354303-Moumouvirus
AGF85422-Moumouvirus
YP_004894454-Megavirus
AFX92446-Megavirus
YP_003986988-Mimivirus
AEQ60668-Mamavirus
ADO67359-CroV
YP_008052370-PgV 16T
YSLGV
ADX05889-OLPV1
ADB03954-Marseillevirus
ADO00434-Iridovirus
CCV01818-Iridovirus
Phycodnaviridae
Mimiviridae
0.2
C VV A32-like packaging ATPase
AET43649-MpV PL1
YP_007676201-MpV 12T
AET84819-MpV SP1
AFC35001-OtV6
YP_004061975-MpV1
YP_004061728-OlV1
YP_004063517-OtV2
YP_007674806-OlV5
YP_001648175-OsV5
ADQ91246-BpV2
YP_004061509-BpV1
YSLPV3
YSLPV1
YSLPV2
YP_001426918-ATCV1
AGE49184-ATCV
AGE55674-ATCV
NP_048749-PBCV1
YP_001498560-PBCV
YP_001497732-PBCV
BAB69884-HaV
AAR26836-FirrV
YP_002154709-FsV
NP_077511-EsV1
AGF85374-Moumouvirus
YP_007354352-Moumouvirus
YP_004894504-Megavirus
YP_00398694-Mimivirus
YP_003969971-CroV
ADX05856-OLPV1
ADX06273-OLPV2
YP_008052384-PgV 16T
YSLGV
YP_004732863-Iridovirus
YP_009010673-Iridovirus
Phycodnaviridae
Mimiviridae
0.2
